# Diet Quality and Cancer Outcomes in Adults: A Systematic Review of Epidemiological Studies

**DOI:** 10.3390/ijms17071052

**Published:** 2016-07-05

**Authors:** Jennifer Potter, Leanne Brown, Rebecca L. Williams, Julie Byles, Clare E. Collins

**Affiliations:** 1 School of Health Sciences, Faculty of Health and Medicine, University of Newcastle, Callaghan 2308, Australia; Rebecca.Williams@newcastle.edu.au (R.L.W.); 2 Department of Rural Health, University of Newcastle, Tamworth 2308, Australia; Leanne.Brown@newcastle.edu.au; 3 Priority Research Centre for Physical Activity and Nutrition, University of Newcastle, Callaghan 2308, Australia; 4 Priority Research Centre for Gender, Health and Ageing, University of Newcastle, Callaghan 2308, Australia; Julie.Byles@newcastle.edu.au; 5 School of Medicine and Public Health, Faculty of Health and Medicine, University of Newcastle, Callaghan 2308, Australia

**Keywords:** cancer, diet quality index, risk and mortality, systematic review

## Abstract

Dietary patterns influence cancer risk. However, systematic reviews have not evaluated relationships between a priori defined diet quality scores and adult cancer risk and mortality. The aims of this systematic review are to (1) describe diet quality scores used in cohort or cross-sectional research examining cancer outcomes; and (2) describe associations between diet quality scores and cancer risk and mortality. The protocol was registered in Prospero, and a systematic search using six electronic databases was conducted through to December 2014. Records were assessed for inclusion by two independent reviewers, and quality was evaluated using a validated tool. Sixty-four studies met inclusion criteria from which 55 different diet quality scores were identified. Of the 35 studies investigating diet quality and cancer risk, 60% (*n* = 21) found a positive relationship. Results suggest no relationship between diet quality scores and overall cancer risk. Inverse associations were found for diet quality scores and risk of postmenopausal breast, colorectal, head, and neck cancer. No consistent relationships between diet quality scores and cancer mortality were found. Diet quality appears to be related to site-specific adult cancer risk. The relationship with cancer mortality is less conclusive, suggesting additional factors impact overall cancer survival. Development of a cancer-specific diet quality score for application in prospective epidemiology and in public health is warranted.

## 1. Introduction

Many types of cancer can be prevented or delayed. The World Cancer Research Fund (WCRF) and American Institute of Cancer Research (AICR) reported that 3–4 million cancer cases worldwide could be prevented through healthy lifestyle factors [1]. Despite public health efforts driven by research supporting the place of healthy lifestyle change for the prevention of cancer, it is expected that by 2020 the number of new cancer cases in Australia will rise by about 17% from this year’s 128,000 incident cases [2].

Whilst evidence supporting an inverse association between healthy dietary patterns, such as the Mediterranean and DASH diet patterns, and cancer incidence exists [1], little research to date reports on diet quality indices and cancer outcomes. Dissimilar to single-nutrient measures, diet quality indices measure quality and variety of the whole diet in order to capture food and nutrient intakes and interactions that are both protective and unfavourable [3]. Diet quality indices defined a priori are gaining favour in nutritional epidemiology as they enable dietary patterns of groups to be evaluated based on pre-existing guidelines, knowledge of favourable or unfavourable effects of various components of the overall dietary pattern, or both. Diet quality scores further allow research to focus on the whole diet rather than single nutrients and enable nutrients to be evaluated in the context of other dietary interactions. For example, a recent study used a diet quality score alongside other lifestyle scores to assess cohort alignment with the WCRF/AICR recommendations for cancer prevention [4].

Systematic reviews completed to date concentrate on specific cancer sites only and generally examine diet quality indices defined a posteriori, which are based exclusively on data obtained from the study population. Bertuccio et al. (2013) conducted a systematic review and meta-analysis examining the association been a posteriori and a priori defined dietary patterns and the incidence of stomach cancer [5]. Summarising data from 16 studies, seven of which reported on a priori diet quality scores, Bertuccio et al. reported an odds ratio (OR) for stomach cancer incidence ranging from 0.2 to 0.7 for the favourable diet quality scores and 1.8 to 6.9 for the unfavourable scores [5]. A meta-analysis by Magalhães et al. (2011) [6] reported a significant and positive association between colon cancer risk and a posteriori defined dietary patterns high in red and processed meat with colon cancer risk (*RR*_combined_ = 1.29, 95% CI: 1.13–1.48, *I*^2^ = 31.7%) and a significant inverse association with healthy dietary patterns (*RR*_combined_ = 0.80, 95% CI: 0.70–0.90, *I*^2^ = 55.1%) [6]. Brennan et al. (2010) similarly reported an association between a posteriori defined healthy dietary patterns and breast cancer incidence for the lowest compared to the highest category of healthy or prudent dietary patterns (OR = 0.89, 95% CI: 0.82–0.99; *p* = 0.02) [7]. Brennan et al. further found dietary patterns characterised by high alcohol intake to be associated with increased breast cancer risk (OR = 1.21, 95% CI: 1.04–1.41; *p* = 0.01) [7]. Additional literature reviews and studies support the place of healthy dietary patterns in the prevention of colorectal [8,9,10,11,12,13,14], breast [15,16,17], prostate [18], gastric [19], and head and neck cancer [20,21].

Good evidence links energy-dense, high-fat, and sugary foods with weight gain, whereas lower energy density diets can prevent overweight and obesity [22]. While the relationship between higher quality diets and risk of overweight and obesity is variable (Aljadani et al., 2013a; Aljadani et al., 2013b; Wolongevicz et al., 2010), alignment of dietary patterns with dietary guidelines has been shown to be associated with reduced risk of overweight and obesity in a number of cohort studies [23,24,25]. For example, a recent cross-sectional study by Aljadani et al., (2013) [23] calculated diet quality scores of young women aged 27.6 ± 1.7 years enrolled in the Australian Longitudinal Study on Women’s Health (*n* = 4287). After a follow-up of six years, those women with higher diet quality scores, as measured using the ARFS, and Australian Diet Quality Index (AUS-DQI), demonstrated less weight gain than those with lower scores. Given strong associations linking cancer incidence and body fatness, high diet quality has the power to influence cancer risk through weight management and the prevention of overweight and obesity.

## 2. Review Aims

The aim of this systematic review is to synthesise the best available evidence on the association between a priori defined diet quality scores and risk and mortality from cancer. Cancers of the colorectum, stomach, breast, prostate, head, and neck are of particular interest, as convincing evidence exists of a link between the risk of these cancers and diet quality [1].

## 3. Review Methods

According to the Preferred Reporting Items for Systematic Reviews and Meta-Analyses (PRIMSA), we systematically searched the literature for all prospective cohort and cross-sectional studies that investigated the association between cancer risk and/or mortality and a priori diet quality score(s). The review protocol was peer reviewed and registered with the online Prospero database [26]. This protocol can be accessed from the University of York – Centre for Reviews and Dissemination [27].

Table 1 outlines the inclusion and exclusion criteria. The outcomes measured in this review will be risk and mortality from total cancer and risk of colorectal, gastric, breast, prostate, and head and neck cancer. Risk estimates will be relative to diet quality score and will include relative risk (RR), hazard ratio (HR), and odds ratio (OR).

### 3.1. Search Strategy

A systematic review of the literature was conducted in 2014 to find and retrieve both published and unpublished studies in the English language with no date limits. A three-step search strategy was followed for this review. An initial search was carried out using the Medline and CINAHL online databases to analyse title and abstract key words and identify index terms (MeSH headings) used to identify articles. The second search used key words and index terms across all included databases. Databases searched included MEDLINE, Medline in Process, EMBASE, CINAHL, and Scorpus. The database Proquest (Dissertations and Theses) was used to find non-commercially published articles. To identify the outcome of interest, the following keywords were searched: neoplasms (exp/), breast neoplasms (exp/), gastric neoplasms (exp/), colonic neoplasms (exp/), rectal neoplasms (exp/), prostatic neoplasms (exp/), and head and neck neoplasms (exp/). To identify diet quality as the key exposure variable of interest the following key words were searched: diet variety (diet* adj3 variet*), diet quality (diet* adj3 quality), diet score (diet* adj1 score$), dietary pattern (diet* adj1 pattern$), diet diversity (diet* adj1 diversity$), and diet index (diet* adj1 ind$). Keywords were searched in the title, abstract, and body of electronic records. Dietary and outcome keywords were searched with the Boolean operator “AND” to identify records. Additional records were added following search of references on records found using the keyword search.

**Figure 1:**
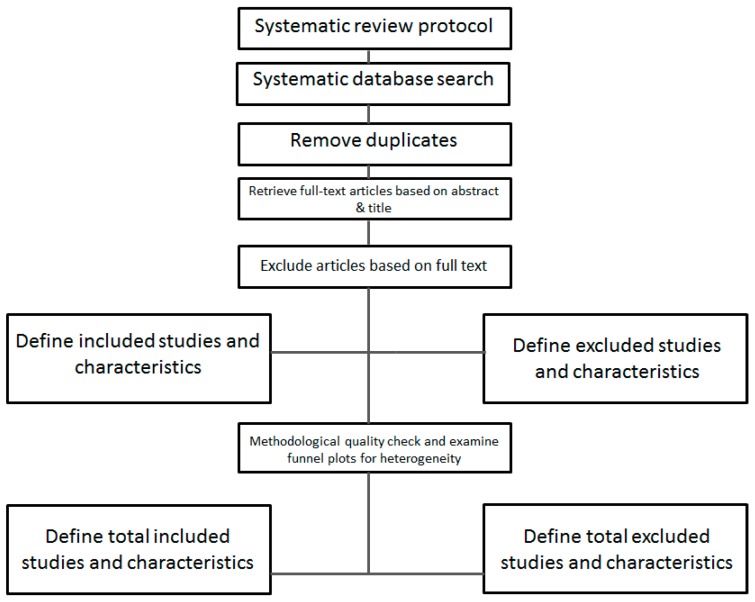
Systematic review flow chart.

The search strategy is shown in the flow chart in Figure 1.

### 3.2. Eligibility Criteria

Inclusion and exclusion criteria are shown in Table 1.

### 3.3. Selection Process

All records retrieved were assessed for eligibility by two independent researchers based on the title, abstract, and MESH headings. The full-text articles appearing to meet eligibility criteria in this early stage were then retrieved. Full-text records were then compared to the relevant inclusion and exclusion criteria (see Table 1) to determine which may be included in the review. When the reviewers disagreed on a study’s relevance for inclusion, a third independent reviewer was consulted. Where multiple studies are identified from the same dataset, the datasets were combined where appropriate or the largest dataset included in the review.

### 3.4. Quality Assessment of Included Studies

Studies were evaluated for methodological quality by a single author using a validated 10-item tool. Criteria assessed included sample selection methods and sample generalisability; study blinding; description and measurement of the exposure; outcome ascertainment and measurement; statistical analysis; funding sources; and author declarations. Four of the pre-defined quality criteria were predesignated as “essential” and needed to be met for a certain study to be assigned a high quality rating: (1) sample selection; (2) comparability of “exposed” and “not exposed” groups; (3) description and measurement of the exposure; and (4) outcome ascertainment and measurement. An overall quality classification “negative”, “neutral”, or “positive” was awarded to each study. Studies were classified as “positive” if five from the ten criteria were met including all four “essential” criteria. If the majority of the 10 criteria were met, but one of the “essential” criteria were not met, a study was assigned a “neutral” classification. If less than five criteria were met or less than two essential criteria were not met, a study was assigned a “negative” classification. When provided detail was insufficient to enable a criterion to be assessed adequately, this criterion was assigned an unclear classification. No studies were excluded based on quality ratings.

An example of the quality assessment tool used for this study is found in Appendix A.

## 4. Results

### 4.1. Description of Studies

**Figure 2:**
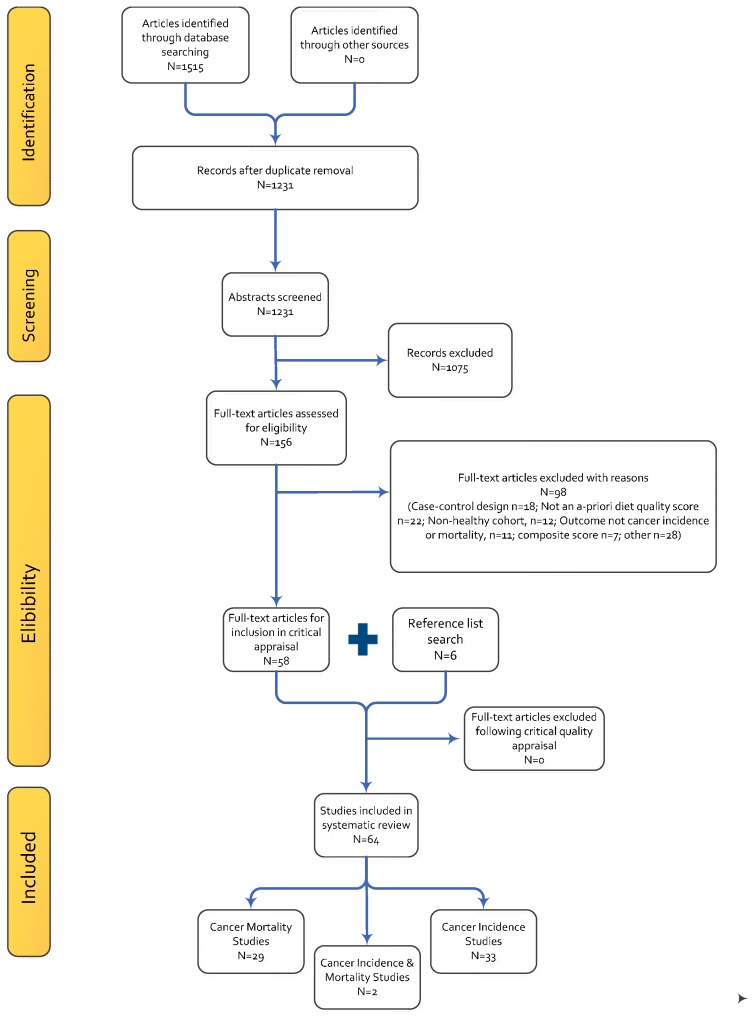
Flow diagram showing the method of determining studies to be included in the systematic review.

The process for study inclusion is presented in Figure 2. Following the initial database search, 1515 citations were retrieved after duplicate removal. Following the title and abstract review, 1231 records were excluded. Of the 156 full-text articles, screened based on the eligibility criteria, 98 were excluded to leave a total of 58 articles available for the systematic review. The major reasons for study exclusion were study design (case-control *n* = 18) and studies using an a posteriori or factor-analysis approach for diet quality assessment (*n* = 22). After screening of the reference lists of the included studies, six additional articles were added to the systematic review to result in a final total of 64 studies (Table 2). All studies were classified as being “positive” (*n* = 46) or “neutral” (*n* = 18) quality using the predefined quality tool. Quality aspects least well reported were study selection, handling of withdrawals or attrition, and blinding. Blinding of the investigators to exposure and outcomes within the study groups was not addressed or “unclear” in 73% of the included studies (*n* = 47) and the source of study funding was not reported in four articles. Publication years of the articles ranged from 1995 to 2014, with an increasing number of studies published after 2010 (44/64). All included studies were of prospective cohort design.

Twenty-eight studies were conducted in the United States of America [11,12,13,18,20,29,39,42,45,46,48,51,52,53,54,55,61,62,63,65,66,67,69,70,71,76,77,81], *n* = 16 conducted in Northern Europe [28,30,31,38,41,43,50,57,58,59,68,73,74,75,78,83], *n* = 6 in Southern Europe [32,36,64,79,80,82], and *n* = 10 across Northern and Southern Europe [10,17,33,34,35,37,40,47,49,56]. Other studies were conducted in regions including Canada [44], Australia [84], Taiwan [60], and Japan [72] (Table 2). The sample size across the studies ranged from *n* = 1044 [30] to *n* = 537,218 [29] and the majority (>75%) of studies had greater than 20,000 participants. The mean follow-up period of the included studies was 13 years and ranged from 3.7 years to 26 years. Food frequency questionnaires (FFQs) (*n* = 52) and 24-h recalls (*n* = 6) were the most common dietary data collection method used to derive a diet quality score. The number of FFQ items ranged from 18 to more than 200 items. Some methodologies required a combination of dietary data collection methods to derive a diet quality score (e.g., diet history and seven-day food record).

Data from the European Prospective Investigation into Cancer (EPIC) were used in 14 studies [10,17,31,32,33,34,35,36,37,40,49,79,80,83], while the American National Institute of Health-American Association of Retired Persons Nutrition and Health (NIH-AARP) cohort data was used in *n* = 11 studies [13,18,20,29,48,53,61,62,69,70,76]. The American-based NHS data were used in eight studies [11,12,39,45,46,66,67,81], the American-based HPFS data were used in *n* = 6 studies [11,39,46,55,66,67], and data from the Swedish Västerbotten Intervention Project (VIP) cohort were used in *n* = 4 studies [73,74,75,78] (Table 2).

### 4.2. Diet Quality Scores

Of the 64 included studies, 55 different diet quality indices were identified as having been used to assess the association between diet quality and cancer outcomes. Supplementary Table S1 describes the diet quality indices and includes tools developed in the United States (*n* = 29), Sweden (*n* = 8), the United Kingdom (*n* = 6), Greece (*n* = 2), and Taiwan (*n* = 2). One diet quality score was taken from each of the following: Canada, Japan, Germany, Australia, the Netherlands, and Denmark. A final diet quality score was created for use in a combined cohort from the Netherlands, Italy, and Finland. The predominant method of dietary assessment for derivation of the scores was a food frequency questionnaire (FFQ) (*n* = 42) with a smaller number of scores derived from food records (*n* = 3) and 24-h recalls (*n* = 6). Three studies used a combination of FFQ and food records to derive a diet quality score. The majority of scores were food- and nutrient- (*n* = 25) or food-based (*n* = 24), with a smaller number nutrient-based only (*n* = 6).

Whilst a score or index may have been developed for a specific population or for a specific purpose, many have been used numerous times with diverse population groups and purposes. For example, the DASH index was originally developed to measure adherence to the Dietary Approaches to Stop Hypertension eating pattern, which was originally developed based on heart health guidelines; however, a number of studies [11,69,76] have used this score to examine a relationship with cancer incidence or mortality. The majority of scores included in this review are updated or repatriated versions of an original score, for example the Healthy Eating Index-2005 (HEI-2005) and Healthy Eating Index-2010 (HEI-2010) are updated versions of the original Healthy Eating Index (HEI), which was based on the 1992 USDA Food Guide Pyramid and the 1995 Dietary Guidelines for Americans. The HEI is a food- and nutrient-based score and awards points based on intake from the five food groups (grains, fruit, vegetables, dairy, meats, and alternatives) as well as an adequate intake of key nutrients (cholesterol, sodium, total and saturated fat), and a final point is awarded for dietary variety. A total of eight updates or variations of the HEI are used in the studies included in this review.

A number of variations of the Mediterranean diet score (MDS) are also included in this review (*n* = 11). The Mediterranean dietary pattern is based on the prevalent traditional diets of peoples observed in the olive growing regions of the Mediterranean region and is characterised by a high intake of (i) vegetables; (ii) legumes; (iii) fruits and nuts; (iv) whole grain cereals; a moderate intake of (v) dairy foods; (vi) fish; a moderate-high intake of (vii) mono-unsaturated lipids versus saturated lipids; a low intake of (vii) meat and processed meat products; and a regular (ix) moderate alcohol intake. The original MDS was published by Trichopoulou et al. (1995) [85] and was designed for a Greek population. Updates or repatriations of the MDS often involve variations of the score to suit a different population. For example, the modified MDS (MMDS) was developed by Reedy et al. (2008) [13] to be used in the American NIH-AARP cohort. Other variations of the score have removed or changed certain components to reflect a specific purpose. For example, Buckland et al. (2013) [17] excluded the alcohol component from the score when examining the relationship between the Mediterranean diet and breast cancer risk, as alcohol is a known factor in breast cancer incidence.

The Recommended Food Score (RFS) and variations thereof is included in six studies included in this review. Similar to the HEI but based on foods only rather than foods and nutrients, the RFS is based on the 1995 Dietary Guidelines for Americans and scores the intake of 23 different foods across a weekly eating pattern. Variations of the RFS in this review have been designed to reflect different FFQ dietary measurement tools [50,67] and changes to suit non-American populations [68].

Five scores in this review define diet quality as adherence to a low-carbohydrate- and high protein-eating pattern (LCHP). Variations of this score comprise indices based on the proportion of total daily energy intake derived from carbohydrate and protein. An additional two scores developed by Fung et al. (2010) [46] further stratify the LCHP score to an animal-based LCHP score (LCHP-AB) and a vegetable-based LCHP score (LCHP-V).

Four different variations of the original DASH and Diet Diversity (DDS) scores respectively are found in studies included in this review. The original DASH diet index was based on the foods and nutrients emphasised and minimised in the Dietary Approaches to Stop Hypertension eating LCHP-V pattern. The DASH score is based on the intake from five components considered to be cardio-protective (fruits; vegetables; nuts and legumes; low fat dairy; and wholegrains) and three considered to be detrimental to cardiovascular health (sodium; processed meat; and sweetened beverages). The four variations of the DASH score included in this review reflect differences in scoring methods to obtain a final overall DASH score. Variations of the Diet Diversity Scores (DDS) consider dietary quality as a function of variation both within and between food groups independent of quantities. Four different DDS variations are found in studies included in this review [33,34,49,51,60], and differences arise predominantly from specific scoring and dietary data collection tools. Overall, DDS scores in this review allocate higher points for greater intake of different foods from within major food groups over the preceding day or two-week period.

Three variations respectively of the Diet Quality Index (DQI) and Healthy Diet Indicator (HDI) are also found in studies included in this review. The DQI was originally developed by Patterson et al. (1994) to reflect the quality of the diet based on the US National Research Council Diet and Health Recommendations for prevention of chronic disease. The DQI is both food- and nutrient-based and comprises eight components which are scored dichotomously (percent energy from fat and saturated fat; cholesterol intake; fruit and vegetable intake; breads, cereal, and legume intake; protein intake; sodium intake; and calcium intake). Adaptations of the DQI in this review include variations for certain nutrients [42,77] and for certain populations. The HDI was originally developed to reflect a dietary pattern based on the WHO Diet, Nutrition and the Prevention of Chronic Diseases report [47]. The HDI is a dichotomous food- and nutrient-based score that comprises 7 to 10 components. Variations of the score reflect chronological updates to the WHO report and adaptations to different populations and diet assessment methods.

A number of additional scores (*n* = 11) are included in this review that reflect region-specific dietary guidelines (DQI-Swedish Nutrition Recommendations; Nordic Food Index; Traditional Sami Diet Score; German Food Pyramid Index; and Aussie DQI), and/or novel classifications of diet quality (Ideal Diet Index; Overall Dietary Index-Revised; and Diet Behaviour Score). Two variations of the RFS, the non-RFS (1) and non-RFS (2), are also negatively scored and reflect intake of foods considered detrimental to health.

### 4.3. Diet Quality and Cancer Risk

Studies examining the relationship between diet quality and cancer risk (*n* = 35) are shown in Supplementary Table S2.

A total of 12 studies used data from the European Investigation Into Cancer data set [10,17,31,32,33,34,35,37,41,49,80,83]. Data from the National Institute of Health-American Association of Retired Persons Nutrition and Health (NIH-AARP) cohort were used by another seven studies [18,20,29,48,61,62,69]. Four studies used data from the Nurse’s Health Study (NHS) and Health professionals Follow-Up Study (HPFS) cohorts [11,39,67,86]. Three studies used NHS cohort data only [12,45,66], whereas one study uses only HPFS cohort data [65]. A number of other cohorts were also used in other studies in this review including data from the Breast Cancer Detection and Demonstration Project (BCDDP) [63], Denmark’s Diet, Cancer and Health study [57], the Nova Scotia Nutrition Survey [44], the Swedish Women’s Lifestyle and Health (WLH) cohort [40], the UK Women’s Cohort Study (UKWCS) [38], Uppsala Longitudinal Study of Adult Men (ULSAM) [30], and the Västerbotten Intervention Project (VIP) [75].

### 4.4. Overall Cancer Risk

Overall, 11 studies investigated the relationship between incidence of cancer of any site and diet quality [31,32,39,40,44,63,65,66,75,83]. Of the 11 studies investigating the link between incidence of any cancer and diet quality scores, three studies (27%) reported that better scores were associated with decreased risk [32,39,40], with two of these three [32,40] measuring adherence to the Mediterranean diet. Benetou et al. (2008) [32] reported a relationship between higher scores of the MMDS and reduced total cancer risk amongst 25,623 Greek men and women (HR_(Tertile 3_ _vs. Tertile 1)_ = 0.78, 95% CI: 0.64, 0.94) enrolled in EPIC. After stratification by sex, Benetou et al. observed a stronger association between higher MMDS scores and reduced cancer risk for women (HR_(Tertile 3_ _vs. Tertile 1)_ = 0.73, 95% CI: 0.56, 0.96) when compared to men (HR_(Tertile 3_ _vs. Tertile 1)_ = 0.83, 95% CI: 0.63, 1.09). Couto et al. (2011) [40] also measured diet quality using the MMDS and its relationship to overall cancer incidence in men and women enrolled in EPIC (*N* = 478,478). Estimating that a respective 4.7% and 2.4% of cancers may be prevented in men and women who follow a Mediterranean dietary pattern, higher MMDS scores were significantly associated with reduced cancer incidence in this EPIC cohort (HR_(High_ _vs. Low scores)_ = 0.93, 95% CI: 0.89, 0.96. P trend 0.0001 for women; (HR_(High_ _vs. Low scores)_ = 0.93, 95% CI: 0.88, 0.99. P trend 0.02). Chiuve et al. (2012) [39] found a 10% and 6% reduction in cancer risk among 112,524 men and women as measured by the HEI-2005 and AHEI-2010, respectively. After stratification by gender, the HEI-2005, which is based on the MyPyramid and 2005 Dietary Guidelines for Americans, was found to be associated with decreased all-sites cancer risk in both men (RR_(Quintile 1_ _vs. Quintile 5)_ = 0.86, 95% CI: 0.64, 0.98. P trend 0.003) and women (RR_(Quintile 1_ _vs. Quintile 5)_ = 0.93, 95% CI: 0.87, 0.98. P trend 0.04) [39]. Higher scores of the AHEI-2010, derived from recommendations for chronic disease prevention, were further found to be associated with reduced cancer risk in women (RR_(Quintile 1_ _vs. Quintile 5)_ = 0.93, 95% CI: 0.88, 0.99. P trend 0.01) but not men (RR_(Q1__vs. Q5)_ = 0.94, 95% CI: 0.87, 1.03. P trend 0.13) [39]. Using 24-h recall data from a small sample of men and women who participated in the 1990 Nova Scotia Nutrition Survey (*n* = 2108), Fitzgerald et al. (2002) [44] estimated that cancer incidence could be reduced by approximately 35% through improving the diet quality of participants. Measured using a nutrient-based a priori score based on the 2001 American and Canadian Dietary Reference Intakes (DRIs), Fitzgerald et al. (2002) reported associations between higher diet quality and reduced cancer incidence in men (OR_(Quartile_ _vs. Quartile 1)_ = 0.81, 95% CI: 0.40, 1.64) and women (OR_(Quartile4_ _vs. Quartile 1)_ = 0.94, 95% CI: 0.44, 2.00), but these relationships did not reach statistical significance (P linear trend 0.41 and 0.54 for men and women, respectively).

Of the eight studies reporting no relationship between diet quality scores and all-sites cancer incidence, two studies used the Recommended Food Score (RFS) [63,67], two studies used a variation of the Healthy Eating Index (HEI-f) [65,66], and other studies used the Alternate Healthy Eating Index (AHEI) [67], WHO HDI [31], LCHP score [75], and GFPI [83].

Mai et al. (2005) [63] investigate the association between the Recommended Food Score (RFS), which measures diet quality based on the 1995 US Dietary Guidelines, and risk of all-sites cancer in 42,254 women enrolled in the Breast Cancer Detection and Demonstration Project (BCDDP). After a mean follow-up of 9.5 years, Mai et al. found no association between the RFS and risk of all-sites cancer (HR_(Quartile 4_ _vs. Quartile 1)_ = 0.98, 95% CI: 0.88, 1.09; P trend 0.99). In two separate studies, McCullough et al. (2000a) [65] and McCullough et al. (2000b) [66] investigated the association between the risk of all-sites cancer and the HEI-f in men and women enrolled in the HPFS (*n* = 38,622) and NHS (*n* = 67,272) cohorts, respectively. A measure of diet quality based on the 1995 Dietary Guidelines for Americans and calculated from FFQ data, the HEI-f was not associated with risk of cancer in men after an eight-year follow-up (RR_(Quintile 5_ _vs. Quintile 1)_ = 1.12, 95% CI: 0.95, 1.31; P trend 0.27) or women after a 12-year follow-up (RR_(Quintile 5_ _vs. Quintile 1)_ = 1.02, 95% CI: 0.93, 1.121; P trend 0.578). A later investigation from McCullough et al. (2002) [67] updated the HEI-f to include dietary factors associated with reducing chronic disease risk (Alternate Healthy Eating Index, AHEI see Table S1) and included in the evaluation of associations with cancer risk the food-based recommended food score (RFS). Collecting FFQ data at baseline from each of the NHS (*n* = 67,271 women aged 30–55 years at baseline in 1976) and HPFS (*n* = 38,615 men aged 40–75 years at baseline in 1986) cohorts, McCullough et al. (2002) found no association between either the AHEI or RFS and risk of cancer in men or women after an 8–12-year follow-up.

Presenting data from two cohorts within the Dutch segment of EPIC (*n* = 35,355 men and women), Berentzen et al. (2013) [31] found no association between cancer risk and higher scores of the WHO Healthy Diet indicator (HDI) (HR_(Tertile 3_ _vs. Tertile1)_ = 0.99, 95% CI: 0.96, 1.02. P trend 0.53) which was based on favourable intakes of saturated fat, polyunsaturated fat, cholesterol, protein, dietary fibre, free sugars, fruits, and vegetables (excluding potato). After stratification by gender and separate examination of the risk of smoking- and alcohol-related cancers by HDI scores, Berentzen et al.’s null result remained. Nilsson et al. (2013) [75] investigated the association between total cancer incidence and a LCHP score (LCHP, see Table S1) among 62,582 men and women enrolled in the Västerbotten Intervention Project (VIP). Followed for up to 17.8 years, higher LCHP scores were not associated with cancer risk in men (HR_(High_ _vs. Low scores)_ = 0.97, 95% CI: 0.97, 1.03; P trend 0.973) or women (HR_(High_ _vs. Low scores)_ = 1.00, 95% CI: 0.85, 1.15; P trend 0.777) from the VIP cohort. Using EPIC-Potsdam data, von Ruesten et al. (2010) [83] investigated the association between all-sites cancer incidence and a diet quality index designed to align with the German Food Pyramid (GFPI, see Table S1). After a mean follow-up of 8.7 years, von Reusten et al. reported no association between higher GFPI scores and risk of cancer in either men (HR_(Quintile 5_ _vs. Quintile 1)_ = 1.16, 95% CI: 0.83, 1.62; P trend 0.4015) or women (HR_(Quintile 5_ _vs. Quintile 1)_ = 0.79, 95% CI: 0.58, 1.08; P trend 0.1444).

### 4.5. Breast Cancer Risk

Of the studies (*n* = 8) investigating the relationship between diet quality and breast cancer risk in adult women, four reported relationships where higher diet scores were associated with lower risk [12,17,45,80]. Assessing adherence to the Mediterranean dietary pattern using an adapted relative variation of the MDS (arMED, see Table S1), which excludes alcohol, Buckland et al. (2013) found a 6% overall reduction in breast cancer risk among 335,062 women enrolled in EPIC with high scores (HR_(High_ _vs. Low scores)_ = 0.94; 95% CI: 0.88, 1.00. P trend 0.048). After stratification by menopausal status, Buckland et al.’s association remained significant only for breast cancers diagnosed among postmenopausal women (HR _(High_ _vs. Low scores)_ = 0.93; 95% CI: 0.87, 0.99 for postmenopausal tumours; HR_(High_ _vs. Low scores)_ = 0.97; 95% CI: 0.81, 1.15. P trend 0.839 for tumours diagnosed prior to menopause). Using data available on breast cancer tumour receptor status, Buckland et al. further reported that, for tumours lacking oestrogen and progesterone receptors, higher adherence to the arMED reduced risk by 20% among postmenopausal women. Using data from the EPIC Greek segment (*n* = 14,807 women aged 20–86 at baseline 1994–8), Trichopoulo et al. (2010) [80] investigated the association between breast cancer risk and adherence to a Mediterranean dietary pattern (MMDS). After a 9.8-year follow-up, Trichopoulou et al. reported an association between higher MMDS scores and reduced postmenopausal (HR_(High_ _vs. Low scores)_ = 0.59, 95% CI: 0.34, 1.03; P trend 0.03) but not premenopausal breast cancer incidence (HR_(High_ _vs. Low scores)_ = 1.13, 95% CI: 0.69, 1.85; P trend 0.91). Using data from 86,621 postmenopausal women enrolled in the NHS, Fung et al. (2011) [12] also found a relationship between higher diet quality scores, based on the DASH dietary pattern, and lower breast cancer risk (RR_(Quintile 5_ _vs. Quintile 1)_ = 0.97 (95% CI: 0.89, 1.06. P trend 0.98). This inverse relationship observed by Fung et al. was strengthened with stratification by receptor status with oestrogen receptor negative (ER−) tumours showing a significant relationship (RR_(Quintile 5_ _vs. Quintile 1)_ = 0.80, 95% CI: 0.64, 1.01. P trend 0.02) with DASH scores whereas no significant association observed for oestrogen receptor positive (ER+) tumours (RR_(Quintile 5_ _vs. Quintile 1)_ = 0.96, 95% CI: 0.85, 1.08. P trend 0.89). Further, whilst no association was found for total LCHP scores, Fung et al. (2011) found an association between a higherLCHP-V score (see Table S1) and lower ER− postmenopausal breast cancer risk (HR _(Quintile 5_ _vs. Quintile 1)_ = 0.81, 95% CI: 0.65, 1.01. P trend 0.03). An earlier study, also by Fung et al. (2006), similarly used NHS (*n* = 71,058 women aged 34–55 years) data to report associations between five different diet quality scores and postmenopausal breast cancer risk (HEI-f, AHEI, RFS, aMED, and DQIR, see Table S1). This earlier study found no association between any of the aforementioned diet quality scores and overall postmenopausal breast cancer risk. However, after stratification by receptor status, a significant relationship between risk of ER− tumours and diet quality but not for ER+ tumours was found. Fung et al.’s 2006 study [45] reported associations between a lower risk of ER− tumours and higher diet quality as measured by the AHEI (RR_(Quintile 5_ _vs. Quintile 1)_ = 0.78, 95% CI: 0.59, 1.04. P trend 0.01), the RFS (1) (RR_(Quintile 5_ _vs. Quintile 1)_ = 0.69, 95% CI: 0.51, 0.94. P trend 0.003), and an alternate MDS(aMED) (RR_(Quintile 5_ _vs. Quintile 1)_ = 0.79, 95% CI: 0.60, 1.03. P trend 0.03). No association was found for risk of ER− tumours and diet quality as measured by the HEI-f (RR_(Quintile 5_ _vs. Quintile 1)_ = 0.92, 95% CI: 0.68, 1.24. P trend 0.47) and DQIR (RR_(Quintile 5_ _vs. Quintile 1)_ = 0.97, 95% CI: 0.72, 1.31. P trend 0.35).

Of the four studies finding no association between diet quality and breast cancer risk, two used variations of the MDS [38,40], one used the RFS [63], one the HDI [38], and another the LCHP score [75]. Using data from 33,731 women enrolled in the UKWHS, Cade et al. (2011) [38] reported no association between breast cancer risk and diet quality as measured using the MMDS (HR_(High_ _vs. Low scores)_ = 0.96, 95% CI: 0.70, 1.32) and HDI (HR_(High_ _vs. Low scores)_ = 0.94, 95% CI: 0.67, 1.32). Whilst Cade et al. [38] reported no significant findings by menopausal status, no data was available for this cohort on tumour receptor classification. Couto et al.’s (2013) [41] findings of a null association with breast cancer risk amongst 49,258 young women enrolled in the Swedish WLH cohort (RR_(High_ _vs. Low scores)_ = 1.42, 95% CI: 0.99, 2.03. P trend 0.12), also measuring diet quality using the MMDS, are similar to those of Cade et al. (2011) [38]. This finding of a null association was not changed when results were stratified by menopausal status, tumour receptor status, malignancy grade, tumour histology, tumour stage, and invasiveness. Couto et al. [41] further investigated the relationship between MMDS and breast cancer risk, excluding the alcohol component from the diet quality score. Couto et al. again found no significant association with breast cancer risk in the cohort of young Swedish women (RR_(High_ _vs. Low scores)_ = 1.06, 95% CI: 0.98, 1.13). Mai et al. (2005) [63] investigated the association between breast cancer risk and the RFS in 42,254 women (µ age 61 years) enrolled in the Breast Cancer Detection and Demonstration Project (BCDDP) follow-up cohort. After a median follow-up of 9.5 years, no association between RFS scores and breast cancer incidence (RR_(Quartile 4_ _vs. Quartile 1)_ = 1.05, 95% CI: 0.90, 1.23. P trend 0.81) was found. Mai et al. [63] did not investigate menopausal status by cancer risk or tumour receptor status. Using data from the VIP, Nilsson et al. (2013) [75] investigated the association between a LCHP score and risk of breast cancer in 31,185 adult women. Followed for up to 17.8 years, women with high LCHP scores, compared to women with low LCHP scores, did not show any increase or decrease in the risk of breast cancer incidence (HR_(High_ _vs. Low scores)_ = 1.00, 95% CI: 0.79, 1.27; P trend 0.924). Data on menopausal history or tumour receptor status were not available from this study.

### 4.6. Colorectal Cancer Risk

All but one of the eight studies investigating the association between diet quality and colorectal cancer (CRC) reported a relationship between higher diet scores and lower CRC risk. Of these seven studies, three used variations of the MDS [10,11,13], two used adaptations of the DASH index [46,69]; two used the RFS [13,63], two used HEI variants [13,48], and one other used the region-specific Nordic Food Index [57].

As the only study of the six to use EPIC data, Bamia et al. (2013) [10] reported a significant association between a higher modified Mediterranean diet score (MMDS) and an EPIC centre-specific MMDS (CSMMDS) and reduced CRC risk in 480,308 European men and women (HR _(High_ _vs. Low scores)_ = 0.89, 95% CI: 0.80, 0.99. P trend 0.02 for MMDS, and HR _(High_ _vs. Low scores)_ = 0.92, 95% CI: 0.84, 1.00. P trend 0.05 for CSMMDS). After stratification by sex, the CSMMDS became non-significant for both genders, whereas the MMDS remained significant for women (HR_(High_ _vs. Low scores)_ = 0.88, 95% CI: 0.77, 1.01. P trend 0.05) but not men (HR _(High_ _vs. Low scores)_ = 0.89, 95% CI: 0.76, 1.04. P trend 0.14). Using data from the NHS (*n* = 87,256 women) and HPFS (*n* = 45,490 men), Fung et al. (2010) [11] also found a significant relationship between lower CRC risk and greater adherence to a DASH index, which involved a higher intake of whole-grains, fruits, and vegetables; moderate intakes of low-fat dairy; a lower intake of processed meats, sweetened beverages, and desserts. Using a pooled analysis of both men and women, a 20% reduction in CRC risk among those in the highest quintile versus the lowest quintile of adherence to the DASH index (RR = 0.80, 95% CI: 0.70, 0.91. P trend 0.001) but not aMED (RR = 0.89, 95% CI: 0.77, 1.01. P trend 0.06) was reported. After stratification by sex, Fung et al. [11] further found a stronger association for higher adherence to the DASH index and lower CRC risk in women (RR _(Quintile 5_ _vs. Quintile 1)_ = 0.80, 95% CI: 0.67, 0.94. P trend 0.005) compared to men (RR _(Quintile 5_ _vs. Quintile 1)_ = 0.81, 95% CI: 0.66, 1.00. P trend 0.09). Using NIH-AARP data, Jarvandi et al. (2013) [48] found an association between higher HEI-2005 scores and lower risk of CRC in 484,020 men and women followed for an average of 9.2 years (HR_(Quartile 4_ _vs. Quartile 1)_ = 1.35, (95% CI: 1.26, 1.44). Miller et al. (2013) [69] also reported associations between higher diet quality, as measured using four variations of a DASH dietary pattern index and lower CRC risk using data from adult men and women enrolled in the NIH-AARP study (*n* = 491,841). After an 11-year follow-up, Miller et al. reported a significantly reduced risk of up to 25% for CRC in men using all four DASH index variations: Dixon (HR _(High_ _vs. Low scores)_ = 0.77, 95% CI: 0.69, 0.87), Mellen (HR _(Quintile 5_ _vs. Quintile 1)_ = 0.78, 95% CI: 0.71, 0.86), Fung (HR _(Quintile__5__vs. Quintile 1)_ = 0.75, 95% CI: 0.68, 0.83), and Güenther (HR _(Quintile 5_ _vs. Quintile 1)_ = 0.81, 95% CI: 0.74, 0.90) (P trend < 0.05 for all comparisons). Miller et al. [69] also reported significantly reduced risks of CRC among women of up to 21% for the Mellen (HR _(Quintile 5_ _vs. Quintile 1)_ = 0.79, 95% CI: 0.68, 0.91), Fung (HR _(Quintile 5_ _vs. Quintile 1)_ = 0.84, 95% CI: 0.73, 0.96), and Güenther (HR _(Quintile 5_ _vs. Quintile 1)_ = 0.84, 95% CI: 0.73, 0.97) scores but not for the Dixon (HR _(High_ _vs. Low scores)_ = 1.01, 95% CI: 0.80, 1.28) variation. Reedy et al. (2008) [13], using NIH-ARRP data (*n* = 492,382 men and women), found a significantly reduced risk of CRC in men (*n* = 293,615) after a five-year follow-up, with higher diet quality scores as measured by the HEI-2005 (HR _(Quintile 5_ _vs. Quintile 1)_ = 0.72, 95% CI: 0.62, 0.83), AHEI (HR _(Quintile 5_ _vs. Quintile 1)_ = 0.71, 95% CI: 0.61, 0.82), MDS (1) (HR _(Quintile 5_ _vs. Quintile 1)_ = 0.72, 95% CI: 0.63, 0.83), and RFS (1) (HR _(Quintile 5_ _vs. Quintile 1)_ = 0.75, 95% CI: 0.65, 0.87). Reedy et al. [13] repeated the same analysis in women (*n* = 198,767) and found only a significant association with CRC risk for the HEI-2005 (HR _(Quintile 5_ _vs. Quintile 1)_ = 0.80, 95% CI: 0.64, 0.98) and borderline significance for the AHEI (HR _(Quintile 5_ _vs. Quintile 1)_ = 0.83, 95% CI: 0.66, 1.05). Using data from the BCDDP follow-up cohort, Mai et al. (2005) [63] similarly reported a non-significant association between high RFS scores and a 16% reduced risk of CRC in a cohort of 42,254 women (RR _(Quartile 4_ _vs. Quartile 1)_ = 0.84, 95% CI: 0.62, 1.14. P trend 0.18). Kyro et al. (2013) [57] investigated the relationship between a healthy Nordic Food Index and risk of CRC in men and women enrolled in the Danish Diet, Cancer and Health cohort (*n* = 57,053). After a median follow-up of 13 years, a 45% reduction in the risk of CRC in women with higher Nordic Food Index scores (IRR _(Quintile 5_ _vs. Quintile 1)_ = 0.65, 95% CI: 0.46, 0.94. P trend 0.02) was reported, compared with women with lower scores. This trend was also observed in men, yet the association did not reach statistical significance (IRR _(Quintile 5_ _vs. Quintile 1)_ = 0.87, 95% CI: 0.61, 1.25. P trend 0.94).

Nilsson et al.’s 2013 [75] study is the only null finding for the relationship between diet quality and CRC risk. Nilsson et al. investigated the association between a LCHP score (see Table S1) and risk of CRC in 62,582 men and women from the VIP cohort. Followed for up to 17.8 years, neither men nor women with higher adherence to the LCHP eating pattern showed any increase or decrease in risk of CRC cancer incidence (men: HR _(High_ _vs. Low scores)_ = 1.00, 95% CI: 0.66, 1.52; P trend 0.511; women: HR _(High_ _vs. Low scores)_ = 0.83, 95% CI: 0.52, 1.34; P trend 0.459).

### 4.7. Gastric Cancer

One of the three studies investigating the association between diet quality and gastric cancer found a relationship between higher diet scores and lower risk of gastric cancer [35]. Using data from 485,044 men and women enrolled in EPIC from 10 different European countries, Buckland et al. (2010) [35] investigated the relationship between gastric cancer and a variation of the Mediterranean diet score (rMED, see Table S1). After a mean follow-up of 8.9 years, and an adjustment for recognised cancer risk factors, EPIC centre and age, a high rMED score was associated with a 33% reduction in gastric cancer risk (HR_(Tertile 3_ _vs. Tertile 1)_ = 0.67, 95% CI: 0.47, 0.94. P trend 0.02).

Using EPIC data (*n* = 452,269), Jeurnink et al. (2012) [49] found no relationship between DDS or any of its variations (DDSvegfr, DDSvegsub, DDSveg, or DDSfruit) and gastric cancer in men or women. Li et al. (2013) [61] reported no association between HEI-2005 or aMED scores and risk of gastric cancer incidence in 494,698 men and women enrolled in the NIH-AARP Diet and Health Study.

### 4.8. Prostate Cancer

Four studies in this review examine the relationship between diet quality and prostate cancer risk with two reporting a significant relationship between higher diet scores and lower prostate cancer risk. One of these two studies investigated a variant of the LCHP score [30] and the other [18] used the HEI-2005 and AHEI-2010 which is based on the American dietary guidelines. Nilsson et al. (2013) [75] reported no relationship between a LCHP score and prostate cancer with Kenfield et al. (2013) [55] similarly reporting null findings for a variation of the MDS.

Using data from a small Swedish cohort (*n* = 1044 men), Ax et al. (2013) [30] found a 53% reduction in prostate cancer risk in men with high adherence to a LCHP score (HR_(High_ _vs. Low scores)_ = 0.47, 95% CI: 0.21, 1.04). This association was also present when men with moderate adherence were compared to high adherers to the LCHP score (HR _(High_ _vs. Low scores)_ = 0.55, 95% CI: 0.32, 0.96). Nilsson et al. (2013) also investigate the association between a LCHP score (see Table S1) and risk of prostate cancer in 31,397 men from the VIP cohort. Followed for up to 17.8 years, men with higher adherence to the LCHP eating pattern did not show any increased or decreased risk of prostate cancer incidence (HR_(High_ _vs. Low scores)_ = 0.97, 95% CI: 0.85, 1.15; P trend 0.777). In a much larger study (*n* = 293,453 men enrolled in NIH-AARP), Bosire et al. (2013) [18] also found a significant reduction in prostate cancer risk in men with high HEI-2005 (HR _(Quintile 5_ _vs. Quintile 1)_ = 0.95, 95% CI: 0.90, 0.98) and AHEI-2010 (HR _(Quintile 5_ _vs. Quintile 1)_ = 0.96, 95% CI: 0.92, 1.00). When stratified by prostate-specific antigen (PSA) screening history, this weak association remained significant only for those men reporting a PSA screen in the preceding three years (for HEI-2005, HR _(Quintile 5_ _vs. Quintile 1)_ = 0.92, 95% CI: 0.86, 0.98, P trend 0.01; for AHEI-2010, HR _(Quintile 5_ _vs. Quintile 1)_ = 0.93, 95% CI: 0.88, 0.99, P trend 0.05). Bosire et al. [18] found no association between aMED scores and prostate cancer risk in men reporting a recent history of PSA screening (HR _(Quintile 5_ _vs. Quintile 1)_ = 0.97, 95% CI: 0.91, 1.03, P trend 0.09). Using data from 47,867 men enrolled in the HPFS, Kenfield et al. (2013) [55] investigated the association between prostate cancer incidence and a Mediterranean diet score (MMDS) as well as aMED, which differs from the traditional score by using sex-specific cut-offs for intake and eliminates the “dairy” food group (see Table S1). After a median follow-up of 23.2 years, no association between risk of prostate cancer and either the MDS (HR _(Highest_ _vs. Lowest scores)_ = 0.95, 95% CI: 0.90, 1.02, P trend 0.13) or aMED (HR _(Quintile 5_ _vs. Quintile 1)_ = 0.94, 95% CI: 0.86, 1.03, P trend 0.39) was found.

### 4.9. Head and Neck Cancer

Three of the four studies examining the relationship between head and neck cancer (HNC) risk and diet quality found associations between higher diet scores and a decreased risk of HNC. Of these three, two used the HEI-2005 [20,61], one used a score based on the Mediterranean dietary pattern [61], and the final study used the DDS, which is based on the total number of foods within the five food groups consumed, independent of quantity. No relationship was found between a variant of the LCHP dietary pattern and HNC risk [75].

Using data from the NIH-AARP study Li et al. (2014b) [20] reported a significant association between higher HEI-2005 and aMED scores and reduced HNC risk in 494,967 men and women. After 10 years of follow-up, comprising cancers of the larynx, oral cavity, and orohypopharynx, a reduced risk of HNC was found to be associated with the HEI-2005, which measures adherence to the 2005 Dietary Guidelines for Americans, in women (HR _(Quintile 5_ _vs. Quintile 1)_ = 0.48, 95% CI: 0.33, 0.70; P trend < 0.0001) and men (HR _(Quintile 5_ _vs. Quintile 1)_ = 0.74, 95% CI: 0.63, 0.89; P trend = 0.0008). This study also found significant associations between HNC incidence and aMED (see Table S1) for both men (HR _(Highest_ _vs. Lowest scores)_ = 0.80, 95% CI: 0.64, 1.01; P trend = 0.002) and women (HR_(High_ _vs. Low scores)_ = 0.42, 95% CI: 0.24, 0.74. P trend < 0.0001). In an earlier study, using data from the NIH-AARP cohort, Li et al. (2013) [61] investigate the association between incidence of oesophageal adenocarcinomas (EAC) and oesophageal squamous cell carcinomas (ESCC) with HEI-2005 scores and aMED (see Table S1). Using data from 494,968 men and women followed for an average of 9.7 years, a significant reduction in EAC and ESCC risk was associated with high HEI-2005 scores (for EAC: HR _(Quintile 5_ _vs. Quintile 1)_ = 0.75, 95% CI: 0.57, 0.98. P trend 0.01; for ESCC: HR _(Quintile 5_ _vs. Quintile 1)_ = 0.75, 95% CI: 0.57, 0.98. P trend 0.001). Similarly, this study also found a significant reduction in ESCC but not EAC risk associated with higher adherence to the aMED score (HR _(High_ _vs. Low scores)_ = 0.44, 95% CI: 0.22, 0.88. P trend 0.03). Jeurnuink et al. (2012) examined associations between diet diversity (DDS), measured as the number of different fruits and vegetables consumed across the fortnight independent of quantity, and risk of squamous cell carcinoma and adenocarcinomas of the oesophagus. After a follow-up of 8.4 years, Jeurnink et al. [49] found a decrease in risk of oesophageal squamous cell carcinoma in men and women (*n* = 452,269) with higher DDS scores (HR _(Tertile 3_ _vs. Tertile 1)_ = 0.42, 95% CI: 0.17, 1.04. P trend 0.07). This association was particularly significant for the sub-score of the DDS which measured diversity of fruit intake across the fortnight (DDSfr) (HR _(Tertile 3_ _vs. Tertile 1)_ = 0.48, 95% CI: 0.21, 1.11. P trend 0.04). Jeurnink et al. found no association between the risk of adenocarcinomas of the oesophagus and diet diversity. Nilsson et al. (2013) [75] investigated the association between a LCHP score (see Table S1) and risk of respiratory tract cancer in 62,582 men and women from the VIP cohort. Followed for up to 17.8 years, neither men nor women with higher adherence to the LCHP eating pattern showed any increase or decrease in risk of respiratory tract cancer incidence (men: HR _(High_ _vs. Low scores)_ = 1.24, 95% CI: 0.62, 2.47; P trend 0.381); women: HR _(High_ _vs. Low scores)_ = 1.37, 95% CI: 0.67, 2.82; P trend 0.328).

### 4.10. Risk of Other Cancers

Using NIH-AARP data, another study from Li et al. (2014a) [62] investigated the association between hepatocellular carcinoma (HCC) and adherence to the HEI-2005 and aMED scores. After an 11 year follow-up, a 28% and 38% reduced risk of hepatocellular carcinoma in those with high HEI-2005 (HR _(Quintile 5_ _vs. Quintile 1)_ = 0.72, 95% CI: 0.53, 0.97; P trend = 0.03) and aMED (HR = 0.62, 95% CI: 0.47, 0.84; P trend = 0.0002) scores was found. Whilst finding no association between dietary diversity scores and lung cancer among 452,187 men and women enrolled in EPIC, Buchner et al. (2010) [34] found a 27% reduced risk of lung cancer in current smokers with high DDSveggr scores (HR _(Quartile 4_ _vs. Quartile 1)_ = 0.73, 95% CI: 0.57, 0.93). Using EPIC data in another study, Buchner et al. (2011) [33] found no protective benefit of any DDS component (DDSvegfr; DDSveggr; DDSvepr; DDSfr) and bladder cancer risk. Buchner et al. (2011) [33], however, did report a slightly increased risk of risk of bladder cancer for DDSvegfr, mostly among never-smokers (HR _(Tertile 3_ _vs. Tertile 1)_ = 1.72, 95% CI: 1.00, 2.97. P trend 0.05) and particularly among men (HR _(Tertile 3_ _vs. Tertile 1)_ = 2.22, 95% CI: 0.88, 5.57). Conversely, higher DDSfrveg scores in women were linked to reduced bladder cancer risk (HR _(Tertile 3_ _vs. Tertile 1)_ = 0.74, 95% CI: 0.49, 1.11. P trend 0.12) particularly among ever smokers (HR _(Tertile 3_ _vs. Tertile 1)_ = 0.55, 95% CI: 0.32, 0.97. P trend 0.03). Buckland et al. (2014) [37] examined the association between risk of bladder cancer and diet quality, measured using rMED (see Table S1), in 477,312 men and women enrolled in EPIC. A non-significant association between rMED scores and risk of bladder cancer (HR _(High_ _vs. Low scores)_ = 0.84, 95% CI: 0.69, 1.03. P trend 0.107) was found. After stratification by smoking status, the non-significant association became significant with a 34% reduction in bladder cancer risk observed among current smokers with high aMED scores (HR_(High_ _vs. Low scores)_ = 0.66, 95% CI: 0.47, 0.93. P trend 0.043). Using scores to represent dietary diversity (DDSvegfr, DDSveggr, DDSvegsub, DDSfr), Jeurnink et al. (2012) [49] found no association between any DDS variation and risk of adenocarcinoma of the gastro-oesophageal junction (GEJ), cardia and non-cardia, in men and women enrolled in EPIC (*n* = 452,269). Using NIH-AARP data from 537,218 men and women, Arem et al. (2013) [29] reported a relationship between higher HEI-2005 scores and lower pancreatic cancer risk (HR_(Q1__vs. Q2) =_ 0.85; 95% CI (0.74, 0.97)).

### 4.11. Diet Quality and Cancer Mortality

Studies examining the relationship between diet quality and cancer mortality (*n* = 31) are shown in Supplementary Table S3.

### 4.12. Diet Quality and Risk of Mortality from All-Sites Cancer

Just under 47% of studies investigating the relationship between diet quality and all-sites cancer mortality risk reported associations between higher diet scores and lower risk of mortality (*n* = 13). A greater proportion found no association (*n* = 16), and two studies found an increased risk of cancer mortality associated with diet quality as measured using an adaptation of the LCHP score [12,46].

### 4.13. Mediterranean Diet Scores

A number of variations of the MDS (MDS; tMED; aMED; mMDS; MMDS—see Table S1) were found to be associated with reduced risk of cancer mortality in cohort studies (*n* = 5) [70,76,78,79,82]. A strong association was found by Mitrou et al. (2007) [70] between Fung et al.’s (2005) original aMED and Mitrou et al.’s tMED scores and overall cancer mortality risk using data from adult men and women enrolled in NIH-AARP (*n* = 214,284) and followed for ten years. Mitrou et al.’s (2007) [70] study found an association between higher aMED (HR_(High_ _vs. Low scores)_ = 0.83, 95% CI: 0.76, 0.91; P trend < 0.001) and tMED (HR_(High_ _vs. Low scores)_ = 0.79, 95% CI: 0.72, 0.87) scores and reduced risk of mortality from any cancer in 214,284 men with no history of chronic disease. A similar relationship with all-sites cancer mortality was observed in 166,012 women for the aMED (HR_(High_ _vs. Low scores)_ = 0.88, 95% CI: 0.78, 1.00; P trend 0.04) but not tMED scores (HR_(High_ _vs. Low scores)_ = 0.89, 95% CI: 0.79, 1.01). Further evidence in support of Mediterranean dietary patterns was found in a more recent NIH-AARP study from Reedy et al. (2014) [76] (*n* = 492,623 men and women aged 50–71 years at baseline in 1995–6). Adapting the MDS for the American context aMED, a significant 20% decrease in cancer mortality risk among those in the highest compared to the lowest quintile of aMED scores (HR_(Quintile 5_ _vs. Quintile 1)_ = 0.80, 95% CI: 0.77, 0.84) was found. Tognon et al. (2012) [78] similarly reported a significant reduction in cancer mortality in men enrolled in the Vasterbotten Intervention Project (VIP) (*n* = 37,546) with higher mMDS scores (HR_(continuous)_ = 0.92, 95% CI: 0.87, 0.98. P trend < 0.01). This relationship was not observed in women enrolled in the VIP (*n* = 39,605) (HR_(continuous)_ = 0.98, 95% CI: 0.92, 1.03. P trend > 0.05). Further, using MMDS scores, Trichopoulou et al. (2003) [79] found a significantly reduced risk of overall cancer mortality in men and women enrolled in EPIC (*n* = 22,043) and followed for eight years (HR_(per two point increment)_ = 0.76, 95% CI: 0.59, 0.98). Finally, Vormund et al. (2014) [82] investigated the association between MDS scores and cancer mortality in a population-based Swedish cohort of adult men and women (*n* = 17,861). After a mean follow-up of 21.1 years, a significantly reduced risk of cancer mortality in the pooled estimate from men and women with high MDS scores (HR_(High_ _vs. Low scores) =_ 0.83, 95% CI: 0.70, 0.97) was found. After stratification by sex, the relationship remained significant for men (HR_(High_ _vs. Low scores) =_ 0.80, 95% CI: 0.65, 0.99) but not for women (HR_(High_ _vs. Low scores) =_ 0.92, 95% CI: 0.73, 1.17).

Six additional studies found no association between MDS variations (MDS, rMED, and MMDS) and overall cancer mortality risk [36,42,55,56,58,64]. Using data from the Spanish segment of EPIC (*n* = 40,622 men and women), Buckland et al. (2011) found no association between rMED scores and cancer mortality (HR_(Quintile 3_ _vs. Quintile 1)_ = 0.92, 95% CI: 0.75, 1.12. P trend 0.414). Similarly, Cuenca-Garcia et al. reported no association between the MDS and overall cancer mortality risk (HR_(Quartile 4_ _vs. Quartile 1)_ = 1.63, 95% CI: 0.91, 2.92. P trend 0.432) in men and women enrolled in the American Aerobics Centre Longitudinal Study (*n* = 12,499) and followed for 11 years. Using data from 47,867 men enrolled in the HPFS, Kenfield et al. (2013) [55] investigated the association between prostate cancer mortality and MMDS as well as aMED. After a median follow-up of 23.2 years, no association between mortality from prostate cancer and either the MDS (HR_(High_ _vs. Low scores)_ = 1.01, 95% CI: 0.75, 1.38, P trend 0.95) or aMED (HR_(Quintile 5_ _vs. Quintile 1)_ = 1.14, 95% CI: 0.73, 1.76, P trend 0.83) was found. Using data from the Spanish segment of EPIC, Knoops et al. (2004) [56] also investigated the association between cancer mortality and the Mediterranean diet pattern as measured by a variation of the MMDS where alcohol was excluded (see Table S1). After a 10-year follow-up of 2339 Spanish men and women aged 70–90 years at baseline in 1988, no association between higher MMDS scores and cancer mortality (RR_(≥4 points on MMDS V <4 points)_ = 0.90, 95% CI: 0.70, 1.17) was found. Investigating the association between the MMDS and cancer mortality in younger women, Lagiou et al. (2006) [58] found a significant reduction in cancer mortality among women aged ≥40 years in the age-adjusted-only Cox proportion hazards regression model (HR_(Tertile 3_ _vs. Tertile 1)_ = 0.67, 95% CI: 0.48, 0.93, *p* < 0.05). This association, however, was not significant for younger women (<40 years of age) (HR_(Tertile 3_ _vs. Tertile 1)_ = 1.07, 95% CI: 0.79, 1.43) and became non-significant in the fully adjusted model (HR_(Tertile 3_ _vs. Tertile 1)_ = 0·80, 95% CI: 0·57, 1·13), *p* > 0.05). Reporting findings from the Spanish Seguimiento Universidad de Navarra (SUN) project (*n* = 15,535), Martinez-Gonzalez et al. (2012) [64] found no association between diet quality, as measured by the MMDS, and cancer mortality in this young cohort (of men and women μ age 38 ± 12 years) (HR_(per two unit increase in MMDS)_ = 1.03 (95% CI: 0.73, 1.45. P trend 0.80).

### 4.14. Healthy Eating Index

A number of studies (*n* = 3) investigating repatriations of the HEI including the HEI-2010, AHEI, and AHEI-2010 were also found to be associated with reduced risk of cancer mortality [71,76,81]. Mursu et al. (2013) [71] found evidence of an association between higher AHEI scores and an a priori diet quality score and reduced overall cancer mortality within the Iowa Women’s Health Study (*n* = 29,634). After a mean follow-up of 20.3 years, women with high AHEI scores had a 12% reduction in cancer mortality (RR_(Quartile 4_ _vs. Quartile 1)_ = 0.88, 95% CI: 0.79, 0.98. P trend < 0.001). Using NIH-AARP data from 214,284 adult men and women, Reedy et al. (2013) found significant reductions of 24% and 18% for associations between the HEI-2010 and AHEI-2010 and cancer mortality risk, respectively (for HEI-2010: HR_(Quintile 5_ _vs. Quintile 1)_ = 0.76, 95% CI: 0.72, 0.80; for AHEI-2010: HR_(Quintile 5_ _vs. Quintile 1)_ = 0.82, 95% CI: 0.78, 0.86). Reporting data from 77,782 nurses enrolled in the NHS, van Dam et al. (2008) [81] examined the association between all-sites cancer mortality and the AHEI, which is based on McCullough et al.’s (2002) [67] original score with the exclusion of nutritional supplements. After a 24-year follow-up, a significantly reduced risk of cancer mortality in women in the highest quintile compared with the lowest quintile of AHEI scores was found (RR_(Quintile 5_ _vs. Quintile 1)__=_ 0.72, 95% CI: 0.65, 0.79. P trend < 0.05).

Two additional studies found no association between higher scores of the AHEI [28] and the original HEI [54] and overall cancer mortality risk in two large population-based cohorts. Using British Whitehall II cohort data (*n* = 7319 men and women), Akbarly et al. (2011) reported no association between AHEI scores and mortality from cancer (HR_(Tertile 3_ _vs. Tertile 1)_: 0.80, 95% CI: 0.58, 1.11) after an average follow-up of 18 years. Calculated from 24-h recall data from 17,611 men and women enrolled NHANES III, Kappeler et al. (2013) [54] found no association between higher HEI scores and cancer mortality risk after a 22-year follow-up (HR_(Tertile 3_ _vs. Tertile 1)_ = 0.75, 95% CI: 0.51, 1.11, P trend 0.14).

### 4.15. The Recommended Food Score

The RFS was found to be linked to reduced all-sites cancer mortality in three studies [52,63,68]. An early study from Kant et al. (2000) [52] examined the association between all-sites cancer mortality and the Recommended Food Score, which measures diet quality as adherence to the 1995 Dietary Guidelines for Americans. Using data from phase II of the BCDDP (*n* = 42,254) and after a follow-up of 5.6 years, it was reported that women with higher quality diets had a 40% reduction in mortality from all-sites cancer when compared to lower quality diets (RR_(Quartile 4_ _vs. Quartile 1)_ = 0.60, 95% CI: 0.49, 0.74. P trend < 0.001). Using Kant et al.’s RFS with more recent BCDDP phases III and IV data (*n* = 42,234), Mai et al. (2005) [63] found a significant reduction in overall cancer mortality amongst women with higher diet quality (HR_(Quartile 4_ _vs. Quartile 1)_ = 0.74, 95% CI: 0.63, 0.86; P trend < 0.001). After a 10-year follow-up, Michels and Wolk (2002) [68] found a 24% reduction in cancer mortality risk in women enrolled in the Swedish Mammography Screening Project (*n* = 59,038) (HR_(Quintile 5_ _vs. Quintile 1)_ = 0.76, 95% CI: 0.60, 0.96. P trend 0.005). Michels and Wolk also investigated the relationship between cancer mortality and a non-RFS, which measured the intake of foods associated with increased risk of chronic disease. Women with higher non-RFS scores demonstrated a 48% increased risk of cancer mortality in the fully adjusted model (HR_(Quintile 5_ _vs. Quintile 1)_ = 1.52, 95% CI: 1.13, 2.05. P trend 0.02).

Contrasting Michels and Wolk (2002), and using data from the Cohort of Swedish Men (*n* = 40,837), Kaluza et al. (2009) [50] found no association between risk of all-sites cancer and the RFS, (HR _(High_ _vs. Low scores)_ = 1.09, 95% CI: 0.84, 1.41. P trend 0.28). Kaluza further found no association with the non-RFS and risk of cancer in men after an average follow-up of almost 8 years (HR _(High_ _vs. Low scores)_ = 1.17, 95% CI: 0.94, 1.46. P trend 0.49).

### 4.16. Diet Diversity Scores

Reporting data from the small Taiwanese Elderly Nutrition and Health Survey (*n* = 1743 men and women aged over 65 years), Lee et al. (2011) [60] found an association between higher diet diversity scores (DDS) and lower risk of cancer mortality (HR = 0.46, 95% CI: 0.20, 1.07, P trend 0.03). However, an earlier NHANES I analysis (*n* = 10,337) found no association between DDS scores and overall cancer mortality in men (RR_(scores 0–2 Vs. 5)_ = 1.3, 95% CI: 0.8, 2.1) or women (RR_(scores 0–2 Vs. 5)_ = 1.4, 95% CI: 0.8, 2.3) after a median follow-up of 14.2 years (Kant et al., 1995) [51].

### 4.17. Other Diet Quality Scores Associated with Cancer Mortality Risk

Other scores found to be associated with reduced overall cancer mortality risk include the (1) DBS [53], which measures six equally weighted components based on recommendations from the 2005 Dietary Guidelines for Americans; (2) the ODI-R, which measures adherence to the Taiwanese dietary guidelines [60]; (3) a DASH score [76], which measures adherence to the DASH dietary pattern; (4) the Aussie DQI, which is based on the 2003 Australian Dietary Guidelines [84]; (5) the DQI-SNR, which is based on the 2005 Swedish Nutrition Recommendations [43]; (6) the a priori diet quality score, which is based on recommendations for prevention of chronic disease [71]; and (7) the HDI, which is based on the WHO recommendations for the prevention of chronic disease [47].

Using NIH-AARP data (*n* = 350,886), Kant et al. (2009) found a significant reduction in cancer mortality amongst men and women with higher DBS scores after a 10-year follow-up (men: RR_(Quintile 5_ _vs. Quintile 1)_ = 0.79 95% CI: 0.73, 0.86. *p* < 0.0001); and women: RR_(Quintile 5_ _vs. Quintile 1)_ = 0.81 95% CI: 0.73, 0.90. *p* < 0.0001). Using data from a small cohort of older Taiwanese men and women, Lee et al. (2010) further reported a non-significant association between higher diet quality measured using the ODI-R and lower overall cancer mortality (HR = 0.48, 95% CI: 0.25, 0.93, P trend 0.14). Using NIH-AARP data (*n* = 492,623 men and women), Reedy et al. (2014) [76] found a significant 20% reduction in participants with high DASH scores (HR _(Quintile 5_ _vs. Quintile 1)_ = 0.80, 95% CI: 0.76, 0.84). Zarrin et al.’s [84] small study (*n* = 1355 men and women enrolled in the Nambour Skin Cancer Study) also found an association between higher Aussie-DQI scores and reduced cancer mortality in men (HR _(Tertile 3_ _vs. Tertile 1)_ = 0.30, 95% CI: 0.11, 0.83; P trend 0.06) and women (HR _(Tertile 3_ _vs. Tertile 1)_ = 0.64, 95% CI: 0.24, 1.68; P trend 0.65). However, whilst the HR for men almost reaches significance at the 0.05 level, neither of the associations for men or women is statistically significant. Drake et al. [43] reported no association between diet quality and cancer mortality in women for any variation of the DQI-SNR but found an association for men using Model 1, which uses scoring based on the 2005 Swedish Nutrition Recommendation cut-offs rather than cohort medians or quintiles (HR_(Tertile 3_ _vs. Tertile 1)_ = 0.82 (95% CI: 0.68, 0.97). Despite findings of this inverse association in Swedish men, there was no significant linear trend (P trend 0.61). After a mean follow-up of 29,634 women for 20.3 years, Mursu et al. found that women with high a priori diet quality scores, calculated from FFQ data collected at baseline in 1986, had a 14% reduction in cancer mortality (RR_(Quartile 4_ _vs. Quartile 1)_ = 0.86, 95% CI: 0.77, 0.95. P trend 0.025). Using additional FFQ data collected during a 2004 follow-up of the same cohort (*n* = 15,076), followed until censoring on 31 December 2008, Mursu et al. found that those women with higher a priori (RR_(Quartile 4_ _vs. Quartile 1)_ = 0.70, 95% CI: 0.52, 0.94. P trend 0.028) scores had a significantly reduced risk of cancer mortality. Combining cohorts from three countries involved in the Seven Countries Study (Italy, The Netherlands, and Finland; *N*_total_ = 3045), Huijbregts et al. (1997) [47] also found a non-significant 15% reduction in all-sites cancer risk among men aged 50–70 years with higher HDI scores (P trend 0.13).

### 4.18. Diet Quality Scores Associated with Increased Cancer Mortality Risk

Contrasting most findings of this review, Fung et al. (2011) [12] reported an increased risk of cancer mortality in women enrolled in the NHS (*n* = 85,168) with higher scores of a LCHP index (HR_(Decile 10 V Decile 1)_ = 1.19, 95% CI: 0.99, 1.42. P trend 0.128). This analysis was repeated again by Fung et al. (2010) [46] for two variations of the LCHP index, one including only protein from animal sources (LCHP-AB) and the other from vegetable sources (LCHP-V). Women with higher LCHP-AB scores showed an increased risk of death from all-sites cancer (HR_(Decile 10 V Decile 1)_ = 1.28 (95% CI: 1.02, 1.60. P trend 0.089), whereas no association was found for the LCHP-V index (HR_(Decile 10 V Decile 1)_ = 0.96, 95% CI: 0.87, 1.05. P trend 0.23) (Fung et al., 2010) [46] repeated the aforementioned comparisons in men from the HPFS, aged 40–79 years at baseline in 1986 and followed for up to 20 years. Fung et al. (2010) found an increased risk of all-sites cancer in men with higher LCHP scores (HR_(Decile 10 V Decile 1)_ = 1.32, 95% CI: 1.11, 1.57. P trend < 0.001) and in the LCHP-AB score in particular (HR_(Decile 10 V Decile 1)_ = 1.45, 95% CI: 1.23, 1.72. P trend < 0.001). No associations observed in men for the LCHP-V variant (Fung et al., 2010). However, in an additional study, using data from the Scandinavian Women’s Lifestyle and Health Cohort (*n* = 42,237 women), Lagiou et al. (2007) [59] reported no association between LCHP scores and cancer mortality risk (HR_(per 2 unit increase in LCHP score)_ = 1.02, 95% CI: 0.96, 1.08).

### 4.19. Other Diet Quality Scores Not Associated with Cancer Mortality Risk

Additional diet quality scores found not to be associated with cancer mortality risk include (1) the IDI [42]; which measures (2) the DQI [42,77]; (3) a LCHP score [59]; (4) a reduced-salt healthy Japanese Diet Score [72]; (5) and a traditional Sami Diet Score, which is characterised by low intake of vegetables, bread and fibre as well as moderate intake of red meat, fatty fish, total fat, berries, and boiled coffee [73].

Cuenca-Garcia et al. (2014) [42] reported findings from the American Aerobics Centre Longitudinal Study and data for 12,499 men and women with a mean follow-up of 11 years. Cuenca-Garcia et al., in one of the few studies using a three-day food record method of dietary assessment, reported null findings for associations between high scores of the IDI (HR_(Quartile 4_ _vs. Quartile 1)_ = 1.06, 95% CI: 0.61, 1.86. P trend 0.913) or DQI (HR_(Q4Vs.Q1)_ = 1.26, 95% CI: 0.72, 2.22. P trend 0.458) and risk of cancer mortality. Seymour et al. (2003) [77], in an older study, reported data from the American Cancer Society’s Cancer Prevention Study II Nutrition Cohort (*n* = 115,833). After a four-year follow-up, Seymour et al. found no association between DQI scores and cancer mortality for both men (RR_(Quintile 5_ _vs. Quintile 1)_ = 0.92, 95% CI: 0.63, 1.34; P trend 0.28) and women (RR_(Quintile 5_ _vs. Quintile 1)_ = 0.61, 95% CI: 0.32, 1.18; P trend 0.28). Nilsson et al. (2012a) and (2012b) [73,74] also reported data from the same group of men and women from the VIP (*n* = 77,319). Followed for up to 19 years, Nilsson et al. (2012a) [74] found no association between higher LCHP scores and cancer mortality in men enrolled in VIP (HR _(continuous)_ = 1.00, 95% CI: 0.98, 1.03. P trend 0.851) or women (HR _(continuous)_ = 1.00, 95% CI: 0.97, 1.02. P trend 0.878). Following the VIP cohort for 19 years, Nilsson et al. (2012b) [73] also found no increase or decrease in cancer risk when comparing men (HR_(continuous)_1.05, 95% CI: 0.99, 1.10; P trend 0.102) or women (HR_(continuous)_ = 1.03, 95% CI: 0.97, 1.09; P trend 0.304) with high Traditional Sami Diet scores to those with low Traditional Sami Diet scores. Nakumura et al. (2009) [72], in the only study using Japanese longitudinal cohort data (*n* = 9086), also found no association between a reduced-salt Japanese diet score and cancer mortality (HR_(Tertile 3_ _vs. Tertile 1)_ = 0.85, 95% CI: 0.69, 1.05; P trend 0.13).

### 4.20. Diet Quality and Risk of Site-Specific Cancer Mortality

Four studies investigated the relationship between site-specific cancer mortality and diet quality [46,55,63,78]. Using data from the American BCDDP, Mai et al. (2005) [63] found a significant reduction in mortality from lung (HR_(Quartile 4_ _vs. Quartile 1)_ = 0.54 (95% CI: 0.29, 0.84; P trend < 0.001) and CRC (HR_(Quartile 4_ _vs. Quartile 1)_ = 0.49, 95% CI: 0.29, 0.86; P trend < 0.01) among women (μ age of 61 years) in the upper quartile of RFS scores compared with those in the lower quartile. Mai et al. also reported a borderline significant result for breast cancer mortality (HR_(Quartile 4_ _vs. Quartile 1)_ = 0.75, 95% CI: 0.56, 1.00; P trend 0.06). Tognon et al. (2012) [78] investigated the association between the mMDS and site-specific cancer mortality among Swedish men and women enrolled in the VIP cohort (*n* = 77,151). After a follow-up period of up to 19 years, Tognon et al. reported no association between mMDS scores and pancreatic, colorectal, stomach, or breast cancer in women (*n* = 38,034). In men (*n* = 39,950), Tognon et al. did find an association between higher mMDS scores and pancreatic cancer (for every one unit increase in mMDS score, HR = 0.82, 95% CI: 0.68, 0.99) but not for colorectal, stomach, or prostate cancer. Using data from the HPFS (*n* = 47,867 men aged 40–75 years), Kenfield et al. (2014) [55] reported exclusively on the relationship between mortality from prostate cancer and diet quality as measured by the MMDS. After a 24-year follow-up (median 23.2 years), Kenfield et al. reported 1181 deaths attributed to prostate cancer through data linkage with the National Death Index. No association was observed between higher adherence to the MMDS and prostate cancer death (HR _(Quintile 5_ _vs. Quintile 1)_ = 1.14, 95% CI: 0.73, 1.76, P trend 0.83). Using HPFS data pooled with data from women enrolled in the NHS, Fung et al. (2010) [46] found a significantly increased risk of CRC death in men and women with higher adherence to a (LCHP-AB) index (HR _(Quintile 5_ _vs. Quintile 1)_ = 1.31, 95% CI: 1.06, 1.62. P trend 0.048) but found no such association with CRC death for the total LCHP index or a variation of the LCHP index which was based on vegetable protein only (LCHP-V) (total LCHP index: HR _(Quintile 5_ _vs. Quintile 1)_ = 1.13, 95% CI: 0.92, 1.40. P trend 0.21; LCHP-V index: HR_(Quintile 5_ _vs. Quintile 1)_ = 0.96, 95% CI: 0.78, 1.17. P trend 0.074). Similarly, higher adherence of the pooled cohort (*n* = 129,716, men and women) to the LCHP and LCHP-AB index further showed an increased risk of lung cancer mortality (total LCHP index: HR _(Quintile 5_ _vs. Quintile 1)_ = 1.22, 95% CI: 1.05, 1.42. P trend 0.003; LCHP-AB index: HR _(Quintile 5_ _vs. Quintile 1_ = 1.23, 95% CI: 1.03, 1.46. P trend 0.011). Fung et al. (2010) [46] also found no association between higher adherence to the total LCHP, LCHP-AB, or LCHP-V scores and risk of mortality from prostate or breast cancer.

## 5. Discussion

This systematic review provides a comprehensive summary of studies examining the relationship between diet quality, as evaluated by a priori diet quality indices or scores and cancer outcomes, including total and specific cancer risk and mortality. This body of evidence suggests that higher diet quality, as measured by a number of indices, is associated with reduced risk of postmenopausal breast cancer, CRC, and HNC. All-sites cancer risk and cancer mortality were not consistently associated with any of the diet quality scores using any of the indices.

The most common cohort datasets evaluated in studies included in this review were the EPIC, NIH-AARP, NHS, HPFS, and VIP, which are predominantly population cohorts from the United States, but other countries include France, the United Kingdom, and Sweden. Each of these countries differs in the all-cause cancer rate with cohorts including the country ranked number one down to the country ranked 34th in cancer rate [87]. Dietary patterns also differ between these countries, and there are other factors that contribute to country-specific cancer incidence and mortality [88]. Therefore, the general conclusions from this review must be interpreted with caution when applying findings to other populations.

Food frequency questionnaires (FFQs) were the most frequently used dietary assessment tool used to inform diet indices that measured overall diet quality. FFQs are the most practical and cost-effective method for assessing dietary intake in large cohorts, but may have some limitations in this context. The reference periods for FFQs can vary, and, while shorter reference periods may capture usual dietary intake for that period, they may not reflect longer-term dietary intake, which would have a greater impact on chronic disease risk and mortality.

There were 55 different diet quality indices identified in this review, which included the original indices plus variations of these indices such as the HEI, MDS, RFS, DASH, and DQI. A number of diet quality indices were originally developed for assessing relationships between diet quality and other chronic diseases, such as the DASH index, which is based on heart health guidelines. However, a number of studies use this score to examine potential relationships with cancer incidence or mortality. Other diet quality indices were variations of already-developed indices that had been specifically developed to investigate the relationship between diet quality and cancer. Buckland et al. (2013) [17] excluded the alcohol component from the MDS score when examining the relationship between the Mediterranean diet score and breast cancer risk, as alcohol is a known factor associated with greater breast cancer incidence. As more evidence emerges on associations between dietary patterns and cancer risk and mortality, further effort should be placed on developing specific tools for assessing associations between diet quality and cancer.

The National Cancer Institute projects that some of the most common cancers in the future will be breast cancer, colon and rectum cancer, prostate cancer, lung cancer, and pancreatic cancer [89]. Within this review, the types of cancer most frequently investigated were breast cancer, CRC, prostate cancer, and HNC. However, lung cancer and pancreatic cancer have received little attention. This suggests that there needs to be some shift in priority, as some prevalent cancers receive more focus than others. In this review, of these priority cancers, diet quality was favourably associated with breast, colorectal, and HNC. Of note, the strongest associations with breast cancer were those in postmenopausal women with oestrogen receptor (ER) negative tumours [12,17,45,80], whereas these associations did not exist for ER+ or premenopausal women. It has previously been reported that a diet lower in fat (20% vs.. 30% of total energy) may be associated with reduced risk of breast cancer in ER– females [90]. The diet quality indices in this review that were associated with a reduced risk of breast cancer were the MDS, HEI, DASH, and RFS, or variations thereof, and, although these indices do not typically quantify total fat intake, they do score the type of fat, particularly saturated fat [91]. Higher intakes of saturated fats have been linked to an increased risk of breast cancer [92]. Therefore, a diet higher in total fat may also be higher in saturated fat, explaining the link between a low-fat diet and reduced cancer risk.

There were also convincing associations between diet quality and CRC; however, there was inconsistency in whether these associations existed in men [13,69] or women [10,11,57,69] when stratified by sex. Higher diet quality scores were also associated with reduced risk of HNC. Higher scores in variations of the MDS, HEI, DASH, and RFS were repeatedly found to be associated with reduced colorectal and HNC risk.

There were few studies that found that a higher diet quality lowered the risk of overall cancer risk, with variability in findings from studies using the same diet quality indices. For example, the study that found an association between a higher AHEI and reduced cancer risk used a version of the AHEI based on the 2010 dietary guidelines [39], whereas one of the studies that found no association used an older version of the AHEI that was based on dietary guidelines prior to this time [67]. The frequency of updates of the dietary guidelines from which many of the indices are based may contribute to variations in findings. As evidence for the associations between diet and chronic disease has evolved, so too have dietary guidelines, with more recent guidelines emphasising the healthiest choices within each food group, such as higher intakes of whole grains and fish and lower intakes of processed meats [39]. Therefore, these updated diet quality indices, compared with older versions, may have stronger associations with cancer risk when applied to older studies. Future evaluation could re-examine some of the previously published evaluations using these updated diet quality indices.

Less than half of the studies investigating the relationship between diet quality and cancer mortality found an association between higher diet quality and reduced cancer mortality. However, of those that did, the MDS, HEI, RFS, and DASH indices, or variations thereof, were the diet quality scores showing the strongest associations with cancer mortality risk. The features of these diet quality scores associated with lower risk included higher intakes of total or non-starchy vegetables, legumes, whole grains, and fruits, including nuts, with moderate to high intakes of dairy, moderate intakes of poultry-, seafood-, and plant-based proteins, and a low intake of red meats, alcohol, and sugar-sweetened products. The more specific the index was (e.g., non-starchy or green and orange vegetables rather than assessing total vegetables, seafood and plant protein rather than total meat, plus the inclusion of empty calories from products with added sugar), the stronger the association with cancer risk and mortality was. Diet quality indices that were less frequently associated with cancer risk were less specific and tended to be older versions of the indices.

A number of studies have also reported a positive association between a higher LCHP score and increased risk of mortality from cancer [12,46]. However, when the protein was divided into protein from animal sources versus plant sources, only animal fat was associated with greater risk of mortality from cancer. A study published in 2014 reported that consuming a diet with moderate-to-high protein content was associated with a three- to four-fold increased risk of cancer mortality compared to a low protein diet [93]. Moreover, when animal-derived protein sources were excluded, the risk of mortality was significantly reduced. This may also explain the fewer than expected associations between higher diet quality scores and lower cancer mortality. Again, as diet quality indices have evolved, food groups have become more specific, including protein from animal and plant sources, total grains and whole grains, and total vegetables—non-starchy and coloured vegetables—which has likely had an impact on the variability in the reported results. This highlights an important consideration when using diet indices to assess diet quality when there are subscales that include particular food groups that may contain both anti- and pro-carcinogenic properties, such as the meat group.

This review has limitations that need to be acknowledged. These include a high amount of heterogeneity between studies, particularly with the study-specific diet quality index used and the cohorts studied. Additionally, there may be some overlap in data from different studies conducted within the same region or when results are reported for a complete dataset and a sub-group of that dataset. When each of these studies reports positive findings, there is a possibility that the results are overstating the actual findings. In these situations, results should be treated with caution. However, strengths include the extensive body of literature reviewed and adherence to the PRISMA guidelines for reporting of systematic reviews.

This systematic review highlights that the current evidence examining associations between diet quality and cancer risk indicates that higher scores using a number of diet indices confer reduced risk, particularly for breast, colorectal, and HNC. However, there is still inconsistency in findings from studies investigating the association between diet quality and all-cause cancer risk and mortality, as well as some specific types of cancer. In this review, there were a wide variety of diet quality indices as well as differing versions that were investigated, which likely contributed to some of the inconsistency in findings. As further research is conducted, dietary guidelines will be updated and many of the diet quality indices that are based on dietary guidelines will be refined. Therefore, additional research into the relationship between diet quality and cancer risk and mortality, particularly using the most recent diet quality indices, is warranted.

## 6. Conclusions and Recommendations

Higher diet quality scores are associated with reduced site-specific but not all-cause cancer risk. Evidence is less conclusive for cancer mortality and suggests additional factors may influence cancer survival. The development of a validated cancer-specific diet quality score could benefit future prospective epidemiological studies as well as public health and policy arenas. Positive lifestyle change may favourably influence the development of cancer. The challenge for public health is to educate the population about components of a cancer-preventing diet and the small changes in eating habits that can lower cancer risk and improve cancer outcomes.

## Figures and Tables

**Table 1:** Inclusion and exclusion eligibility criteria.

Inclusion Criteria	Exclusion Criteria
Studies reporting on a predefined diet quality score and cancer risk ^1^	Studies using factor analysis or clustering methods for dietary assessment
Studies reporting on a predefined diet quality score and cancer mortality ^1^	Melanoma
Studies conducted in high income countries ^2^ Studies published in the English language	Studies published in a language other than English
Adult populations	Studies in animals
Prevalence or cross-sectional study design & cohort studies	Health conditions that might influence diet quality (e.g., diabetes)

^1^ Total cancer and cancers of the colon, rectum, breast, stomach, prostate, head and neck; ^2^ Based on OECD criteria; countries include Australia, Austria, Belgium, Canada, Czech Republic, Denmark, Finland, France, Germany, Greece, Hungary, Iceland, Ireland, Italy, Japan, Korea, Luxembourg, the Netherlands, New Zealand, Norway, Portugal, Slovak Republic, Spain, Sweden, Switzerland, the United Kingdom, and the United States of America.

**Table 2:** Summary of included studies.

Title	Study	Location	Cohort	Diet Quality Score
1	Akbaraly TN, et al. (2011) [28]	UK	Whitehall II cohort	AHEI
2	Arem H., et al., (2013) [29]	USA	NIH-AARP study	HEI-2005
3	Ax E., et al., (2013) [30]	Sweden	ULSAM	MMDS; LCHP Score
4	Bamia C, et al., (2013) [10]	Northern and Southern Europe	EPIC	MMDS; CSMMDS
5	Berentzen NE., et al., (2013) [31]	Netherlands	EPIC-NL	HDI
6	Benetou V., et al., (2008) [32]	Greece	EPIC	MMDS
7	Bosire C., et al., (2013) [18]	USA	NIH-AARP	HEI-2005; aMED; AHEI-2010
8	Buchner FL, et al., (2011) [33]	Northern and Southern Europe	EPIC	DDS
9	Buchner FL., et al., (2010) [34]	Northern and Southern Europe	EPIC	DDS
10	Buckland G., et al., (2010) [35]	Northern and Southern Europe	EPIC	rMED
11	Buckland G., et al., (2011) [36]	Spain	EPIC—Spain	rMED
12	Buckland G., et al., (2013) [17]	Northern and Southern Europe	EPIC	arMED
13	Buckland G., et al., (2014) [37]	Northern and Southern Europe	EPIC	rMED
14	Cade JE, et al., (2011) [38]	UK	UKWCS	MMDS; HDI
15	Chiuve SE, et al., (2012) [39]	USA	NHS HPFS	HEI; AHEI-2010
16	Couto E., et al., (2011) [40]	Northern and Southern Europe	EPIC	MMDS
17	Couto E., et al., (2013) [41]	Sweden	Swedish WLH cohort	MMDS
18	Cuenca-Garcia M., et al., (2014) [42]	USA	ACLS	Ideal Diet Index (IDI); MMDS; DQI
19	Drake I., et al., (2012) [43]	Sweden	Malmö Diet and Cancer cohort	DQI-SNR
20	Fitzgerald AL, et al., (2002) [44]	Canada	Nova Scotia Nutrition Survey	Diet quality score based on the Nova Scotia DRIs
21	Fung TT, et al., (2006) [45]	USA	NHS	HEI-f; AHEI; DQIR; RFS; aMED
22	Fung TT., et al., (2010) [11]	USA	NHS HPFS	aMED; DASH score
23	Fung TT., et al., (2010) [46]	USA	NHS HPFS	LCHP
24	Fung TT., et al., (2011) [12]	USA	NHS	LCHP score; DASH score
25	Huijbregts P., et al., (1997) [47]	Northern and Southern Europe	Seven Countries Study	Healthy diet indicator (HDI)
26	Jarvandi S., et al., (2013) [48]	USA	NIH-AARP Diet and Health Study	HEI-2005
27	Jeurnink SM., et al., (2012) [49]	Northern and Southern Europe	EPIC	DDS
28	Kaluza, J., et al., (2009) [50]	Sweden	Cohort of Swedish Men	RFS; Non-RFS
29	Kant AK., et al., (1995) [51]	USA	NHANES Epidemiologic Follow-Up Study	DDS
30	Kant AK., et al., (2000) [52]	USA	BCDDP	RFS
31	Kant AK, et al., (2009) [53]	USA	NIH-American Association of Retired Persons cohort	Dietary behaviour score (DBS)
32	Kappeler R., et al., (2013) [54]	USA	NHANES III	HEI
33	Kenfield SA., et al., (2014) [55]	USA	HPFS	MMDS; aMED
34	Knoops KTB., et al., (2004) [56]	Northern and Southern Europe	HALE (European cohort) Survey in Europe on Nutrition and the Elderly: a concerned Action (SENECA) Finland, Italy the Netherlands elderly (FINE) study	MMDS
35	Kyro C., et al., (2013) [57]	Denmark	Diet, Cancer and Health cohort	Nordic food index
36	Lagiou P., et al., (2006) [58]	Sweden	Scandinavian Women’s Lifestyle and Health Cohort	MMDS
37	Lagiou P., et al., (2007) [59]	Sweden	Scandinavian Women’s Lifestyle and Health Cohort	LCHP
38	Lee M, et al., (2011) [60]	Taiwan	The Elderly Nutrition and Health Survey	ODI-R; DDS
39	Li W., et al., (2013) [61]	USA	NIH-AARP Diet and Health Study	HEI-2005; aMED
40	Li W., et al., (2014a) [62]	USA	NIH-AARP Diet and Health Study	HEI-2010; aMED
41	Li W., et al., (2014b) [20]	USA	NIH-AARP Diet and Health Study	HEI-2005; aMED
42	Mai V., et al., (2005) [63]	USA	BCDDP	RFS
43	Martinez-Gonzalez MA., (2012) [64]	Spain	Seguimiento Universidad de Navarra (SUN) Project	MMDS
44	McCullough ML., et al., (2000a) [65]	USA	HPFS	HEI-f
45	McCullough ML., et al., (2000b) [66]	USA	NHS	HEI-f
46	McCullough ML., et al., (2002) [67]	USA	NHS HPFS	AHEI; RFS
47	Michels KB., & Wolk A. (2002) [68]	Sweden	Mammography Screening Cohort	RFS; Non-RFS
48	Miller PE., et al., (2013) [69]	USA	NIH-AARP	DASH
49	Mitrou PN, et al., (2007) [70]	USA	NIH-AARP	tMED; aMED
50	Mursu J., et al., (2013) [71]	USA	Iowa Women’s Health Study	AHEI-2010
51	Nakamura Y., et al., (2009) [72]	Japan	National Integrated Project for Prospective Observation of Non-Communicable Diseases and its Trends in the Aged	Reduced-salt Japanese diet score
52	Nilsson LM., et al., (2012a) [73]	Sweden	VIP	Traditional Sami Diet Score
53	Nilsson LM., et al., (2012b) [74]	Sweden	VIP	LCHP score
54	Nilsson LM., et al., (2013) [75]	Sweden	VIP	LCHP score
55	Reedy J., et al., (2008) [13]	USA	NIH-AARP	HEI-2005; AHEI; MMDS; RFS
56	Reedy J., et al., (2013) [76]	USA	NIH-AARP	HEI-2010; AHEI-2010; aMED; DASH score
57	Seymour JD., et al., (2003) [77]	USA	American Cancer Society Cancer Prevention Study II Nutrition Cohort	DQI
58	Tognon G., et al., (2012) [78]	Sweden	VIP	mMDS
59	Trichopoulou A., et al., (2003) [79]	Greece	EPIC	MMDS
60	Trichopoulou A., et al., (2010) [80]	Greece	EPIC	MMDS
61	van Dam RM., et al., (2008) [81]	USA	NHS	AHEI
62	Vormund K., et al., (2014) [82]	Switzerland	Longitudinal cohort	MDS
63	Von Rueston A., et al., (2010) [83]	Germany	EPIC-Potsdam	GFPI
64	Zarrin R., et al., (2013) [84]	Australia	Nambour Skin Cancer study	Aussie-DQI

## Supplementary Materials

Supplementary materials can be found at http://www.mdpi.com/1422-0067/17/7/1052/s1.

## Appendix A

**Table A1:** Newcastle—Ottawa quality assessment scale.

Selection	
1. Representativeness of the exposed cohort (a). Truly representative of the average essentially healthy adult in the community (b). Somewhat representative of the average essentially healthy adult in the community (c). Selected group of users eg. nurses, volunteers (d). No description of the derivation of the cohort	
2. Selection of the non-exposed cohort (a). Drawn from the same community as the exposed cohort (b). Drawn from a different source (c). No description of the derivation of the non-exposed cohort	
3. Ascertainment of exposure (a). Secure record (eg surgical records) (b). Structured interview (24hr recall; or FR) (c). Written self-report (self-report FFQ) (d). No description	
4. Demonstration that outcome of interest was not present at start of study (a). Yes (b). No	
**Comparability**	
1. Comparability of cohorts on the basis of the design or analysis (a). Study statistically adjusts for three or more key variables from at least two of three categories: (1) Socio-demographic factors (e.g. race, sex, ethnicity, education, income, etc.; (2) Medical risk factors (e.g. age, previous cancers, diabetes history, screening history, etc.; (3) Health behaviours (e.g. smoking, alcohol intake, physical activity) (b). Study receives an additional if study adjusts for at least one variable from each category and 4 or more factors in total.	
**Outcome**	
1. Assessment of outcome (a). Independent blind assessment (b). Record linkage (c). Self-report (d). No description	
2. Was follow-up long enough for outcomes to occur (a). Yes (> 10 years) (b). No	
3. Adequacy of follow-up of cohorts (a). Complete follow-up—all subjects accounted for (b). Subjects lost to follow-up unlikely to introduce bias—small number lost (<15% loss to F/U), or description provides of those lost (c). Follow-up rate >15% and no description of those lost (d). No statement	
	Total Score
Comments	

A study can be awarded a maximum of one star for each numbered item within the Selection and Outcome categories. A maximum of two stars can be given for Comparability. Maximum possible score is nine stars and this is considered high quality.
