# Supplementary material for: Diet Quality and Cancer Outcomes in Adults: A Systematic Review of Epidemiological Studies
Source: Int J Mol Sci. 2016 Jul 5;17(7):1052. doi: 10.3390/ijms17071052 (PMC4964428; doi:10.3390/ijms17071052)
Supplement: Supplementary file 1 [file ijms-17-01052-s001.pdf]

Supplementary Materials: Diet Quality and Cancer Outcomes in Adults: A Systematic Review of Epidemiological Studies

Jennifer Potter, Leanne Brown, Rebecca L. Williams, Julie Byles and Clare E. Collins

Table S1. Diet quality indices identified in included studies.

	Index & Original Author	Origin	Type of Index	Objective	Index Method	Score Range	Dietary Method	Applied in the Following Studies
1	Diet Quality Index (DQI) (1) Seymour, J.D. (2003) [77].	USA	Food and nutrient	Based on the DQI defined by Patterson et al., (1994) which was created to reflect the quality of the diet based the US National Research Council Diet and Health recommendations. With modification for calcium recommendations.	Eight component score based on eight recommendations from the US National Research Council Diet and Health. Healthiest intakes scored as 0, intermediate scored as 1 and unhealthiest intakes scored as 2. Scores include % energy from total fat; % energy from saturated fat; cholesterol intake; fruit and vegetable intake; breads, cereals and legume intake; protein intake; sodium intake and calcium intake.	0–16 (0 indicates excellent diet)	FFQ	Seymour, J.D. (2003) [77]
2	Diet Quality Index (DQI) (2) Cuenca-Garcia, M., et al. (2014) [42].	USA	Food and nutrient	Based on the DQI defined by Patterson et al., (1994) which was created to reflect the quality of the diet based the US National Research Council Diet and Health recommendations.	New cut-off added for calcium and protein. Overall score based on eight recommendations from the US National Research Council Diet and Health. Unhealthiest intakes scored as 0, intermediate scored as 1 and healthiest intake scored as 2. Scores include % energy from total fat and saturated fat; cholesterol intake; fruit and vegetable intake; breads, cereals and legume intake; protein intake; sodium intake and calcium intake.	0–16 (16 indicates excellent diet)	3 d food record (2 weekdays and 1 weekend day)	Cuenca-Garcia, M., et al. (2014) [42]
3	Diet Quality Index Revised (DQIR) Haines, P., et al (1999) [94].	USA	Food and nutrient	Based on the DQI and adapted for the NHS.	Comprises 10 components (grains; vegetables; fruit; total fat; saturated fat; cholesterol; iron; calcium; diet diversity; and moderation in added fat and sugar) The total fat, saturated fat, and cholesterol components were calculated as a % total energy and scored categorically as 0, 5, or 10, and the remaining components were scored as continuous variables from 0 to 10, proportional to recommended range of intake. Scores summed across the 10 components for a highest possible score of 100 points.	0–100 (100 indicates excellent diet)	FFQ	Fung, T.T., et al. (2006) [45]
4	Healthy Eating Index (HEI) Kennedy, E., et al (1995) [95].	USA	Food and nutrient	Single summary estimate of adherence to the US Food Guide Pyramid and Dietary Guidelines for Americans.	Comprises 10 components. Five relate to the core foods given in the USDA Food Guide Pyramid (grains, vegetables, fruit, milk, meat) and additional components include total and saturated fat as a % of energy, cholesterol, sodium, and diet variety over 3 days. Scores for each component range from 0 (worst) to 10 (best).	0 (worst)–100 (best)	24 h recall and/2-d food record	Kappeler, R., et al. (2013) [54]

Table S1. *Cont.*

	Index & Original Author	Origin	Type of Index	Objective	Index Method	Score Range	Dietary Method	Applied in the Following Studies
5	Healthy Eating Index–f McCullough, M.L., et al. (2000) [65,66].	USA	Food and nutrient	Similar to the HEI however score calculated from FFQ data.	Similar to the HEI. Comprises 10 equally weighted components with each contributing 10 points toward total score. Components 1–5 based on serve recommendations from the USDA Food Guide Pyramid (vegetables, fruit, meat, milk, grains). Other components based on % energy from fat and sat fat, salt intake and cholesterol intake. A variety component considers number of unique foods consumed over the month. Components scored proportionately as continuous variables.	0 (worst)–100 (best)	FFQ	Fung, T.T., et al. (2006) McCullough, M.L., et al. (2000a) [65] McCullough, M.L., et al. (2000b) [66]
6	Alternate Healthy Eating Index (AHEI) (1) McCullough, M.L., et al. (2002) [67].	USA	Food and nutrient	Single score developed to assess whether the AHEI predicts chronic disease better than the original HEI.	Differed from the HEI scoring criteria by addressing quality within food groups by removing potatoes (including French fries), including nuts and soy, a ratio of white/red meat, trans fat, PUFA:SAT fat ratio, and adding sub-scores for multivitamin use and alcohol intake. Eight components (vegetables; fruit; nuts and soy; white:red meat ratio; cereal fibre; *trans* fat; alcohol and PUFA:SAT fat ratio) scored from 0 (worst) to 10 (best) with intermediate intakes scored proportionately between 0 and 10. Multivitamin use scored dichotomously with 2.5 points awarded for non-use and 7.5 points for use.	2.5 (worst)–87.5 (best)	Semi-quantitative FFQ	Fung, T.T., et al. (2006) [45]; McCullough, M.L., et al. (2002) [67]; Akbaraly, T. N., et al., (2011) [28]; Reedy, J., et al. (2008) [13]
7	Alternate Healthy Eating Index (AHEI) (2) Van Dam, R.M., et al. (2008) [81].	USA	Food and nutrient	Single score similar to McCullough (2002) with the exclusion of multivitamin use (as multivitamins have become a less important source of folate) and alcohol as alcohol was assessed separately.	Seven components (vegetables; fruit; nuts and soy; fish and poultry:red meat ratio; cereal fibre; *trans* fat; alcohol and PUFA:SAT fat ratio) scored from 0 (worst) to 10 (best) with intermediate intakes scored proportionately between 0 and 10.	0 (worst)–70 (best)	FFQ	Van Dam, R.M., et al. (2008) [81]
8	Healthy Eating Index 2005 (HEI-2005) (1) Guenther, P.M., et al. (2006) [96].	USA	Food and nutrient	Measure of overall compliance with the 2005 Dietary Guidelines for Americans.	Based on the 12 components of the MyPyramid food patterns. Nine components scored on adequacy, where highest score was assigned for meeting the guidelines: total fruit, including juice (0–5); whole fruit, excluding juice (0–5); total vegetables (0–5); dark green and orange vegetables and legumes (0–5); total grains (0–5); whole grains (0–5); milk (0–10); meat and beans (0–10); and oils (0–10). Three components based on moderation, where lower intakes scored higher: saturated fat (0–10); sodium (0–10); and % energy from fat, alcohol and added sugar (0–20). Total and sub-scores expressed per 1000 calories to account for differences in energy intake.	0 (no guidelines met) -100 (all guidelines met)	FFQ	Arem, H., et al. (2013) [29]; Bosire, C., et al. (2013) [18]; Li, W., et al. (2013) [61]; Li, W.-Q., et al. (2014 b) [20]; Jarvandi, S., et al. (2013) [48]; Reedy, J., et al. (2008) [13]

Table S1. *Cont.*

	Index & Original Author	Origin	Type of Index	Objective	Index Method	Score Range	Dietary Method	Applied in the Following Studies
9	Healthy Eating Index 2005 (HEI-2005) (2) Chiuve, S.E., et al. (2012) [39].	USA	Food and nutrient	Similar to above except scoring for sodium changed.	As for the 2005-HEI above except different scoring for sodium. Participants divided into 11 equal groups based on the distribution of reported sodium intakes and assigned corresponding scores of 0–10 (higher score for less sodium intake).	0–100	FFQ	Chiuve, S.E., et al. (2012) [39]
10	Healthy Eating Index-2010 (HEI-2010) Guenther et al., 2013 [97].	USA	Food and nutrient	Update the HEI to reflect the changes made in the 2010 Dietary Guidelines for Americans.	Comprises 12 components, 9 adequacy sub-scores (total fruit (0–5); whole fruit (0–5); total vegetables (0–5); greens and beans (0–5); whole grains (0–10); dairy (0–10); total protein foods (0–5); seafood and plant proteins (0–5); fatty acid ratio, PUFA:MUFA (0–10)) and 3 moderation sub-scores (refined grains (0–10); sodium (0–10); empty calories (0–20)). For adequacy components, intakes at the level of the standard or higher receive the maximum number of points available. For the moderation components, intakes at the level of the standard or lower receive the maximum number of points. All components (except fatty acid ratio) scored/100 kcal as a % of energy.	0 (worst)–100 (best)	FFQ	Reedy, J., et al. (2014) [76]; Li, W.Q., et al. (2014a) [62]
11	Alternate Healthy Eating Index-2010 (AHEI-2010) Chiuve, S.E., et al. (2012) [39].	USA	Food and nutrient	Update of the AHEI (Van Dam et al., 2008). Reflects current scientific evidence and based on foods and nutrients predictive of chronic disease risk. Changes include the addition of components for sodium, sugar-sweetened beverages, and omega-3 fatty acids.	Comprises 11 components each scored 0–10: vegetables; fruit; whole grains; sugar-sweetened beverages and juices; nuts and legumes; red/processed meat; *trans* fat; omega-3 fatty acids; PUFA; sodium; and alcohol. For women 10 points awarded for intake 0.5–1 std drinks/d and 0 points for ≥2.5 std drinks/d. For men 10 points awarded for intake 0.5–2 std drinks/d and 0 points for ≥3.5 std drinks/d.	0–110	124-item FFQ	Bosire, C., et al. (2013) [18]; Chiuve, S.E., et al. (2012) [39]; Mursu, J., et al. (2013) [71]; Reedy, J., et al. (2014) [76]
12	Recommended Food Score (RFS) (1) (Kant, et al., 2000) [52]	USA	Food	Single summary score based on adherence to the 1995 US Dietary Guidelines.	Dichotomous score based on intake (Y = 1 N = 0) of 23 food items (apples or pears; oranges; cantaloupe; orange or grapefruit juice; grapefruit; other fruit juices; dried beans; tomatoes; broccoli; spinach, mustard; turnip or collard greens; carrots or mixed vegetables with carrots; green salad; sweet potatoes, yams, other potatoes; baked or stewed chicken or turkey; baked or broiled fish; dark breads; cornbread, tortillas, grits; high fibre cereals; cooked cereals; 2% milk and 2% milk products; 1% skim milk). 1 point given for each item if the food is consumed at least once per week.	0–23	FFQ	Kant et al. (2000) [52]; Mai, V., et al. (2005) [63]; Reedy, J., et al. (2008) [13]
13	Recommended Food Score (RFS) (2) McCullough et al. 2002 [67].	USA	Food	Single summary score based on adherence to the 1995 US Dietary Guidelines.	As in the RFS scoring system outlined above. However because the FFQ used was longer the maximum and minimum possible scores increased.	0–56	FFQ	McCullough 2002 [67]; Fung, T.T., et al. (2006) [45]

Table S1. *Cont.*

	Index & Original Author	Origin	Type of Index	Objective	Index Method	Score Range	Dietary Method	Applied in the Following Studies
14	Recommended Food Score (RFS) (3) Kaluza, J., et al. (2009) [50].	Sweden	Food	Further develop a simple measure of diet quality that takes into account the variety of foods as recommended in dietary guidelines.	Update on RFS developed by Kant et al. (2000) to include more food items. Scoring based on 36 recommended food items. For recommended foods consumed at least 1–3 times per month participants assigned a score of 1. For consumption frequency less than this score of 0 was assigned.	0 (worst)–36 (best)	FFQ	Kaluza, J., et al. (2009) [50]
15	Recommended Food Score (RFS) (4) Michels & Wolk, 2002 [68].	Sweden	Food	Single summary score based on adherence to National Dietary Guidelines.	Differs to Kant et al. (2000) in 3 food items: juices; potatoes and chicken not included. A score of 1 assigned where intake over previous month of each of the following listed foods is between 1 and 3 occasions: apples/pears; citrus fruit; bananas; lettuce/cucumber; spinach/kale; tomatoes; cabbage; root vegetables; beans/peas; 0.5% or 1.5% fat milk; 1.5% fat yoghurt; whole grain bread or crisp bread (no fat); oats; salmon/herring/tuna; other fish.	0–18	FFQ	Michels & Wolk, 2002 [68]
16	Non-Recommended Food Score (1) Kaluza, J., et al. (2009) [50].	Sweden	Food	Provide a measure to examine mortality outcomes by poor diet quality.	Scoring based on 16 food items including red meat products (3 items); processed meat products (5 items); high fat dairy (3 items); white bread; sweets; potato chips/popcorn; mayonnaise; ice cream. Consumption of any of these non-recommended products 3 or more times per week assigned a score of 1. Score of 0 assigned for consumption below 3 times per week.	0 (healthier)–16 (most unhealthy)	FFQ	Kaluza, J., et al. (2009) [50]
17	Non-Recommended Food Score (2) Michels & Wolk, 2002 [68].	Sweden	Food	Provide a measure to examine mortality outcomes by poor diet quality.	Scoring based on 21 items. Consumption of any of these non-recommended products 3 or more times per month assigned a score of 1. Score of 0 assigned for consumption below 3 times per month.	0 (most healthy)–21 (most unhealthy)	FFQ	Michels & Wolk, 2002 [68]
18	Modified Mediterranean Diet Score (MMDS) (1) Trichopoulou, A., et al. (2003) [79].	Greece	Food	Indicate the degree of adherence to the Mediterranean diet.	Similar to the MDS outlined above with the addition of a fish component. Total of nine component scores. For beneficial components (vegetables; legumes; fruits and nuts; cereals; fish; high monounsaturated:saturated fatty acid ratio) intake above the sex-specific median cut-off assigned score = 1 and below = 0. For components presumed to be detrimental (meat and poultry; and dairy) persons whose intake was above the sex-specific median assigned score = 0 and below = 1. For alcohol, intake for men scored 1 for ethanol intake between 10 and 50 g/d and women scored 1 for intake between 5 and 25 g/d.	0–9	FFQ	Trichopoulou, A., et al. (2003) [79]; Ax, E., et al. (2014) [30]; Couto, E., et al. (2011) [40]; Benetou, V., et al. (2008) [32]; Kenfield, S.A., et al. (2014) [55]; Lagiou, P., et al. (2006) [58]; Martinez-Gonzalez, M.A., et al. (2012) [64]; Trichopoulou, A., et al. (2010) [80]

Table S1. *Cont.*

	Index & Original Author	Origin	Type of Index	Objective	Index Method	Score Range	Dietary Method	Applied in the Following Studies
19	Modified Mediterranean diet score (MMDS) (2) Reedy, J., et al. (2008) [13].	USA	Food	Single score to asses conformity to the traditional Mediterranean diet.	Nine dietary components used to capture the traditional Mediterranean diet. One point scored for intake above the sex-specific cohort median for the following components considered beneficial to health; whole grains; total vegetables (excluding potatoes); total fruit; fish; legumes; and nuts. One point given for intake below the sex-specific Mediterranean median for red and processed meat and monounsaturated fatty acid to saturated fatty acid ratio. A moderate intake of and 5–25 g/d awarded one point.	0–9	FFQ	Reedy, J., et al. (2008) [13]
20	Modified Mediterranean Diet Score (MMDS) (3) Trichopoulou, A., et al. (2003) [79].	Adapted from the MMDS model above (Greece) and applied to the EPIC-elderly study comprising participant from 10 European countries	Food	Indicate the degree of adherence to the Mediterranean diet.	Similar to the MMDS (1) above with the addition of polyunsaturates to the lipid ratio. Total of nine component scores. For beneficial components (vegetables; legumes; fruits and nuts; cereals; fish; high monounsaturated and polyunsaturated:saturated fatty acid ratio) intake above the sex-specific median cut-off assigned score = 1 and below = 0. For components presumed to be detrimental (meat and poultry; and dairy) persons whose intake was above the sex-specific median assigned score = 0 and below = 1. For alcohol, intake for men scored 1 for ethanol intake between 10 and 50 g/d and women scored 1 for intake between 5 and 25 g/d.	0–9	FFQ or FR	Couto E., et al. (2013) [41]; Bamia, et al., 2013 [10]
21	Modified Mediterranean Diet Score (MMDS) (4) Cade, J.E., et al. (2011) [38].	UK	Food and nutrient	Modified MMDS (1) with the addition of separate categories for meat and poultry.	Total of 10 component scores. Intakes above the cohort median cut-off for vegetables; legumes; fruit and nuts; cereal; fish and ratio of monounsaturated to saturated fatty acids, gave a score of 1. Considered non-beneficial components, intakes below the cohort median of meat; poultry; and dairy scored 1. For alcohol, women consuming 5–10 g ethanol/d received a score of 1.	0–10	FFQ	Cade, J.E., et al. (2011) [38]
22	Modified Mediterranean Diet Score (MMDS) (5) Knoops, K.T.B., et al., (2004) [56].	Europe (SENCA study), Finland, Italy, The Netherlands	Food and nutrient	A summary score to represent adherence to the Mediterranean diet.	Similar to Trichopoulou 2003 with alcohol component removed. Eight components. For beneficial components one point added for intake above the sex-specific median: MUFA:SAFA ratio; fruits and fruit products; vegetables and potatoes; legumes, nuts and seeds; fish; grains. For components not beneficial one point awarded for intake below the sex-specific median: meat and meat products; and dairy products.	0–8	Diet Hx	Knoops K.T.B., et al., (2004) [56]

Table S1. *Cont.*

	Index & Original Author	Origin	Type of Index	Objective	Index Method	Score Range	Dietary Method	Applied in the Following Studies
23	Modified Mediterranean Diet Score (MMDS) (6) Cuenca-Garcia M., et al., (2014) [42].	USA	Food	Indicate the degree of adherence to the Mediterranean diet Alcohol component scored differently to original MMDS.	Based on Trichopolou et al., 2003 outlined above. Total of nine component scores. For beneficial components (vegetables; legumes; fruits and nuts; cereals; fish; high monounsaturated:saturated fatty acid ratio) intake above the sex-specific median cut-off assigned score = 1 and below = 0. For components presumed to be detrimental (meat and poultry; and dairy) persons whose intake was above the sex-specific median assigned score = 0 and below = 1. For alcohol, intake for men of ≤2 drinks/d and women intake ≤1 drink/d scored 1.	0–9	FFQ	Cuenca-Garcia, M., et al., (2014) [42]
24	Mediterranean Diet Score (MDS) (1) Vormund, K., et al. (2014) [82].	Switzerland	Food	Modification of Trichopoulou, A., et al.'s (2003) MMDS to include dairy as a beneficial component; exclude nuts; legumes and include only whole grain cereals. Alcohol cut-offs the same for men and women.	Total of nine component scores: salad; vegetables; fruits; whole grains; white meat; fish monounsaturated lipids; dairy; and alcohol (wine). For each component a value of 0, 1 or 0.5 (the later for monounsaturated lipids) was assigned. Including beneficial components (salad; vegetables; fruits; whole grains; white meat; fish; monounsaturated lipids, wine) and avoiding detrimental components (red or processed meat) received score of 1 point for each component. One point also assigned for inclusion of dairy.	0 (worst)–9 (best)	24 h recall	Vormund, K., et al. (2014) [82]
25	CSMMDS Couto et al., 2011 [41].	EPIC	Food	Adaptation of Trichopoulou et al.'s MMDS. Based on cut-offs from sex and centre specific medians within the EPIC study.	Total of nine component scores. For beneficial components (vegetables; legumes; fruits and nuts; cereals; fish; high monounsaturated and polyunsaturated:saturated fatty acid ratio) intake above the centre and sex-specific median cut-off assigned score = 1 and below = 0. For components presumed to be detrimental (meat and poultry; and dairy) persons whose intake was above the centre and sex-specific median assigned score = 0 and below = 1. For alcohol, intake for men scored 1 for ethanol intake between 10 and 50 g/d and women scored 1 for intake between 5 and 25 g/d.	0–9	FFQ	Couto et al., 2011 [40]; Bamia et al., 2013 [10]
26	Alternate Mediterranean Diet Score (aMED) (1) Bosire, C., et al. (2013) [18].	USA	Food	Indicate the degree of adherence to the Mediterranean diet.	Modification of MMDS to exclude potato products from the vegetable group; separate fruits and nuts into two groups; eliminate the dairy group, include whole-grain products only; and include only red and processed meats in the meat group. For beneficial components (fruits; nuts; vegetables; legumes; whole grains; fish; and MUFA:SAT fat ratio) one point is given for intake above the population median. One point given for intake below the population median for unhealthy components (red and processed meats). For alcohol, intake between 10 and 25 g/d assigned 1 point for both men and women.	0–9	124-item FFQ	Bosire, C., et al. (2013) [18]; * note alcohol scoring different to aMED used by Fung et al. below

Table S1. *Cont.*

	Index & Original Author	Origin	Type of Index	Objective	Index Method	Score Range	Dietary Method	Applied in the Following Studies
27	Alternate Mediterranean Diet Score (aMED) (2) Fung, T.T., et al. (2006) [45].	USA	Food	Indicate the degree of adherence to the Mediterranean diet.	Modification of MMDS (1) to exclude potato products from the vegetable group; separate fruits and nuts into two groups; eliminate the dairy group, include whole-grain products only; and include only red and processed meats in the meat group. For beneficial components (fruits; nuts; vegetables [excluding potatoes]; legumes; whole grains; fish; and MUFA:SAT fat ratio) one point is given for intake above the population median. One point given for intake below the population median for unhealthy components (red and processed meats). For alcohol, intake between 5 and 15 g/d.	0 (worst)–9 (best)	FFQ	Fung, T.T., et al. (2006) [45]; Li, W., et al. (2013) [61]; Li, W. Q., et al. (2014a) [62]; Li, W.-Q., et al. (2014b) [20]; Mitrou, P.N., et al. (2007) [70]; Reedy, J., et al. (2014) [76]
28	Alternate Mediterranean Diet Score (aMED) (3) Fung, T.T., et al. (2010) [11].	USA	Food	Indicate the degree of adherence to the Mediterranean diet.	As above with the addition of sex-specific cut-offs for alcohol intake. Modification of MMDS to exclude potato products from the vegetable group; separate fruits and nuts into two groups; eliminate the dairy group, include whole-grain products only; and include only red and processed meats in the meat group. For beneficial components (fruits; nuts; vegetables [excluding potatoes]; legumes; whole grains; fish; and MUFA:SAT fat ratio) one point is given for intake above the population median. One point given for intake below the population median for unhealthy components (red and processed meats). For alcohol, intake between 5 and 15 g/d for women and 10–25 g for men assigned 1 point.	0–9	FFQ	Fung, T.T., et al. (2010) [11]; Kenfield, S.A., et al. (2014) [55]
29	Relative Mediterranean Diet Score (rMED) (2) Buckland, G., et al. (2010) [35].	EPIC (10 European countries)	Food	Provide a summary estimate of adherence to the Mediterranean diet.	A variation of the MDS and MMDS. Based on intake of 9 key components. Each component (excluding alcohol) measured as grams/1000 kcal/day and divided into tertile of intake. Participants in tertiles 1, 2 and 3 received a score of 0, 1 and 2 respectively based on their level of intake within the component category. Positive scoring for components presumed to be beneficial (fruit including nuts and seeds, excluding juice; vegetables, excluding potato; legumes; cereals; fish; and olive oil). Scoring was inverted for those components presumed to be non-beneficial to health (total meat and dairy). Alcohol scored as a dichotomous variable. Two points were assigned for moderate intake (5–25 g ethanol/d for women and 10–50 g/day ethanol for men) and 0 points for above and below the sex-specific ranges.	0–18	FFQ	Buckland, G., et al. (2014) [37]; Buckland, G., et al. (2010) [35]; Buckland, G., et al. (2011) [36]

Table S1. *Cont.*

	Index & Original Author	Origin	Type of Index	Objective	Index Method	Score Range	Dietary Method	Applied in the Following Studies
30	Adapted Relative Mediterranean Diet Score (arMED) (1) Buckland, G., et al. (2013) [17].	EPIC (10 European countries)	Food	Provide a summary estimate of adherence to the Mediterranean diet with the exclusion of alcohol as alcohol is a known risk factor for BC incidence.	Adapted version of rMED. arMED scoring based on the MDS and MMDS. For the six components presumed to be beneficial to health (fruits, vegetables, legumes, fish, olive oil, and cereals) participants were assigned a score of 0–2 based on country-specific tertiles of intake. This scoring was inverted for components presumed to be detrimental to health (meat and dairy).	0–16	FFQ	Buckland, G., et al. (2013) [17]
31	Refined Mediterranean Diet Score (refined mMDS) (2) Tognon, G., et al. (2012) [78].	Sweden	Food and nutrient	Provide a summary estimate of adherence to the Mediterranean diet.	Eight component score modified version of Tognon et al., (2011) with the exclusion of legumes, nuts and seeds component. For beneficial components (vegetables and potatoes; fruit and fresh juices; wholegrain cereals; fish and fish products; ratio of MUFA and PUFA to saturated fatty acids) one point is given for intake above the population median. One point given for intake below the population median for unhealthy components (red and processed meats; and dairy). For alcohol, moderate intake (primarily in the form of wine and generally consumed during meals) scored 1 point. Intake of each component adjusted for daily energy intakes of 2500 kcal for men and 2000 kcal for women.	0 (worst)–8 (best)	FFQ	Tognon, G., et al. (2012) [78]
32	Traditional Mediterranean Diet Score (tMED) Mitrou, P.N., et al. (2007) [70].	USA	Food and nutrient	Provide a summary estimate of adherence to the Mediterranean diet.	Similar to the MMDS (Trichopoulou, A., et al. 2003) but separates fruit and nuts into two groups, eliminates dairy, includes only whole-grains, red and processed meat and uses same alcohol cut-off for men and women. Total of nine component scores. For beneficial components (vegetables [excluding potatoes], legumes, fruits and nuts, grains, fish, high monounsaturated:saturated fatty acid ratio) intake above the sex-specific median cut-off assigned score = 1 and below = 0. For components presumed to be detrimental (meat and dairy) persons whose intake was above the sex-specific median assigned score = 0 and below = 1. For alcohol, intake for men scored 1 for ethanol intake between 10 and 50 g/d and women scored 1 for intake between 5 and 25 g/d.	0–9	FFQ	Mitrou, P.N., et al. (2007) [70]
33	Low Carbohydrate-High Protein Score (LCHP) (1) Ax, E., et al. (2014) [30].	Sweden	Nutrient	Provide a summary score of adherence to a low carbohydrate, low protein eating pattern.	Study participants divided into deciles of carbohydrate and protein intake. Participants in the highest decile of carbohydrate intake assigned the lowest score and those in the lowest the highest score. The reciprocal scoring was applied to protein intake.	2–20	7 d food record	Ax, E., et al. (2014) [30]

Table S1. *Cont.*

	Index & Original Author	Origin	Type of Index	Objective	Index Method	Score Range	Dietary Method	Applied in the Following Studies
34	Low Carbohydrate-High Protein Score (LCHP) (2) Fung, T.T., et al. (2010) [46].	USA	Nutrient	Provide a summary score of adherence to a low carbohydrate, low protein eating pattern.	Percent energy from fat, protein, and carbohydrate were divided equally into 11 categories according to percentiles. For fat and protein, those in the highest category received 10 points and those in the next received 9 points and so forth. A reciprocal scoring system was applied for carbohydrate intake.	Summation of the fat, protein and carbohydra te scores for a range of 0 (worst)–30 (best)	FFQ	Fung, T.T., et al. (2010) [46]; Fung, T.T., et al. (2011) [12]; Lagiou, P., et al., (2007) [59]
35	Low Carbohydrate-High Protein Score – vegetable (LCHP-V) Fung, T.T., et al. (2010) [46].	USA	Nutrient	Provide a summary score of adherence to a vegetable-based low carbohydrate, low protein eating pattern.	Based on % energy from carbohydrate, animal protein, and animal fat.	Not specified	FFA	Fung, T.T., et al. (2010) [46]
36	Low Carbohydrate-High Protein Score –animal based (LCHP-AB) Fung, T.T., et al. (2010) [46].	USA	Nutrient	Provide a summary score of adherence to a animal product based low carbohydrate, low protein eating pattern.	Based on % energy from carbohydrate, vegetable protein, and vegetable fat.	Not specified	FFA	Fung, T.T., et al. (2010) [46]
37	Low Carbohydrate-High Protein Score (LCHP) (3) Trichopoulou et al., (2007) [98].	Greece	Nutrient	Provide a summary score of adherence to a low carbohydrate, low protein eating pattern.	All participants sorted based on deciles of carbohydrate and protein intake. For each participant, ascending decile of protein intake and descending decile of carbohydrate intake were added to create the total LCHP score using, alternatively, absolute and energy-adjusted carbohydrate and protein values.	2 (worst)–20 (best)	FFQ	Nilsson, L.M., et al. (2012b) [74]; Nilsson, L. M., et al. (2013) [75]; Lagiou P., et al., 2007 [59]
38	DASH score (1) Fung, T.T., et al. (2008) [99].	USA		Create a score based on foods emphasized and minimised in the Dietary Approaches to Stop Hypertension eating pattern.	Comprises eight components. For those components considered beneficial to health (fruits; vegetables; nuts and legumes; low-fat dairy products; and wholegrains) participants are categorised into quintiles. Those in quintile 1 receive 1 point and those in quintile 5 receive 5 points. For those components considered detrimental to health (sodium; processed meats; and sweetened beverages), those in quintile 1 received 5 points and those in quintile 5 1 point.	8 (worst)–40 (best)	FFQ	Fung, T.T., et al. (2010) [11]; Fung, T. T., et al. (2011) [12]; Miller, P.E., et al. (2013) [69]; Reedy, J., et al. (2014) [76]
39	DASH index (2) Dixon et al., (2007) [100].	USA	Food and nutrient	Assess adherence to the DASH eating plan shown in the 2005 Dietary Guidelines for Americans.	Dichotomous score with 1 point awarded for meeting the guideline and 0 awarded otherwise. Total of nine components: total fruit; total vegetables; total whole grains; nuts, seeds, and legumes; meat intake, % energy from sugar; alcohol intake; and % energy from saturated fat.	0 (worst)–9 (best)	FFQ	Miller, P.E., et al. (2013) [69]

Table S1. *Cont.*

	Index & Original Author	Origin	Type of Index	Objective	Index Method	Score Range	Dietary Method	Applied in the Following Studies
40	DASH index (3) Mellen et al., (2008) [101].	USA	Nutrient	Totally nutrient-based to assess the target nutrient values from the DASH eating pattern.	Nine components of which are expected to be higher (protein, fibre, magnesium, calcium, and potassium) and lower (total fat, saturated fat, sodium, and cholesterol) on the DASH eating pattern. The method uses nutrient targets based on a 2100-cal diet for both men and women. Meeting the nutrient goal receives 1 point with 0 points awarded otherwise. For intakes that meet an intermediate goal ½ point is awarded.	0 (worst)–9 (best)	FFQ	Miller, P.E., et al. (2013) [69]
41	DASH index (4) Güenther [102].	USA	Food	Food-based index to measure adherence to the DASH eating plan shown in the 2005 Dietary Guidelines for Americans.	10 component score. Six components scored out of 10 (fruit (includes juice); vegetables (including potato); meat; poultry; fish; eggs; nuts, seeds, and legumes; fats and oils; and sweets). Four components on a 5 point scale (total grains; high fibre grains; total dairy; and low fat dairy). Maximum score awarded if individuals meet recommendations. Lower intakes scored proportionately. Recommendations based on 4 different energy intakes (1600, 2000, 2300, 3100 kcal/d) to account for different age, activity-level and sex-specific requirements.	0 (worst)–10 (best)	FFQ	Miller, P.E., et al. (2013) [69]
42	Diet Diversity Score (1) Büchner, F.L., et al. (2010) [34].	EPIC (10 European countries)	Foods	Provide a means to improve between-country comparability of fruit and vegetable diversity amongst participants in EPIC the DDS was created and applied.	Based on the baseline questionnaires four different DDS components derived: *DDSvegfr* counts the total number of different fruits and vegteables eaten over a two week period; *DDSveggr* one point given for eating each of the following at least once in a two week period (leafy vegetables; fruiting vegetables; root vegetables; cabbage; mushrooms; grain and pod vegetables; onion and garlic; and stalk vegetables); DDSvegpr counts the total number of different vegetable products eaten at least once in two weeks; *DDSfr* counts the total number of different fruit products eaten at least once in two weeks.	DDSvegfr 0–40 DDSveggr 0–8 DDSvegpr 0–26 DDSfr 0–14	FFQ FFQ + 7-d FR (UK)	Büchner, F.L., et al. (2011) [33]; Büchner, F.L., et al. (2010) [34]
43	Diet Diversity Score (2) Jeurnink, S. M., et al. (2012) [49].	EPIC (10 European countries)	Food	Provide a means to improve between-country comparability of fruit and vegetable diversity amongst participants in EPIC the DDS was created and applied.	As above. Grouping names changed: DDSvegfr DDSvegsub DDSveg DDSfruit	DDSvegfr 0–40 DDSveggr 0–8 DDSvegpr 0–26 DDSfr 0–14	FFQ	Jeurnink, S.M., et al. (2012) [49]
44	Diet Diversity Score (3) Lee, M.S., et al. (2011) [60].	Taiwan	Food	Simple summary estimate of diet diversity based on 6 core foods.	One point given for ≥0.5 serves per day for each of the following six food groups: dairy; eggs/legumes/meat/fish; grains; fruit; vegetables; oils/fats.	0 (worst)–6 (best)	24 h recall	Lee, M.S., et al. (2011) [60]

Table S1. *Cont.*

	Index & Original Author	Origin	Type of Index	Objective	Index Method	Score Range	Dietary Method	Applied in the Following Studies
45	Diet Diversity Score (4) Kant et al. (1991) [103].	USA	Food	Simple summary estimate of diet diversity based on 5 core foods.	One point given for eating food from each of the five food groups in the previous day. Food groups comprised: Dairy group (all milk and milk products); meat group (animal and plant protein sources); gain group (all grain products except cakes, pies, cookies and pastries); fruit group (all fresh, frozen, canned fruit juice, and dried fruits. Fruit drinks excluded); vegetable group (all raw, cooked, frozen and canned vegetables).	0 (worst)–5 (best)	24 h recall	Kant, A.K., et al. (1995) [51]
46	World Health Organisation Healthy Diet Indicator (HDI) (1) Huijbregts, P., et al. (1997) [47].	Netherlands, Italy & Finland	Food and nutrient	Provide a summary score using the dietary guidelines for the prevention of chronic diseases as defined by the WHO.	Dichotomous scoring system. A score of 1 was given if a participant was within the recommended range for each food/nutrient as defined by the WHO. Nine components: saturated fatty acids; polyunsaturated fatty acids; protein; complex carbohydrates; dietary fibre; fruits and vegetables; pulses, nuts, seeds; monosaccharides and disaccharides; cholesterol.	0 (worst)–9 (best)	Diet Hx	Huijbregts, P., et al. (1997) [47]
47	World Health Organisation Healthy Diet Indicator (HDI) (2) Cade, J.E., et al. (2011) [38].	UK	Food and Nutrient	Provide a summary score indicating adherence to the *WHO Diet, Nutrition and the Prevention of Chronic Diseases* report.	Dichotomous scoring system. A score of 1 was given if a participant was within the recommended sex-specific range for the specific nutrient outlined in the WHO guidelines and 0 assigned otherwise. Nutrients included in the scoring include; total fat, saturated fat, polyunsaturated fatty acids, total carbohydrate, non-milk extrinsic sugars, non-starch polysaccharides, fruit and vegetables, protein, cholesterol, and salt.	0 (worst)–10 (best)	FFQ	Cade, J.E., et al. (2011) [38]
48	World Health Organisation Healthy Diet Indicator (3) Berentzen, N.E., et al. (2013) [31].	Netherlands	Food and Nutrient	Provide an update of the HDI originally developed by Huijbregts (1997) to indicate adherence to the *WHO Diet, Nutrition and the Prevention of Chronic Diseases* report.	Dichotomous scoring system on seven variables. A score of 1 was awarded where intake met the WHO guidelines and 0 assigned otherwise. The seven components included: % energy from saturated fatty acids; % energy from polyunsaturated fatty acids; cholesterol intake; % energy from protein, dietary fibre intake; fruit and vegetable intake; and % energy from free sugars.	0 (worst)–7 (best)	FFQ	Berentzen, N.E., et al. (2013) [31]
49	Ideal Diet Index (IDI) Cuenca-Garcia, M., et al. (2014) [42].	USA	Food and nutrient	Index based on the DASH eating pattern for ideal cardiovascular health.	Dash eating pattern cut offs used to assign scores. One point given where intake met the guidelines for 8 components; fruit and vegetables; fish; whole grains; sodium; nuts, seeds, and legumes; added sugar; processed meat; and % energy from saturated fat. Score of 0 given if guideline not met.	0 (worst)–8 (best)	3-d food record (2 weekdays and 1 weekend day)	Cuenca-Garcia, M., et al. (2014) [42]

Table S1. *Cont.*

	Index & Original Author	Origin	Type of Index	Objective	Index Method	Score Range	Dietary Method	Applied in the Following Studies
50	DQI-SNR Drake I., et al., (2011) [104].	Sweden	Food and nutrient	A diet quality index (DQI) that assesses adherence to the Swedish nutrition recommendations (SNR) and the Swedish dietary guidelines.	The index comprises six components as outlined in the 2005 Swedish Nutrition Recommendations. Components comprise: % energy from saturated fatty acids and polyunsaturated fatty acids; fish and shellfish (g/wk); dietary fibre intake (g/MJ); fruit and vegetable intake (g/d); and % energy from sucrose. Model 1: uses pre-defined cut-offs based on the 2005 SNR and SDG.Cut-offs create dichotomous variables where adherence receives 1 point and non-adherence 0 points. Model 2: uses the study population's median energy-adjusted intakes as cut offs. One point assigned for intake above the population median for PUFA; fish and shellfish; dietary fibre; fruit and vegetables. One point assigned for intake below the median for SFA and sucrose. Model 3: ranked individuals into quintiles of energy-adjusted intake of the components. A proportional scale ranging from 1–5 was assigned to the different quintile groups, with high scores to individuals with higher intakes of PUFA, fish and shellfish; dietary fibre; and fruit and vegetables and lower scores for higher intakes of SFA and sucrose.	Model 1 0–6 Model 2 0–6 Model 3 6 (worst)–30 (best)	7-d menu book and 168-item FFQ	Drake, I., et al. (2012) [43]
51	Overall Dietary Index Revised (ODI-R) Lee, M.S., et al. (2011) [60].	Taiwan	Food and nutrient	Provide an overall summary estimate of adherence to the Dietary Guidelines and Food Guides for Taiwan.	ODI-R comprises 9 components: amount (10 points) and quality (5 points) of grains and starchy tubers; amount of vegetable (10 points) and fruit (10 points) intake; amount (10 points) and quality (5 points) of eggs/soy/fish/meats; amount of dairy intake (10 points); polyunsaturated:saturated fatty acid ratio (10 points); dietary cholesterol intake (10 points); refined sugar intake (3 points), salt intake (4 points); alcohol intake (3 points); and dietary diversity (at least 0.5 serving from 10 food groups, 0–10 points).	0 (worst)–100 (best)	24 h recall + FFQ	Lee, M.S., et al. (2011) [60]
52	Diet Behaviour Score (DBS) Kant, A.K., et al. (2009) [53].	USA		Develop a score that directly reflects the dietary behaviour recommendations from the Dietary Guidelines for Americans (2005).	Based on six behavioural recommendations from the 2005 Dietary Guidelines for Americans: usual weekly vegetable consumption; usual weekly fruit consumption; usual weekly consumption of whole grain breads and cereals; usual weekly consumption of low-fat dairy as a drink or with cereal; usual practice of addition of solid fat after cooking or at the table. Six points available for each component.	0 (worst)–36 (best)	FFQ	Kant, A.K., et al. (2009) [53]
53	Nordic Food Index Olsen et al. (2011) [105]	Denmark	Food	Develop a food-based index that includes only traditional Nordic foods which have expected health-promoting effects	Comprises six items: fish; cabbage; whole grain rye; whole grain oats; apples and pears; root vegetables. One point given for intake at or above the sex-specific median for the whole cohort for each food category.	0 (worst)–6 (best)	FFQ	Kyro, C., et al. (2013) [57]

Table S1. *Cont.*

	Index & Original Author	Origin	Type of Index	Objective	Index Method	Score Range	Dietary Method	Applied in the Following Studies
54	A priori diet quality score Mursu, J., et al. (2013) [71].	USA	food	Develop a food-based diet quality score to investigate relationships to overall and cause-specific mortality.	Based on dichotomous scoring for 34 food groups in a positively rated category, neutral category and a negative rated category. Scores for food groups either positively or negatively rated are categorised into quartiles. The a priori diet quality score was calculated by summing category scores for foods in the positively rated group (0–3) and the negatively rated group (3–0).	0 (worst)–81 (best)	FFQ	Mursu, J., et al. (2013) [71]
55	Reduced-Salt Japanese Diet Score Nakamura, Y., et al. (2009) [72].	Japan	Food	Design a score based on the traditional healthy Japanese diet.	Seven-component score with each component scored dichotomously. Components include egg intake (≤2 eggs/wk); fish intake (eaten ≥1 occasion over 2 days); meat intake (≤2 occasions/wk); tsukemono intake ≥1/d; infrequent intake soup with noodles; use of low-salt soya sauce; and moderate alcohol intake.	0 (worst)–7 (best)	FFQ	Nakamura, Y., et al. (2009) [72]
56	Traditional Sami Diet Score Nilsson, L. M., et al. (2013a) [73].	Sweden	Food and nutrient	Design a score based on the traditional Sami diet of the 1700s.	Eight-component score, One point scored for intake above the cohort median for red meat; fatty fish; total fat; berries; and boiled coffee. One point scored for intake below the cohort median for vegetables; bread; and fibre.	0 (lowest adherence) –8 (highest adherence)	FFQ	Nilsson, L.M., et al. (2013a) [73]
57	German Food Pyramid Index (GFPI) Von Ruesten, A., et al. (2010) [83].	Germany	Food	Develop a score to measure adherence to the German Food Pyramid recommended intakes.	Eight component score with each component having a possible maximum score of 10. Components include: non-alcoholic beverages; vegetables; fruits; cereals; dairy products; meat/sausages/fish/eggs; added fat/oil; and sweets/snacks. The components 'cereals', dairy', 'meat/sausages/fish/eggs', and added fat/oils' were negatively scored with higher intakes receiving lower scores. Other components positively scored. Extra points awarded for intakes above the recommended for the positively scored components.	0 (worst)–110 (best)	148 item-FFQ	Von Ruesten, A., et al. (2010) [83]
58	Aussie- DQI Zarrin, R., et al. (2013) [84]	Australia	Food and nutrient	A measure to reflect adherence to the Dietary Guidelines for Australian Adults and to assess risk factors associated with the Australian National Health Priority Areas.	Eleven component score. Components comprise: vegetables; fruits; dairy products; meat and alternatives; cereals; % energy from total fat and saturated fat; % energy from sugar; alcohol; processed meat; added sodium and dietary variety. The first 10 components scored from 0–10 points with maximum points awarded for meeting the guideline and proportional scores for intakes that deviate from the recommendations. Variety component comprises four sub-components (vegetables, fruit, wholegrain, and fish) each with scores between 0 and 4.	0 (worst)–120 (best)	24 h recall	Zarrin, R., et al. (2013) [84]
59	Diet quality score based on Nova Scotia DRIs Fitzgerald AL., et al. (2002) [44].	Canada	Nutrient	Create a diet quality score to assess adherence to the new Nova Scotia DRIs.	Assigned a value of 1 to each of 17 age and gender-specific nutrient recommendations that were met and 0 if not met (above or below recommendation).	0 (worst)–17 (best)	24 h recall	Fitzgerald A.L., et al. (2002) [44]

Table S2. Cancer incidence and diet quality score evidence table. F/U: Follow-up.

	Study	Location	Study Design & Cohort	Participants	Diet Quality Score	Method & Period of Dietary Analysis	Outcome Variable/s & Censoring	Cancer Incidence Results *	Overall Conclusions
1	Arem, H., et al., (2013) [29], The Healthy Eating Index 2005 and risk of pancreatic cancer in the NIH-AARP study	USA (California, Florida, Louisiana, New Jersey, North Carolina, Pennsylvania, Georgia, Michigan)	Cohort NIH-AARP study	N = 537,218 men (μ_{age} = 63 y) and women (μ_{age} = 62 y) residing in one of seven US states	HEI-2005 (1) (0–100)	FFQ returned in 1995 and 1996 (baseline). Dietary ax period preceding 12 mo *Validated*	Cox proportional hazards regression for HR and 95% Cis for incidence of pancreatic cancer Adjusted for sociodemographic, health status, and health behaviour factors *F/U from baseline to 2006.*	Median 10.5 y = F/U $HR_{(Q5VsQ1)}$ = 0.85; 95% CI (0.74, 0.97) *	Consuming a high quality diet (as scored by the HEI-2005) is linked to reduced risk of pancreatic cancer
2	Ax, E., et al., (2013) [30], Dietary patterns and prostate cancer risk: report from the population based ULSAM Cohort Study of Swedish Men	Sweden	Cohort prospective ULSAM	N = 1044 men (μ_{age} = 70 ± 1yr)	MMDS (1) (0–8) LCHP Score (1) (2–20) For each score, participants categorised into 3 groups (low, medium and high adherers)	7 d FR completed at baseline in 1991–1995	Cox proportional hazards regression for HR and 95% CI's Adjusted for energy intake, health behaviour, and socio-demographic factors *F/U from baseline to 2007*	Median F/U = 13.2 y mMDS $HR_{(high\ compared\ to\ low\ adherers)}$ = 1.04 (95% CI: 0.43, 2.49, P *trend* 0.90) LCHP $HR_{(medium\ compared\ to\ low\ adherers)}$ = 0.55 (95% CI: 0.32, 0.96, P *trend* 0.04) * $HR_{(high\ compared\ to\ low\ adherers)}$ = 0.47 (95% CI: 0.21, 1.04, P *trend* 0.04)	In this study a LCHP diet is linked to reduced risk of PCa
3	Bamia, C., et al., (2013) [10], Mediterranean diet and colorectal cancer risk: results from a European cohort [12] (8)	Denmark, France, Germany, Greece, Italy, the Netherlands, Norway, Spain, Sweden, and United Kingdom	Cohort EPIC	N = 143,752 men and N = 336,556 women enrolled in EPIC Age range of both men and women 25–70 y at baseline	Modified Mediterranean diet score (MMDS [3]) *(0–9)* Centre-Specific MMDS (CSMMDS) *(0–9)* Scores categorised lowest-highest as follows: 0–3; 4–5; 6–9	FFQ (multiple FFQs used at different centres completed at baseline 1992–2000)	Cox proportional hazards regression for HR and 95% CI's for CRC incidence Adjusted for energy intake, health behaviour, and socio-demographic factors *F/U from centre-baseline to centre census 2004–2008*	μ F/U = 11.6 y MMDS Pooled: HR = 0.89 (95% CI: 0.80, 0.99. P *trend* 0.02) Men: HR = 0.89 (95% CI: 0.76, 1.04. P *trend* 0.14) Women: HR = 0.88 (95% CI: 0.77, 1.01. P *trend* 0.05) CSMMDS Pooled: HR = 0.92 (95 % CI:0.84, 1.00. P *trend* 0.05) Men: HR = 0.91 (95% CI: 0.80, 1.03. P *trend* 0.14) Women: HR = 0.93 (95% CI: 0.83, 1.05. P *trend* 0.19)	Conformity to the Mediterranean diet is linked to reduced risk of CRC. After stratification by sex this association remained significant only for women

Table S2. *Cont.*

	Study	Location	Study Design & Cohort	Participants	Diet Quality Score	Method & Period of Dietary Analysis	Outcome Variable/s & Censoring	Cancer Incidence Results *	Overall Conclusions
4	Berentzen, N.E., et al., (2013) [31], Adherence to the WHO's Healthy Diet Indicator and overall cancer risk in the EPIC-NL cohort	Netherlands	Prospective cohort EPIC-NL (Prospect and MORGEN cohorts)	N = 35,355 men and women (μ age = 49.2 (±11.9) years)	HDI (3) Categorised into tertiles (scores 0–2; 3; 4–7)	178-item FFQ completed at baseline (1993–7) with data collected on intake over previous 12 mo *Validated*	Multivariate cox proportional hazards ratios for overall cancer risk HR and 95% Cis Adjusted for energy intake, health behaviour, and socio-demographic factors *F/U from baseline to 2007*	μ F/U = 12.7 y All participants $HR_{(T3VT1)}$ = 0.99 (95% CI: 0.96, 1.02. P *trend* 0.53) Men $HR_{(T3VT1)}$ = 0.95 (95% CI: 0.89, 1.03. P *trend* 0.46) Women $HR_{(T3VT1)}$ = 1.00 (95% CI: 0.95, 1.04. P *trend* 0.67)	Greater adherence to the WHOs HDI is not associated with reduced cancer risk
5	Benetou, V., et al., (2008) [32], Conformity to traditional Mediterranean diet and cancer incidence: the Greek Epic cohort	Greece	Longitudinal cohort EPIC	N = 25,623 men and women enrolled in EPIC (Greek segment)	MMDS (1) Categorised into tertiles: Poor adherence (scores 0–3); intermediate adherence (scores 4–5); and high adherence (scores 6–9)	150-item FFQ completed at baseline (1994–9) with data collected on intake over previous 12 mo *Validated*	Multivariate cox proportional hazards ratios for overall cancer risk HR and 95% CI's for cancer risk Adjusted for energy intake, health behaviour, and socio-demographic factors *F/U from baseline to 2007*	Median F/U = 9.7 y *All* $HR_{(T3VT1)}$ = 0.78 (95% CI: 0.64, 0.94) *Men* $HR_{(T3VT1)}$ = 0.83 (95% CI: 0.63, 1.09) *Women* $HR_{(T3VT1)}$ = 0.73 (95% CI: 0.56, 0.96)	Mediterranean diet as measured by the MMDS in this study is associated with reduced risk of cancer incidence (especially for women)
6	Bosire, C., et al., (2013) [18], Index-based dietary patterns and the risk of prostate cancer in the NIH-AARP diet and health study	USA (California, Florida, Louisiana, New Jersey, North Carolina, Pennsylvania, Atlanta, Georgia, and Detroit, Michigan)	Cohort, longitudinal NIH-AARP	N = 293,453 men (aged 50–71 y) from seven US states	HEI-2005 (0–100) aMED (0–9) AHEI-2010 (0–110) Categorised into quintiles	124-item FFQ completed at baseline (1995–6) Diet ax period 12 mo *Validated*	Cox proportional hazards regression for HR and 95% CI for risk of prostate cancer Adjusted for sociodemographic, health status, and health behaviour factors *F/U from baseline to 2006*	μ F/U = 8.9 y Weak associations were observed only among those men reporting recent PCA testing: HEI-2005 $HR_{(Q1VsQ5)}$ = 0.92 (95% CI:0.86, 0.98, P *trend* 0.01) AHEI-2010 $HR_{(Q1VsQ5)}$ = 0.93 (95% CI: 0.88, 0.99, P *trend* 0.05) aMED $HR_{(Q1VsQ5)}$ = 0.97 (95% CI: 0.91, 1.03, P *trend* 0.09)	Higher HEI-2005 and AHEI-2010 scores were associated with a lower risk of total PC only among men who reported PSA testing in the preceding 3 years.

Table S2. *Cont.*

	Study	Location	Study Design & Cohort	Participants	Diet Quality Score	Method & Period of Dietary Analysis	Outcome Variable/s & Censoring	Cancer Incidence Results *	Overall Conclusions
7	Buchner, F.L., et al., (2011) [33], Variety in vegetable and fruit consumption and risk of bladder cancer in the European Prospective Investigation into Cancer and Nutrition	Northern and Southern Europe (Denmark, France, Germany, Greece, Italy, The Netherlands, Norway, Spain, Sweden, UK)	Cohort longitudinal EPIC	N = 452,185 men and women enrolled in EPIC (aged 25–70 y)	DDS (1) categorised into sub-scores as follows: DDSvegfr (0–8) DDSveggr (0–8) DDSvegpr (0–26) DDSfr (0–14)	FFQ—specific to the country Diet ax period previous 12 mo completed at centre baseline (1991–2000) *Validated*	Cox proportional hazards regression for HR and 95% CI's for bladder cancer risk Adjusted for sociodemographic, health status, and health behaviour factors *F/U from centre-baseline to centre census 2002–2005*	μ follow-up = 8.7 y DDSvegfr $HR_{(tertile\ 3\ V\ tertile\ 1)}$ = 1.30 (95% CI: 1.00, 1.69. P *trend* 0.05) DDSveggr $HR_{(tertile\ 3\ V\ tertile\ 1)}$ = 1.19 (95% CI: 0.94, 1.51. P *trend* 0.43) DDSvegpr $HR_{(tertile\ 3\ V\ tertile\ 1)}$ = 1.16 (95% CI: 0.90, 1.50. P *trend* 0.26) DDSfr $HR_{(tertile\ 3\ V\ tertile\ 1)}$ = 0.99 (95% CI: 0.77, 1.28. P *trend* 0.98)	No conclusion of a link between DDS and BC risk. Small suggestion of a link to increased risk of bladder cancer for DDSvegfr, mostly among never-smokers. ($HR_{(tertile\ 3\ V\ tertile\ 1)}$ = 1.72, 95% CI: 1.00, 2.97. P *trend* 0.05) and namely among men ($HR_{(tertile\ 3\ V\ tertile\ 1)}$ = 2.22, 95% CI: 0.88, 5.57). Higher DDSfrveg scores in women linked to reduced risk ($HR_{(tertile\ 3\ V\ tertile\ 1)}$ = 0.74, 95% CI: 0.49, 1.11. P *trend* 0.12) particularly among ever smokers ($HR_{(tertile\ 3\ V\ tertile\ 1)}$ = 0.55, 95% CI: 0.32, 0.97. P *trend* 0.03)

Table S2. *Cont.*

	Study	Location	Study Design & Cohort	Participants	Diet Quality Score	Method & Period of Dietary Analysis	Outcome Variable/s & Censoring	Cancer Incidence Results *	Overall Conclusions
8	Buchner, F.L., et al., (2010) [34], Variety in vegetable and fruit consumption and risk of lung cancer in the European Prospective Investigation into Cancer and Nutrition	Northern and Southern Europe (Denmark, France, Germany, Greece, Italy, The Netherlands, Norway, Spain, Sweden, UK)	Cohort longitudinal EPIC	N = 452,187 men and women enrolled in EPIC (aged 25–70 y)	DDSvegfr (0–8) DDSveggr (0–8) DDSvegpr (0–26) DDSfr (0–14)	FFQ—specific to the country Diet ax period previous 12 mo completed at centre baseline (1991–2000) *Validated*	Cox proportional hazards regression for HR and 95% CI's for lung cancer risk Adjusted for sociodemographic, health status, and health behaviour factors *F/U from centre-baseline to centre census 2002–2005*	μ follow-up = 8.7 y DDSvegfr $HR_{(tertile\ 3\ V\ tertile\ 1)}$ = 0.96 (95% CI: 0.75, 1.21. P *trend* 0.65) DDSveggr $HR_{(tertile\ 3\ V\ tertile\ 1)}$ = 0.77 (95% CI: 0.64, 0.94. P *trend* 0.02) DDSvegpr $HR_{(tertile\ 3\ V\ tertile\ 1)}$ = 0.94 (95% CI: 0.74, 1.18. P *trend* 0.31) DDSfr $HR_{(tertile\ 3\ V\ tertile\ 1)}$ = 0.94 (95% CI: 0.76, 1.17. P *trend* 0.42)	Variety in vegetable consumption was inversely associated with lung cancer risk. However, after stratification by smoking status, HR remained significant only for current smokers ($HR_{(Q4vsQ1)}$ = 0.73, 95% CI: 0.57, 0.93. P *trend* 0.04) and not for former ($HR_{(Q4vSQ1)}$ = 0.86, 95% CI: 0.59, 1.26, P *trend* 0.70) or never smokers ($HR_{(Q4vSQ1)}$ = 0.81, 95% CI: 0.42, 1.59, P *trend* 0.90).
9	Buckland, G., et al., (2013) [17], Adherence to the Mediterranean diet and risk of breast cancer in the European Prospective Investigation into Cancer and Nutrition cohort study	Northern and Southern Europe (Denmark, France, Germany, Greece, Italy, The Netherlands, Norway, Spain, Sweden, UK)	Cohort longitudinal EPIC	N = 335, 062 women (aged 25–70 y)	arMED (1) (0–16) Score categorised into 3 groups: High (10–16) Medium (6–9) Low (0–5)	FFQ—specific to the country completed at baseline (1992–2000) Diet ax period previous 12 mo *Validated*	Cox proportional hazards regression for HR and 95% CI's for breast cancer risk by arMED scores Adjusted for sociodemographic, health status, and health behaviour factors F/U from baseline to 2002–10	μ F/U = 11 y For all women, a high compared to low arMED score associated with 6% reduction in BC risk (HR = 0.94; 95% CI:0.88, 1.00. P *trend* 0.048) For post-menopausal women high arMED score associated with 7% reduction in BC risk (HR = 0.93; 95% CI: 0.87, 0.99. P *trend* 0.037) No associations amongst pre-menopausal women	Higher adherence to a Mediterranean diet is associated with a lower BC risk amongst post-menopausal women. Amongst post-menopausal women, those with higher arMED scores had a 20% reduced risk of BC (HR = 0.80; 95% CI: 0.65, 0.99. P *trend* 0.043).

Table S2. *Cont.*

	Study	Location	Study Design & Cohort	Participants	Diet Quality Score	Method & Period of Dietary Analysis	Outcome Variable/s & Censoring	Cancer Incidence Results *	Overall Conclusions
10	Buckland, G., et al., (2014) [37], Adherence to the Mediterranean diet and risk of bladder cancer in the EPIC cohort study	Northern and Southern Europe (Denmark, France, Germany, Greece, Italy, The Netherlands, Norway, Spain, Sweden, UK)	Cohort longitudinal EPIC	N = 477,312 men and women enrolled in EPIC	rMED (2) (0–18) Score categorised into 3 groups: High (scores 11–18); Medium (scores 7–10); Low (scores 0–6)	FFQ—specific to the country completed at baseline (1992–2000) Diet ax period previous 12 mo *Validated*	Cox proportional hazards regression for HR and 95% CI's for bladder cancer risk Adjusted for sociodemographic, health status, and health behaviour factors F/U from baseline to 2002–10	μ F/U = 11 y No association between bladder cancer risk and rMED score. $HR_{(high\ V\ low\ rMED)}$ = 0.84 (95% CI: 0.69, 1.03. P *trend* 0.107) Amongst current smokers 34% reduction in risk of bladder cancer $HR_{(high\ V\ low\ rMED)}$ = 0.66 (95% CI: 0.47, 0.93. P *trend* 0.043)	No evidence for a link between the Mediterranean dietary pattern and bladder cancer risk
11	Buckland, G., et al., (2010) [35] Adherence to a Mediterranean Diet and risk of gastric adenocarcinoma within the European Prospective Investigation into Cancer and Nutrition cohort study	Northern and Southern Europe (Denmark, France, Germany, Greece, Italy, The Netherlands, Norway, Spain, Sweden, UK)	Prospective cohort EPIC	N = 485,044 men and women aged 35–70 y at baseline	rMED (2) Categorised into tertiles (T1-T3): low adherence (scores 0–6); medium adherence (scores 7–10); and high adherence (scores 11–18)	FFQ—specific to the country completed at baseline (1992–8) *Validated*	Cox proportional hazards regression for HR and 95% CI's for gastric adenocarcinoma (GC) risk Adjusted for sociodemographic, health status, and health behaviour factors *F/U from baseline to 2003–06*	μ F/U = 8.9 y $HR_{(T3VT1)}$ = 0.67 (95% CI: 0.47, 0.94. P *trend* 0.02)	Greater adherence to rMED is associated with reduced risk of GC in this cohort

Table S2. *Cont.*

	Study	Location	Study Design & Cohort	Participants	Diet Quality Score	Method & Period of Dietary Analysis	Outcome Variable/s & Censoring	Cancer Incidence Results *	Overall Conclusions
12	Cade, J.E., et al., (2011) [38], Does the Mediterranean dietary pattern or the Healthy Diet Index influence the risk of breast cancer in a large British cohort of women?	Brittan	Longitudinal cohort UKWCS	N = 33,731 women (aged 35–69 y)	MMDS (4) (0–10) HDI (2) (0–10) Both MMDS and HDI scores transformed into categorical variables with 6 categories (0–2[reference], 3, 4, 5, 6, 7–10)	219-item FFQ completed at baseline (1995–8) *Validated*	Cox proportional hazards regression for HR and 95% CI's for breast cancer risk Adjusted for sociodemographic, health status, and health behaviour factors *F/U from baseline to 2006*	μ F/U 9 y MMDS *All women* HR $_{(high\ Vs\ low\ scores)}$ = 0.96 (95% CI: 0.70, 1.32. P *trend* 0.4) *Premenopausal* HR $_{(high\ Vs\ low\ scores)}$ = 0.65 (95% CI: 0.47, 1.02. P *trend* 0.09) Menopausal HR $_{(high\ Vs\ low\ scores)}$ = 1.30 (95% CI: 0.83, 2.05. P *trend* 0.90) HDI *All women* HR $_{(high\ Vs\ low\ scores)}$ = 0.94 (95% CI: 0.67, 1.32. P *trend* 0.8) *Premenopausal* HR $_{(high\ Vs\ low\ scores)}$ = 0.83 (95% CI: 0.50, 1.39. P *trend* 0.60) Menopausal HR $_{(high\ Vs\ low\ scores)}$ = 0.99 (95% CI: 0.63, 1.55. P *trend* 0.90)	This study offers no evidence supporting a link between risk of breast cancer and the Mediterranean dietary pattern or WHO HDI. Small indication of a slightly reduced risk of breast cancer among premenopausal women with high compared to low MMDS scores.
13	Chiuve, S.E., et al., (2012) [39], Alternative dietary indices both strongly predict risk of chronic disease (8)	USA	Cohort NHS HPFS	N = 71, 495 women from the NHS (aged 30–55 y at baseline in 1976) N = 41, 029 men from the HPFS (aged 40–75 y at baseline in 1986)	HEI—2005 (2) *(0–100)* AHEI-2010 *(110)*	NHS : FFQ, in 1984 and 1986, then every 4 years to 2006 (7 total) HPFS–FFQ every 4 years from 1986–2006 (5 total) FFQs *validated* and collect data on preceding 12 mo	Cox proportional hazards models used to calculate HR and then estimate RR incidence of chronic diseases (including all-sites cancer) Adjusted for socio-demographic, health status, and health behaviour factors *F/U from baseline to 2008*	Up to 24 y F/U For all-sites cancer incidence: HEI-2005 Men: RR $_{(Q1\ vs\ Q5)}$ = 0.86 (95% CI: 0.64,0.98. P *trend* 0.003) Women: RR $_{(Q1\ vs\ Q5)}$ = 0.93 (95% CI: 0.87,0.98. P *trend* 0.04) Pooled: RR $_{(Q1\ vs\ Q5)}$ = 0.90 (95% CI: 0.84,0.96. P *trend* 0.001) HEI-2010 Men: RR $_{(Q1\ vs\ Q5)}$ = 0.94 (95% CI:0.87, 1.03. P *trend* 0.13) Women: RR $_{(Q1\ vs\ Q5)}$ = 0.93 (95% CI:0.88, 0.99. P *trend* 0.01) Pooled: RR $_{(Q1\ vs\ Q5)}$ = 0.94 (95% CI:0.89, 0.98) *	Both the HEI-2005 and AHEI-2010 were associated with a reduction in cancer risk for the pooled group of men and women (10% and 6% reductions respectively). Both the HEI-2005 and AHEI-2010 were inversely associated with cancer risk in women. For men, only the HEI-2005 showed a significant decrease in risk.

Table S2. *Cont.*

	Study	Location	Study Design & Cohort	Participants	Diet Quality Score	Method & Period of Dietary Analysis	Outcome Variable/s & Censoring	Cancer Incidence Results *	Overall Conclusions
14	Coutok E., et al., (2011) [40], Mediterranean dietary pattern and cancer risk in the EPIC cohort	Northern and Southern Europe (Denmark, France, Germany, Greece, Italy, The Netherlands, Norway, Spain, Sweden, UK)	Cohort longitudinal EPIC	N = 142,605 men N = 335,873 women EPIC cohort Aged 25–70 y at baseline between 1992 and 2000	MMDS (1) Categorised as follows (0–3; 4; 5; 6–9)	FFQ and 7 or 14 d FR Completed at baseline and FFQs measured intake over the previous 12 mo FFQ *validated*	Cox proportional hazards regression for HR and 95% CI's for total cancer risk Adjusted for socio-demographic, health status, and health behaviour factors *F/U from baseline to 2002–05*	μ F/U = 8.7 y Overall, pooled HR for risk of all cancers associated with a 2 point increment in MMDS = 0.96 (95% CI: 0.95, 0.98) Comparing MMDS scores of 6–9 to 0–3 Pooled: HR = 0.93 (95% CI: 0.90, 0.96. P *trend* 0.00001) Men: HR = 0.93, (95% CI: 0.88, 0.99. P *trend* 0.02) Women: HR = 0.93, (95% CI: 0.89, 0.96. P *trend* 0.0001)	In this study, adherence to a Mediterranean dietary pattern appears to reduce risk of overall cancer incidence.
15	Couto, E., et al., (2013) [41], Mediterranean dietary pattern and risk of breast cancer	Sweden	Longitudinal cohort Swedish WLH cohort	N = 49,258 women (aged 30–49 y at baseline in 1991–92)	MMDS (3) (0–9) Categorised as follows (0–2, 3–4, 5, 6–7, 8–9)	80-item FFQ completed at baseline (1991–2) Diet ax period 6 mo preceding *Validated*	Cox proportional hazards models used to calculate HR and then estimate RR incidence of breast cancer Adjusted for socio-demographic, health status, and health behaviour factors *F/U from baseline to 2008*	Mean F/U = 16 y Comparing high (8–9) with low (0–2) MMDS scores Full cohort: RR = 1.42, 95% CI: 0.99, 2.03. P *trend* 0.12 RR BC risk associated with a 2-point increment in MDS RR = 1.08 (95% CI: 1.00, 1.15) No change in risk association by menopausal status or tumour characteristics (e.g., receptor status). No significant associations if alcohol excluded from score.	Adherence to a Mediterranean diet did not reduce risk of breast cancer in this cohort of relatively young women
16	Fitzgerald, A.L, et al., (2002) [44], Diet quality and cancer incidence in Nova Scotia, Canada	Nova Scotia, Canada	Longitudinal cohort using participants in the Nova Scotia Nutrition Survey	N = 2108 men and women (μ age = 48.9 ± 15.7 at baseline in 1990)	Diet quality score based on the Nova Scotia DRIs (score range 0–17) Scores categorised into quantiles (Q1-Q4)	24-h recall completed at baseline	Nested case-control comparison. Multivariate OR and 95% CI's calculated for cancer risk Adjusted for socio-demographic, health status, and health behaviour factors *F/U from baseline to 1999*	Up to 9 y F/U Men $OR_{(Q4 \text{ V } Q1)}$ = 0.81 (95% CI: 0.40, 1.64. P *trend* 0.41) Women $OR_{(Q4 \text{ V } Q1)}$ = 0.94 (95% CI: 0.44, 2.00. P *trend* 0.54)	The OR indicates a lower risk of cancer for higher diet quality but this relationship is not statistically significant

Table S2. *Cont.*

	Study	Location	Study Design & Cohort	Participants	Diet Quality Score	Method & Period of Dietary Analysis	Outcome Variable/s & Censoring	Cancer Incidence Results *	Overall Conclusions
17	Fung, T.T., et al., (2010) [11], The Mediterranean and Dietary Approaches to Stop Hypertension (DASH) diets and colorectal cancer	USA	Longitudinal cohort<br>NHS<br>HPFS	N = 87,256 women aged 34–55 y at baseline in 1980<br>N = 45,490 men aged 40–75 y at baseline in 1986	aMED (3)<br>DASH score (1)<br>Both categorised into quintiles	FFQ Self-administered at baseline<br>Diet ax period preceding 12 mo<br>*Validated*	Cox proportional hazards regression for RR and associated 95% Cis for risk of overall colorectal cancer, colon cancer and rectal cancer<br>Adjusted for Health status factors and health behaviour variables<br>*F/U from baseline to 2006*	Pooled mean F/U = 8.35 y<br>NHS ≤26 y F/U<br>*Colorectal cancer*<br>aMED: $RR_{(Q5VQ1)}$ = 0.88<br>(95% CI: 0.74, 1.05. P *trend* 0.14)<br>DASH: $RR_{(Q5VQ1)}$ = 0.80<br>(95% CI: 0.67, 0.94. P *trend* 0.005) *<br>*Colon cancer*<br>aMED: $RR_{(Q5VQ1)}$= 0.91<br>(95% CI: 0.74, 1.11. P *trend* 0.13)<br>DASH: $RR_{(Q5VQ1)}$ = 0.70<br>(95% CI: 0.55, 0.90. P *trend* 0.002) *<br>*Rectal cancer*<br>aMED: $RR_{(Q5VQ1)}$ = 0.80<br>(95% CI: 0.55, 1.15. P *trend* 0.64)<br>DASH: $RR_{(Q5VQ1)}$ = 0.74<br>(95% CI: 0.52, 1.07. P *trend* 0.34)<br>HPFS ≤20 y F/U<br>*Colorectal cancer*<br>aMED: $RR_{(Q5VQ1)}$ = 0.88<br>(95% CI: 0.71, 1.09. P *trend* 0.25)<br>DASH: $RR_{(Q5VQ1)}$ = 0.81<br>(95% CI: 0.66, 1.00. P *trend* 0.09)<br>*Colon cancer*<br>aMED: $RR_{(Q5VQ1)}$ = 0.87<br>(95% CI: 0.67, 1.13. P *trend* 0.45)<br>DASH: $RR_{(Q5VQ1)}$ = 0.82<br>(95% CI: 0.63, 1.05. P *trend* 0.10)<br>*Rectal cancer*<br>aMED: $RR_{(Q5VQ1)}$ = 0.75<br>(95% CI: 0.46, 1.23. P *trend* 0.19)<br>DASH: $RR_{(Q5VQ1)}$ = 0.89<br>(95% CI: 0.72, 0.45. P *trend* 0.64)<br>Pooled data<br>(men & women total CRC risk)<br>aMED: $RR_{(Q5VQ1)}$ = 0.89<br>(95% CI: 0.77, 1.01. P *trend* 0.06)<br>DASH: $RR_{(Q5VQ1)}$ = 0.80<br>(95% CI: 0.70, 0.91. P *trend* 0.001) *	Adherence to the DASH dietary pattern (which involves higher intake of whole grains, fruit and vegetables, moderate amounts of low-fat dairy; and lower amounts of red and processed meat, desserts and sweetened beverages) is associated with reduced risk of CRC. Especially for women

Table S2. *Cont.*

	Study	Location	Study Design & Cohort	Participants	Diet Quality Score	Method & Period of Dietary Analysis	Outcome Variable/s & Censoring	Cancer Incidence Results *	Overall Conclusions
18	Fung, T.T., et al., (2011) [12], Low-carbohydrate diets, Dietary Approaches To Stop Hypertension diets, and the risk of postmenopausal breast cancer	USA	Longitudinal cohort NHS	N = 86,621 women aged 34–55 y at baseline in 1980	LCHP score (2) LCHP-V LCHP-AB DASH score (1) Categorised into quintiles	FFQ Self-administered at baseline assessed intake over preceding 12 mo *Validated*	Cox proportional hazards regression for RR and associated 95% Cis for risk of overall and receptor-specific breast cancer Adjusted for health status and health behaviour factors *F/U from baseline to 2006*	Up to 26 y F/U LCHP score $RR_{(Q5VQ1)}$ = 1.02 (95% CI: 0.93, 1.11. P *trend* 0.92) LCHP-V $RR_{(Q5VQ1)}$ = 0.95 (95% CI: 0.87, 1.03. P *trend* 0.16) LCHP-AB $RR_{(Q5VQ1)}$ = 1.02 (95% CI: 0.94, 1.11. P *trend* 0.81) DASH $RR_{(Q5VQ1)}$ = 0.97 (95% CI: 0.89, 1.06. P *trend* 0.98)	Inverse relationship observed for incidence of oestrogen-receptor negative breast cancer and DASH score ($RR_{(Q5VQ1)}$ = 0.80 (95% CI: 0.64, 1.01. P *trend* 0.02)
19	Fung, T.T., et al., (2006) [45], Diet quality is associated with risk of estrogen-receptor-negative breast cancer in post-menopausal women	USA	Longitudinal cohort NHS	N = 71,058 women aged 34–55 y at baseline in 1984	HEI-f AHEI (1) DQIR RFS (2) aMED (2) Categorised into quintiles	FFQ Self-administered at baseline and 5 times between 1984 and 1998 assessed intake over preceding 12 mo *Validated*	Cox proportional hazards regression for RR and associated 95% CI's for risk of oestrogen receptor negative breast cancer Adjusted for health status and health behaviour factors *F/U from baseline to 2002*	Up to 18 y F/U No association between any score and total post-menopausal and ER+ breast cancer Risk of ER- breast cancer HEI-f: $RR_{(Q5VQ1)}$ = 0.92 (95% CI: 0.68, 1.24. P *trend* 0.47) AHEI: $RR_{(Q5VQ1)}$ = 0.78 (95% CI: 0.59, 1.04. P *trend* 0.01) DQIR $RR_{(Q5VQ1)}$ = 0.97 (95% CI: 0.72, 1.31. P *trend* 0.35) RFS: $RR_{(Q5VQ1)}$ = 0.69 (95% CI: 0.51, 0.94. P *trend* 0.003) * aMED: $RR_{(Q5VQ1)}$ = 0.79 (95% CI: 0.60, 1.03. P *trend* 0.03) *	Women who scored higher on the AHEI, RFS and the AMED had a lower risk of ER-BC. NO association with ER+ BC or total post-menopausal cancer
20	Jarvandi, S., et al., (2013) [48], Increased risk of colorectal cancer in Type 2 diabetes is independent of diet quality	USA	Longitudinal cohort NIH-AARP Diet and Health Study	N = 484,020 men and women aged 50–70 y at baseline in 1995–6	HEI-2005 (1) Categorised into quartiles (Q1-Q4)	124-item FFQ self-administered, data collected over previous 12 mo. Completed at baseline *Validated*	Cox proportional hazards models used to calculate HR and 95% CI for colorectal cancer risk Adjusted for socio-demographic, health and health behaviour factors *F/U from baseline to 2006*	µ F/U = 9.2 y All $HR_{(Q1VQ4)}$ = 1.35 (95% CI: 1.26, 1.44) * Men $HR_{(Q1VQ4)}$ = 1.37 (95% CI: 1.26, 1.24) * Women $HR_{(Q1VQ4)}$ = 1.30 (95% CI: 1.15, 1.46) *	Diabetes and poor diet independently and additively are associated with colorectal cancer risk

Table S2. *Cont.*

	Study	Location	Study Design & Cohort	Participants	Diet Quality Score	Method & Period of Dietary Analysis	Outcome Variable/s & Censoring	Cancer Incidence Results *	Overall Conclusions
21	Jeurnink, S.M., et al., (2012) [49], Variety in vegetable and fruit consumption and the risk of gastric and esophageal cancer in the European Prospective Investigation into Cancer and Nutrition	Northern and Southern Europe (Denmark, France, Germany, Greece, Italy, The Netherlands, Norway, Spain, Sweden, UK)	Cohort longitudinal EPIC	N = 452,269 men and women aged 35–70 at baseline in 1992–2000	DDS (2) comprised 4 components: DDSvegfr DDSvegsub DDSveg DDSfruit Categorised into tertiles (T1-T3)	FFQ—specific to the country Diet ax period 12 mo prior *Validated*	Cox proportional hazards models used to calculate HR and then estimate RR incidence of gastric and esophageal cancer Adjusted for socio-demographic, health behaviour and other health factors *F/U from baseline to 2002–05*	μ F/U = 8.4 y *Non-cardia adenocarcinoma* DDSvegfr :$HR_{(T3VT1)}$ = 1.82 (95% CI: 1.02, 3.25. P *trend* 0.04) DDSvegsub: $HR_{(T3VT1)}$ = 1.10 (95% CI: 0.62, 1.94. P *trend* 0.61) DDSveg: $HR_{(T3VT1)}$ = 1.09 (95% CI: 0.61, 1.24. P *trend* 0.86) DDSfruit: $HR_{(T3VT1)}$ = 1.33 (95% CI: 0.76, 2.33. P *trend* 0.32) *Eosophageal, GEJ ad cardia adenocarcinomas* DDSvegfr: $HR_{(T3VT1)}$ = 0.76 (95% CI: 0.43, 1.33. P *trend* 0.33) DDSvegsub: $HR_{(T3VT1)}$ = 1.17 (95% CI: 0.70, 2.92. P *trend* 0.62) DDSveg: $HR_{(T3VT1)}$ = 0.81 (95% CI: 0.48, 1.43. P *trend* 0.46) DDSfruit: $HR_{(T3VT1)}$ = 0.83 (95% CI: 0.46, 1.49. P *trend* 0.58) *Esophageal squamous cell carcinomas* DDSvegfr: $HR_{(T3VT1)}$ = 0.42 (95% CI: 0.17, 1.04. P *trend* 0.07) DDSvegsub: $HR_{(T3VT1)}$ = 0.59 (95% CI: 0.29, 1.22. P *trend* 0.26) DDSveg: $HR_{(T3VT1)}$ = 0.72 (95% CI: 0.32, 1.62. P *trend* 0.41) DDSfruit: $HR_{(T3VT1)}$ = 0.48 (95% CI: 0.21, 1.11. P *trend* 0.04)	Independent from quantity of consumption, more variety in vegetable and fruit consumption combined and fruit consumption alone may decrease risk of esophageal squamous cell carcinoma (significant results found in linear comparisons vs the categorical comparisons shown in the previous cell). No association for gastric or esophageal adenocarcinoma
22	Kenfield, S.A., et al., (2014) [55], Mediterranean diet and prostate cancer risk and mortality in the health professionals follow-up study	USA	Longitudinal cohort HPFS	N = 47,867 men aged 40–75 y at baseline in 1986	MMDS (1) categorised from least to highest adherence as follows: scores 0–3, 4–5 and 6–9 aMED (3) categorised into quintiles (Q1-Q5)	130-item FFQ completed at baseline and at subsequent regular intervals *Validated*	PCa incidence and mortality: Cox proportional hazards regression to estimate HR and 95% CI's for PCa incidence Adjusted for socio-demographic, health and health behaviour factors *F/U from baseline to 2010*	Median F/U = 23.2 y PCa incidence *MMDS* $HR_{(highest\ V\ lowest\ scores)}$ = 0.95 (95% CI: 0.90, 1.02, P *trend* 0.13) *aMED* $HR_{(Q5VQ1)}$ = 0.94 (95% CI: 0.86, 1.03, P *trend* 0.39)	A higher Mediterranean diet score was not associated with risk of advanced PCa or death from PCa

Table S2. *Cont.*

	Study	Location	Study Design & Cohort	Participants	Diet Quality Score	Method & Period of Dietary Analysis	Outcome Variable/s & Censoring	Cancer Incidence Results *	Overall Conclusions
23	Kyro, C., et al., (2013) [57], Adherence to a healthy Nordic food index is associated with a lower incidence of colorectal cancer in women: The Diet, Cancer and Health cohort study	Denmark	Longitudinal cohort Diet, Cancer and Health cohort	N = 57,053 men and women aged 50–60 y at baseline in 1993–7	Nordic food index Categorised into quintiles (Q1-Q5)	192-item FFQ at baseline. Measured intake over previous 12 mo *Validated*	Incidence rate ratios (IRR) for colorectal cancer and 95% CI's calculated from Cox proportional hazard models Adjusted for sociodemographic, health and health behaviour factors *F/U from baseline to 2009*	Median F/U = 13 y Men $IRR_{(Q5VQ1)}$ = 0.87 (95% CI: 0.61, 1.25. P *trend* 0.94) Women $IRR_{(Q5VQ1)}$ = 0.65 (95% CI: 0.46, 0.94. P *trend* 0.02	Higher adherence to the Nordic diet was associated with reduced risk of CRC in women
24	Li, W., et al., (2013) [61], Index-based dietary patterns and risk of esophageal and gastric cancer in a large cohort study	USA	Longitudinal cohort NIH-AARP Diet and Health Study	N = 494,698 men and women (aged 51–70 y at baseline [1995–96])	HEI-2005 (1) (categorised into quintiles, Q1-Q5) aMED (2) (5 categories: scores 0–2, 3, 4, 5–6, and 7–9)	124-item FFQ completed at baseline Diet ax period preceding 12 mo *Validated*	Multivariate adjusted HR and 95% CI's for risk of esophageal (esophageal squamous cell carcinoma [ESCC]; and esophageal adenocarcinoma [EAC]) and gastric cancer (cardia and non-cardia cancer) Adjusted for socio-demographic, health and health status factors *F/U from baseline to 2006*	μ F/U = 9.7 y HEI-2005 HR $ESCC_{(Q5\ Vs Q1)}$ = 0.51 (95% CI: 0.31, 0.86. P *trend* 0.001) HR $EAC_{(Q5\ Vs Q5)}$ = 0.75 (95% CI: 0.57, 0.98. P *trend* 0.01) HR gastric $cardia_{(Q5\ Vs Q5)}$ = 0.92 (95% CI: 0.67, 1.27. P *trend* 0.56) HR non-$cardia_{(Q5\ Vs Q5)}$ = 0.88 (95% CI: 0.65, 1.20. P *trend* 0.15) aMED HR $ESCC_{(highest\ Vs\ lowest\ scores)}$ = 0.44 (95% CI: 0.22, 0.88. P *trend* 0.03) HR $EAC_{(highest\ Vs\ lowest\ scores)}$ = 0.91 (95% CI: 0.66, 1.25. P *trend* 0.25) HR gastric $cardia_{(highest\ Vs\ lowest\ scores)}$ = 1.10 (0.76, 1.61. P *trend* 0.90) HR non-$cardia_{(highest\ Vs\ lowest\ scores)}$ = 0.75 (95% CI: 0.52, 1.09. P *trend* 0.11)	HEI-2005 and aMED scores are associated inversely with risk of esophageal cancers.
25	Li, W., et al., (2014a) [62], Index-based dietary patterns and risk of incident hepatocellular carcinoma and mortality from chronic liver disease in a prospective study	USA	Longitudinal cohort NIH-AARP Diet and Health Study	N = 494,942 men and women (aged 51–70 y at baseline [1995–96])	HEI-2010 (categorised into quintiles, Q1-Q5) aMED (2) (5 categories: scores 0–2, 3, 4, 5, and 6–9)	124-item FFQ completed at baseline Diet ax period preceding 12 mo Validated	Cox proportional hazards models used to calculate HR (95% CIs) and for incident primary hepatocellular carcinoma (HCC) Adjusted for socio-demographic, health and health status factors *F/U from baseline to 2006*	Up to 11 y F/U HEI-2010 HR $HCC_{(Q5\ V\ Q1)}$ = 0.72 (95% CI: 0.53, 0.97; P *trend* = 0.03) aMED HR $HCC_{(lowest\ Vs\ highest\ scores)}$ = 0.62 (95% CI: 0.47, 0.84; P *trend* = 0.0002	In this cohort higher HEI-2010 and aMED scores were associated with reduced risk of HCC

Table S2. *Cont.*

	Study	Location	Study Design & Cohort	Participants	Diet Quality Score	Method & Period of Dietary Analysis	Outcome Variable/s & Censoring	Cancer Incidence Results *	Overall Conclusions
26	Li, W., et al., (2014b) [20], Index-based dietary patterns and risk of head and neck cancer in a large prospective study	USA	Longitudinal cohort NIH-AARP Diet and Health Study	N = 494,967 men (μ age 62.1 ± 5.3 y) and women (μ age 61.9 ± 5.4 y)	HEI-2005 (1) (categorised into quintiles) aMED (2) (5 categories: scores 0–2, 3, 4, 5–6, and 7–9)	124-item FFQ completed at baseline (1995–6) Diet ax period preceding 12 mo Validated	Cox proportional hazards models used to calculate HR (95% CIs) and for incident head and neck cancer (cancers of the larynx, oral cavity and orohypopharynx) Adjusted for socio-demographic, health and health status factors *F/U from baseline to 2006*	Up to 11 y F/U HEI-2005 *Men:* HR $HNC_{(Q5\ V\ Q1)}$ = 0.74 (95% CI: 0.63, 0.89; P *trend* = 0.0008) *Women:* HR $HNC_{(Q5\ V\ Q1)}$ = 0.48 (95% CI: 0.33, 0.70; P *trend* < 0.0001) aMED *Men:* HR HNC(highest Vs lowest scores) = 0.80 (95% CI: 0.64, 1.01; P *trend* = 0.002) *Women:* HR HNC(highest Vs lowest scores) = 0.42 (95% CI: 0.24, 0.74. P *trend* < 0.0001)	Compliance with dietary guidelines may help reduce risk of incidence HNC
27	Mai, V., et al., (2005) [63], Diet quality and subsequent cancer incidence and mortality in a prospective cohort of women	USA	Longitudinal cohort BCDDP	N = 42,254 women (μ age 61 y)	RFS (1) (categorised into quantiles, Q1-Q4)	62-item FFQ (completed 1987–89) considers intake over previous 12 mo *Validated*	Cox proportional hazards models used to calculate HR (95% CIs) of total and site-specific cancer incidence and mortality Adjusted for health and health behaviour variables *F/U to 1998*	μ F/U = 9.5 y Cancer incidence Total cancer: $HR_{(Q4\ V\ Q1)}$ = 0.98 (95% CI: 0.88, 1.09; P *trend* 0.99) Breast cancer: $HR_{(Q4\ V\ Q1)}$ = 1.17 (95% CI: 1.01, 1.36; P *trend* 0.08) Lung cancer: $HR_{(Q4\ V\ Q1)}$ = 0.62 (95% CI: 0.46, 0.84; P *trend* < 0.001) Colorectal cancer: $HR_{(Q4\ V\ Q1)}$ = 0.94 (95% CI: 0.69, 1.27; P *trend* 0.56)	In this cohort the RFS was inversely associated with incidence lung cancer but not for other cancers
28	McCullough, M.L., et al., (2000a) [65], Adherence to the dietary guidelines for Americans and risk of major chronic disease in men	USA	Longitudinal cohort HPFS	N = 38,622 men (40–75 y at baseline in 1986)	HEI-f Categorised into quintiles (Q1-Q5)	131-item FFQ collected at baseline (1986–90) data collected on previous 12 mo intake *Validated*	Pooled logistic regression to calculate RR and 95% CI's for cancer risk Adjusted for health and health behaviour factors *F/U to 1994*	μ F/U = 8 y Cancer incidence $RR_{(Q5\ V\ Q1)}$ = 1.12 (95% CI: 0.95, 1.31; P *trend* 0.27)	No association between HEI-f score and cancer risk
29	McCullough, M.L., et al., (2000b) [66], Adherence to the dietary guidelines for Americans and risk of major chronic disease in women	USA	Longitudinal cohort NHS	N = 67,272 female nurses (30–55 y at baseline in 1976)	HEI-f Categorised into quintiles (Q1-Q5)	~130-item FFQ collected at baseline (1984), 1986 and 1990 collected on previous 12 mo intake *Validated*	Calculated RR as the incidence rate of disease among women in each quintile of HEI-f score divided by the incidence rate for women in the lowest quintile Adjusted for health and health behaviour factors *F/U to 1996*	Up to 12 y F/U Cancer incidence $RR_{(Q5\ V\ Q1)}$ = 1.02 (95% CI: 0.93, 1.121; P *trend* 0.578)	HEI-f in this cohort not associated with cancer risk

Table S2. *Cont.*

	Study	Location	Study Design & Cohort	Participants	Diet Quality Score	Method & Period of Dietary Analysis	Outcome Variable/s & Censoring	Cancer Incidence Results *	Overall Conclusions
30	McCullough, M.L., et al., (2002) [67], Diet quality and major chronic disease risk in men and women: moving toward improved dietary guidance	USA	Longitudinal cohort NHS HPFS	N = 38,615 men from HPFS (40–75 y at baseline in 1986) N = 67,271 women from NHS (30–55 y at baseline in 1984)	AHEI(1) RFS(2) Both scores categorised into quintiles (Q1-Q5)	NHS ~130-item FFQ collected at baseline (1984), 1986 and 1990 HPFS 131-item FFQ collected at baseline (1986–90) Both collected on previous 12 mo intake *Validated*	Calculated RR (95% CI's) as the incidence rate of total cancer in each quintile of AHEI and RFS score divided by the incidence rate in the lowest quintile Adjusted for health and health behaviour factors *F/U to 1996 for HPFS and 1994 for NHS*	Up to 8–12 y F/U HPFS *AHEI* $RR_{(Q5VQ1)}$ = 1.03 (95% CI: 0.87, 1.22; P *trend* 0.66) *RFS* $RR_{(Q5VQ1)}$ = 1.08 (95% CI: 0.94, 1.25; P *trend* 0.79) NHS *AHEI* $RR_{(Q5VQ1)}$ = 0.97 (95% CI: 0.88, 1.06; P *trend* 0.92) *RFS* $RR_{(Q5VQ1)}$ = 1.00 (95% CI: 0.85, 1.01; P *trend* 0.39)	Neither the AHEI nor RFS predicted cancer risk in this cohort of men and women
31	Miller, P.E., et al., (2013) [69], Comparison of 4 established DASH diet indexes: examining associations of index scores and colorectal cancer	USA	Longitudinal cohort NIH-AARP	N = 491,841 men and women (aged 50–71 at baseline in 1995–6)	DASH (Dixon) (2) Score categorised as follows: ≤1(referent category); 2, 3, ≥4 DASH (Mellen) (3) DASH (Fung) (1) DASH (Güenther) (4) Scores categorised into quintiles (Q1-Q5)	124-item FFQ data collected on intake over previous 12 mo *Validated*	Cox proportional hazard ratios and 95% CI's for colorectal cancer incidence risk Adjusted for socio-demographic, health and health behaviour factors *F/U to 2006*	Up to 11 y F/U Men HR $Dixon_{(highest\ V\ lowest\ scores)}$ = 0.77 (95% CI: 0.69, 0.87) HR $Mellen_{(Q5VQ1)}$ = 0.78 (95% CI: 0.71, 0.86) HR $Fung_{(Q5VQ1)}$ = 0.75 (95% CI: 0.68, 0.83) HR $Güenther_{(Q5VQ1)}$ = 0.81 (95% CI: 0.74, 0.90) Women HR $Dixon_{(highest\ V\ lowest\ scores)}$ = 1.01 (95% CI: 0.80, 1.28) HR $Mellen_{(Q5VQ1)}$ = 0.79 (95% CI: 0.68, 0.91) HR $Fung_{(Q5VQ1)}$ = 0.84 (95% CI: 0.73, 0.96) HR $Güenther_{(Q5VQ1)}$ = 0.84 (95% CI: 0.73, 0.97)	The 4 established DASH index scores showed a significant inverse association with colorectal cancer risk among both men (all 4 scores) and women (all but the Dixon DASH score)

Table S2. *Cont.*

	Study	Location	Study Design & Cohort	Participants	Diet Quality Score	Method & Period of Dietary Analysis	Outcome Variable/s & Censoring	Cancer Incidence Results *	Overall Conclusions
32	Nilsson, L.M., et al., (2013) [75], Low-carbohydrate, high-protein diet score and risk of incident cancer: a prospective cohort study	Västerbotten, Sweden	Longitudinal cohort VIP	N = 62,582 men and women (ongoing recruitment commenced 1990)	LCHP score (3) (categorised into low [2–8 pts]; medium [9–13 pts; and high [14–20 pts] scores])	3 FFQs One older and one newer 84-item FFQ and a more recent 65-item version *Validated*	Cox proportional hazard ratios for total and site-specific cancer incidence risk Adjusted for socio-demographic, health and health behaviour factors *F/U to 2007*	Median F/U = 9.7 y (range = 1 d–17.9 y) Men *Total cancer* $HR_{(high\ V\ low\ scores)}$ = 0.97 (95% CI: 0.97, 1.03; P *trend* 0.973) *Prostate cancer* $HR_{(high\ V\ low\ scores)}$ = 0.97 (95% CI: 0.85, 1.15; P *trend* 0.777) *Colorectal cancer* $HR_{(high\ V\ low\ scores)}$ = 1.00 (95% CI: 0.66, 1.52; P *trend* 0.511) *Respiratory tract cancer* $HR_{(high\ V\ low\ scores)}$ = 1.24 (95% CI: 0.62, 2.47; P *trend* 0.381) Women *Total cancer* $HR_{(high\ V\ low\ scores)}$ = 1.00 (95% CI: 0.85, 1.15; P *trend* 0.777) *Breast cancer* $HR_{(high\ V\ low\ scores)}$ = 1.00 (95% CI: 0.79, 1.27; P *trend* 0.924) *Colorectal cancer* $HR_{(high\ V\ low\ scores)}$ = 0.83 (95% CI: 0.52, 1.34; P *trend* 0.459) *Respiratory tract cancer* $HR_{(high\ V\ low\ scores)}$ = 1.37 (95% CI: 0.67, 2.82; P *trend* 0.328)	No association between cancer risk and LCHP diet in this cohort
33	Reedy, J., et al., (2008) [13], Index-based dietary patterns and risk of colorectal cancer: the NIH-AARP Diet and Health Study	USA	Longitudinal cohort NIH-AARP	N = 492,382 men and women (aged 50–71 at baseline in 1995–6)	HEI-2005 (1) AHEI (1) MMDS (2) RFS (1) All scores categorised by quintile	124-item FFQ completed at baseline Diet ax period preceding 12 mo *Validated*	Cox proportional hazard ratios for colorectal cancer incidence risk Adjusted for socio-demographic, health and health behaviour factors *F/U to 2000*	Up to 5 y F/U Men HEI-2005: $HR_{(Q5VQ1)}$ = 0.72 (95% CI: 0.62, 0.83) * AHEI: $HR_{(Q5VQ1)}$ = 0.71 (95% CI: 0.61, 0.82) * MDS: $HR_{(Q5VQ1)}$ = 0.72 (95% CI: 0.63, 0.83) * RFS: $HR_{(Q5VQ1)}$ = 0.75 (95% CI: 0.65, 0.87) * Women HEI-2005: $HR_{(Q5VQ1)}$ = 0.80 (95% CI: 0.64, 0.98) * AHEI: $HR_{(Q5VQ1)}$ = 0.83 (95% CI: 0.66, 1.05) MDS: $HR_{(Q5VQ1)}$ = 0.89 (95% CI: 0.72, 1.11) RFS: $HR_{(Q5VQ1)}$ = 1.01 (95% CI: 0.80, 1.28)	All four scores associated with a reduced risk colorectal cancer in men. HEI-2005 associated with reduced colorectal cancer risk in women

Table S2. *Cont.*

	Study	Location	Study Design & Cohort	Participants	Diet Quality Score	Method & Period of Dietary Analysis	Outcome Variable/s & Censoring	Cancer Incidence Results *	Overall Conclusions
34	Trichopoulou, A., et al., (2010) [80], Conformity to traditional Mediterranean diet and breast cancer risk in the Greek EPIC (European Investigation into Caner and Nutrition) cohort	Greece	Longitudinal cohort EPIC	N = 14,807 women aged 20–86 at baseline in 1994–8	MMDS (1) Categorised as follows: 0–3, 4–5, 6–9)	150-item FFQ Completed at baseline Diet ax period preceding 12 mo *Validated*	Cox proportional hazard ratios for breast cancer incidence risk Adjusted for socio-demographic, health and health behaviour factors *F/U to 2009*	μ F/U = 9.8 y (range: 23 d-15.8 y) *All women* $HR_{(highest\ Vs\ lowest\ score)}$ = 0.84 (95% CI: 0.59, 1.20; P *trend* 0.12) *Pre-menopausal* $HR_{(highest\ Vs\ lowest\ score)}$ = 1.13 (95% CI: 0.69, 1.85; P *trend* 0.91) *Post-menopausal* $HR_{(highest\ Vs\ lowest\ score)}$ = 0.59 (95% CI: 0.34, 1.03; P *trend* 0.03)	Conformity to the Mediterranean diet may be protective of breast cancer risk for post-menopausal women
35	Von Rueston, A., et al., (2010) [83], Adherence to recommendation of the German food pyramid and risk of chronic diseases: results from the EPIC-Potsdam study	Potsdam, Germany	Longitudinal cohort EPIC-Potsdam	N = 25,531 men and women aged 35–65 y at baseline in 1994–8	GFPI Categorised into quintiles (Q1-Q5)	148-item FFQ based on intake over previous 12 mo collected dietary data at baseline *Validated*	Cox proportional hazard ratios for cancer incidence risk Adjusted for socio-demographic, health and health behaviour factors *F/U to 2007*	μ F/U = 7.8 y Cancer risk *Men* $HR_{(Q5VQ1)}$ = 1.16 (95% CI: 0.83, 1.62; P *trend* 0.4015) *Women* $HR_{(Q5VQ1)}$ = 0.79 (95% CI: 0.58, 1.08; P *trend* 0.1444)	Adherence to the GFPI does not have much of an impact on overall or cancer mortality risk

* Where a study provided several risk estimates with different degrees of adjustment for confounding the estimate presented is the one adjusting for the largest number of factors.

Table S3. Cancer mortality and diet quality score evidence table. F/U: Follow-up.

	Study	Location	Study Design & Cohort	Participants	Diet Quality Score	Method & Period of Dietary Analysis	Outcome Variable/s	Cancer Mortality Results *	Overall Conclusions
1	Akbaraly, T.N., et al. (2011) [28]. Alternative Healthy Eating Index and mortality over 18 y of follow-up: results from the Whitehall II cohort. (8)	Brittan, London	Cohort Whitehall II cohort	7319 men and women. μ age 49.5 yr (range 39–63 yrs) Participants all office-based staff from 20 civil service departments	AHEI (1) (2.5–87.5)	Semi-quantitative FFQ completed 1991–3	Multivariate Cox regression used to model association between diet quality and mortality (HR and 95% CIs) Adjusted for sociodemographic factors, health behaviours and health factors. *F/U to 2010*	Up to 18 yr F/U Cancer mortality: $HR_{(highest\ tertile\ AHEI\ vs\ lowest\ tertile)}$: 0.80 (95% CI: 0.58, 1.11)	No association between cancer mortality and AHEI Association found for CVD-related mortality
2	Buckland, G., et al., (2011) [36], Adherence to the Mediterranean diet reduces mortality in the Spanish cohort of the European Prospective Investigation into Cancer and Nutrition (EPIC-Spain)	Spain	Longitudinal cohort EPIC—Spain	*N* = 40,622 men and women aged 29–60 y (μ = 49.3±8 y) at baseline in 1992–6	rMED (2) Score categorised into 3 quintiles (Q1-Q3): High (scores 11–18); Medium (scores 7–10); Low (scores 0–6)	FFQ measured intake over previous 12 mo *Validated*	Multivariate Cox regression used to model association between diet quality and mortality (HR and 95% CIs) Adjusted for socio-demographic, health and health behaviour factors *F/U to 2006*	μ F/U = 13.4 y Cancer mortality $HR_{(Q3VQ1)}$ = 0.91 (95% CI: 0.75, 1.12. P *trend* 0.414)	Higher adherence to the rMED dietary pattern associated with reduced all-cause mortality as well as CVD mortality but not cancer mortality

Table S3. *Cont.*

	Study	Location	Study Design & Cohort	Participants	Diet Quality Score	Method & Period of Dietary Analysis	Outcome Variable/s	Cancer Mortality Results *	Overall Conclusions
3	Drake, I., et al., (2012) [43], Scoring models of a diet quality index and the predictive capability of mortality on a population-based cohort of Swedish men and women	Malmö, Sweden	Longitudinal cohort Malmö Diet and Cancer cohort	*N* = 6940 men (μ(sd) age = 59.2 y(7.1 y)) *N* = 10,186 women (μ(sd) age = 57.5(8.0)) Recruited from 1992–6	DQI-SNR Model 1: scoring based on pre-defined cut-offs from the Swedish Nutrient Recommendations and Dietary Guidelines (score range: 0–6) Model 2: based on study-population's energy-adjusted intakes as cut-offs (score range: 0–6) Model 3: ranked individuals into quintiles of energy-adjusted intake. A proportional score (0–5) assigned to the quintile groups for each component (score range 6–30)	7 d FR & 168-item FFQ	Multivariate Cox regression used to model association between diet quality and mortality Adjusted for socio-demographic factors, health behaviours and health factors. *F/U to 2008*	μ F/U = 14.2 y *Cancer mortality risk:* Model 1 Men: $HR_{(tertile\ 3\ V\ tertile\ 1)}$ = 0.82 (95% CI: 0.68, 0.97) Women: $HR_{(tertile\ 3\ V\ tertile\ 1)}$ = 0.91 (95% CI: 0.70, 1.17) Model 2 Men $HR_{(tertile\ 3\ V\ tertile\ 1)}$ = 0.99 (95% CI: 0.75, 1.28) Women: $HR_{(tertile\ 3\ V\ tertile\ 1)}$ = 1.02 (95% CI: 0.77, 1.36) Model 3 Men: $HR_{(tertile\ 3\ V\ tertile\ 1)}$ = 0.90 (95% CI: 0.73, 1.10) Women: $HR_{(tertile\ 3\ V\ tertile\ 1)}$ = 0.90 (95% CI: 0.73, 1.12)	A dietary pattern similar to that outlined in the SNRs and SDGs is associated with reduced risk of all-cause and CVD mortality but not cancer mortality.

Table S3. *Cont.*

	Study	Location	Study Design & Cohort	Participants	Diet Quality Score	Method & Period of Dietary Analysis	Outcome Variable/s	Cancer Mortality Results *	Overall Conclusions
4	Cuenca-Garcia, M., et al., (2014) [42], Dietary indices, cardiovascular risk factors and mortality in middle-aged adults: findings from the Aerobics Centre Longitudinal Study (9)	Cooper Clinic, Dallas, Texas USA	Cohort ACLS	*N* = 12,449 men (77%) and women (μ age = 46.9 y)	Ideal Diet Index (IDI) (0–8) Mediterranean Diet Score (MMDS [6]) (0–9) Diet Quality Index (DQI [2]) (0–16) All scores categorised into quartiles (Q1-Q4)	3 d FR completed at baseline (1987–1999)	Cox proportional hazards regression for HR and associated 95% CI's for total and cause-specific mortality Adjusted for socio-demographic, health behaviour, and factors of health status *F/U to 2003*	μ F/U = 11.6 y *For cancer mortality:* IDI: $HR_{(Q4VsQ1)}$ = 1.06, 95% CI (0.61, 1.86. P *trend* 0.913) DQI: $HR_{(Q4VsQ1)}$ = 1.26, 95% CI (0.72, 2.22. P *trend* 0.458) MMDS: $HR_{(Q4VsQ1)}$ = 1.63, 95% CI (0.91, 2.92. P *trend* 0.432)	No association with any diet quality index tested in this study and cancer mortality
5	Fung, T.T., et al., (2010) [46], Low-carbohydrate diets and all-cause and cause-specific mortality: two cohort studies	USA	Longitudinal cohort NHS HPFS	*N* = 85,168 women aged 34–55 y at baseline in 1980 *N* = 44,548 men aged 40–75 y at baseline in 1986	LCHP (2) LCHP-V LCHP-AB For each score participants categorised into deciles (D1-D10)	FFQ self-administered at baseline for both cohorts *Validated* for both cohorts	Cox proportional hazards regression for HR and associated 95% CI's for total and cause-specific mortality Adjusted for health and health behaviour factors *F/U to 2003*	Up to 23 y F/U *Total cancer mortality:* *NHS* Up to F/U of 26 y *LCHP* $HR_{(D10VD1)}$ = 1.19 (95% CI: 0.99, 1.42. P *trend* 0.128) *LCHP-V* $HR_{(D10VD1)}$ = 0.96 (95% CI: 0.87, 1.05. P *trend* 0.23) *LCHP-AB* $HR_{(D10VD1)}$ = 1.28 (95% CI: 1.02, 1.60. P *trend* 0.089) *HPFS* Up to F/U of 20 y *LCHP* $HR_{(D10VD1)}$ = 1.32 (95% CI: 1.11, 1.57. P *trend* < 0.001) *LCHP-V* $HR_{(D10VD1)}$ = 1.00 (95% CI: 0.84, 1.18. P *trend* 0.35) *LCHP-AB* $HR_{(D10VD1)}$ = 1.45 (95% CI: 1.23, 1.72. P *trend* < 0.001)	A higher total LCHP and, in particular, a high LCHP-A diet score was associated with increased risk of all-sites cancer mortality in men. This association was weaker in women and did not reach statistical significance. In pooled analysis an association observed for LCHP scores and colorectal cancer risk ($HR_{(D10VD1)}$ = 1.45 (95% CI: 1.06, 1.62. P *trend* 0.048) None of the LCHP scores associated with breast or prostate cancer death.

Table S3. *Cont.*

	Study	Location	Study Design & Cohort	Participants	Diet Quality Score	Method & Period of Dietary Analysis	Outcome Variable/s	Cancer Mortality Results *	Overall Conclusions
6	Huijbregts, P., et al., (1997) [47], Dietary pattern and 20 year mortality in elderly men in Finland, Italy and the Netherlands: longitudinal cohort study	Finland, Italy and the Netherlands	Longitudinal cohort Seven Countries Study	*N* = 3045 men aged 50–70 yrs at baseline in 1970	Healthy diet indicator (HDI [1]) Participants divided into thirds based on HDI scores (<2; 2; and >2) Finland & the Netherlands (<3; 3–4; >4) Italy	Cross-check diet hx	Cox proportional hazards regression for RR and associated 95% CI's for total and cause-specific mortality Adjusted for socio-demographic, health behaviour, and factors of health status *F/U to 1990*	Up to 20 y F/U Pooled analysis (including all participants from each centre) Cancer Mortality $RR_{(highest\ V\ lowest\ HDI\ scores)}$ = 0.85 (P *trend* 0.13)	There is an association between HDI and risk of cancer death. But this is not significant. There is a significant association between total mortality and risk of death
7	Kappeler, R., et al., (2013) [54], Meat consumption and diet quality and mortality in NHANES III	USA	Cross-sectional NHANES III	*N* = 17,611 men and women recruited in 1988–94	HEI Categorised into tertiles (T1-T3)	24 h recall completed at bassline	Cox proportional hazards regression for HR and associated 95% CI's for total and cause-specific mortality Adjusted for socio-demographic, health and health behaviour factors *F/U to 2010*	Up to 22 yr F/U *Cancer mortality* Men $HR_{(T3VT1)}$ = 0.74 (95% CI: 0.46, 1.17, P *trend* 0.10) Women $HR_{(T3VT1)}$ = 0.94 (95% CI: 0.46, 1.95, P *trend* 0.82) Pooled $HR_{(T3VT1)}$ = 0.75 (95% CI: 0.51, 1.11, P *trend* 0.14)	No significant associations between HEI and cancer mortality
8	Kant, A.K., et al., (1995) [51], Dietary diversity and subsequent cause-specific mortality in the NHANES I epidemiologic follow-up	USA	Longitudinal cohort NHANES Epidemiologi c Follow-Up Study	*N* = 10,337 men and women aged 25–74 y at baseline in 1971–75	DDS (4) Categorised into the following groups: scores 0–2, 3, 4, and 5	24 h recall completed at baseline	Cox proportional hazards regression for RR and associated 95% CI's for total and cause-specific mortality Adjusted for socio-demographic, health and health behaviour factors *F/U to 1987*	Median F/U = 14.2 y Cancer mortality *Men* $RR_{(scores\ 0\text{–}2\ Vs\ 5)}$ = 1.3 (95% CI: 0.8, 2.1. P *trend* 0.52) *Women* $RR_{(scores\ 0\text{–}2\ Vs\ 5)}$ = 1.4 (95% CI: 0.8, 2.3. P *trend* 0.87)	Results of the study are suggestive of an increased risk of cancer mortality associated with diets characterised by the omission of several major food groups
9	Kant, A.K., et al., (2000) [52], A prospective study of diet quality and mortality in women	USA	Cohort BCDDP	*N* = 42,254 women enrolled in the BCDDP at baseline (1987–89) µ age 61.7 yrs	RFS (1) Categorised into quartiles (Q1-Q4)	FFQ at baseline *Validated*	Cox proportional hazards regression for RR and 95% CI's all cause and cause-specific mortality, including cancer mortality Adjusted for socio-demographic, health and health behaviour factors *F/U to 1993*	Median F/U = 5.6 y *Risk of cancer mortality:* $RR_{(Q4VQ1)}$ = 0.60 (95% CI: 0.49, 0.74. P *trend* < 0.001)	Study suggests that women reporting a diet higher in fruits, vegetables, who grains, low-fat dairy, and lean meats have a 40% reduced risk of cancer death.

Table S3. *Cont.*

	Study	Location	Study Design & Cohort	Participants	Diet Quality Score	Method & Period of Dietary Analysis	Outcome Variable/s	Cancer Mortality Results *	Overall Conclusions
10	Kant, A.K., et al., (2009) [53], Patterns of recommended dietary behaviours predict subsequent risk of mortality in a large cohort of men and women in the united states (8)	USA	Cohort NIH-American Association of Retired Persons cohort	*N* = 350,886 men and women (aged 50–71 yrs at baseline in 1995–1996)	Dietary behaviour score (DBS) (0–36) Categorised into quintiles (Q1-Q5)	FFQ at baseline and then measured at 10 year follow-up	Cox proportional hazards regression for RR and 95% CI's all cause and cause-specific mortality, including cancer mortality Adjusted for socio-demographic, health and health behaviour factors *F/U to 2006*	Up to 10.5 y F/U *Men* All sites cancer $RR_{(Q5\ vs\ Q1)}$ = 0.79 95% CI: 0.73, 0.86. *p* < 0.0001 * *Women* All sites cancer $RR_{(Q5\ vs\ Q1)}$ = 0.81 95% CI: 0.73, 0.90. *p* < 0.0001 *	Adoption of recommended dietary behaviours was associated with lower total and cancer mortality
11	Kaluza, J., et al., (2009) [50], Diet quality and mortality: a population-based prospective study of men	Sweden	Longitudinal cohort Cohort of Swedish Men	*N* = 40, 837 aged 45–79 y at baseline in 1997–8	RFS (3) Categorised into low, medium and high groups as follows: ≤ 20 points, 21–27 points; and 28 points Non-RFS (1) categorised into low, medium and high groups as follows: ≤ 2 points, 3–4 points, ≥ points	96-item FFQ administered Measured intake over preceding 12 mo	Cox proportional hazards regression for HR all cause and cause-specific mortality, including cancer mortality Adjusted for socio-demographic, health and health behaviour variables *F/U to 2005*	μ F/U = 7.7 y *Cancer mortality:* *RFS* $HR_{(high\ V\ low\ scores)}$ = 1.09 (95% CI: 0.84, 1.41. P *trend* 0.28) *Non-RFS* $HR_{(high\ V\ low\ scores)}$ = 1.17 (95% CI: 0.94, 1.46. P *trend* 0.49)	No significant associations with cancer mortality were observed for RFS of Non-RFS
12	Kenfield, S.A., et al., (2014) [55], Mediterranean diet and prostate cancer risk and mortality in the health professionals follow-up study	USA	Longitudinal cohort HPFS	*N* = 47,867 men aged 40–75 y at baseline in 1986	MMDS (1) categorised from least to highest adherence as follows: scores 0–3, 4–5 and 6–9 aMED (3) categorised into quintiles (Q1-Q5)	130-item FFQ completed at baseline and at subsequent regular intervals *Validated*	PCa incidence and mortality: Cox proportional hazards regression to estimate HR and 95% Cis Adjusted for socio-demographic, health and health behaviour factors *F/U from baseline to 2010*	Median F/U = 23.2 y PCa mortality *aMED* $HR_{(Q5VQ1)}$ = 0.94 (95% CI: 0.86, 1.03, P *trend* 0.39) *MMDS* $HR_{(highest\ V\ lowest\ scores)}$ = 1.01 (95% CI: 0.75, 1.38, P *trend* 0.95) *aMED* $HR_{(Q5VQ1)}$ = 1.14 (95% CI: 0.73, 1.76, P *trend* 0.83)	A higher Mediterranean diet score was not associated with risk of advanced PCa or death from PCa

Table S3. *Cont.*

	Study	Location	Study Design & Cohort	Participants	Diet Quality Score	Method & Period of Dietary Analysis	Outcome Variable/s	Cancer Mortality Results *	Overall Conclusions
13	Knoops, K.T.B., et al., (2004) [56], Mediterranean Diet, lifestyle factors, and 10-year mortality in elderly European men and women	Belgium, France, Greece, Hungary, Italy, the Netherlands, Portugal, Spain, Switzerland	Longitudinal cohort HALE (European cohort) Survey in Europe on Nutrition and the Elderly: a concerned Action (SENECA) Finland, Italy the Netherlands elderly (FINE) study	*N* = 1507 healthy men aged 70–90 y at baseline in 1988 *N* = 832 women aged 70–90 at baseline in 1988	MMDS (5) 0 (low quality)—8 (high quality)	SENECA Diet hx collected dietary data from the month prior to interview FINE Diet hx collected dietary data from 2–4 wks prior to interview	Total and cause-specific mortality: Cox proportional hazards regression and 95% Cis Adjusted for socio-demographic, health and health behaviour factors *F/U from baseline to 2000*	μ F/U = 10 y (range: 8.9–10.5 y) *Cancer mortality* $RR_{(\geq 4 \text{ points on MMDS V} < 4 \text{ points})}$ = 0.90 (95% CI: 0.70, 1.17)	No association between MMDS and cancer mortality in this cohort
14	Lagiou, P., et al., (2006) [58], Mediterranean dietary pattern and mortality among young women: a cohort study in Sweden	Sweden	Cohort, longitudinal Scandinavian Women's Lifestyle and Health Cohort	*N* = 42,237 women aged 30–40 y at baseline in 1991–2	MMDS (1) Categorised into three tertiles (scores 0–3, 4–5, and 6–9 for T1-T3)	80-item FFQ administered at baseline collected data on intake over preceding 6 mo *Validated*	Cox proportional hazards regression for HR and 95% CI's for total and cause-specific mortality Adjusted for socio-demographic, health and health behaviour factors *F/U from baseline to 2003*	μ F/U = 12 y Cancer mortality All women $HR_{(\text{tertile 3 V tertile 1})}$ = 0.80 (95% CI: 0.57, 1.13) Women aged <40 at enrolment $HR_{(\text{tertile 3 V tertile 1})}$ = 1.07 (95% CI: 0.79, 1.43) Women aged ≥40 y at enrolment $HR_{(\text{tertile 3 V tertile 1})}$ = 0.84 (95% CI: 0.71, 1.01)	For women <40 y old at enrolment, no association between Mediterranean diet and cancer mortality. For women 40–49 y at enrolment link found in the model only adjusted for age ($HR_{(\text{tertile 3 V tertile 1})}$ = 0.78 (95% CI: 0.66, 0.93).
15	Lagiou, P., et al., (2007) [59], Low carbohydrate-high protein diet and mortality in a cohort of Swedish women	Sweden	Cohort, longitudinal Scandinavian Women's Lifestyle and Health Cohort	*N* = 42,237 women aged 30–40 y at baseline in 1991–2	LCHP (3) (2–10 points)	80-item FFQ administered at baseline collected data on intake over preceding 6 mo *Validated*	Cox proportional hazards regression for HR total and cause-specific mortality Adjusted for socio-demographic, health and health behaviour factors *F/U from baseline to 2003*	μ F/U = 12 y Cancer mortality *All women* $HR_{(\text{per 2 unit increase in LCHP score})}$ = 1.02 (95% CI: 0.96, 1.08) *Women ≤39 y* $HR_{(\text{per 2 unit increase in LCHP score})}$ = 1.04 (95% CI: 0.92, 1.15) *Women 40–49 y* $HR_{(\text{per 2 unit increase in LCHP score})}$ = 1.02 (95% CI: 0.94, 1.08)	No association between LCHP diet and cancer mortality

Table S3. *Cont.*

	Study	Location	Study Design & Cohort	Participants	Diet Quality Score	Method & Period of Dietary Analysis	Outcome Variable/s	Cancer Mortality Results *	Overall Conclusions
16	Lee, M, et al., (2011) [60], A simple food quality index predicts mortality in elderly Taiwanese	Taiwan	Cohort The Elderly Nutrition and Health Survey	*N* = 1743 men and women	ODI-R (categories: <50, 50–55, 55–60, 60–65, 65–70, >70) DDS (3) (categories <3, 4, 5, 6)	24 h recall and 18 item FFQ completed at baseline 1999–2000	Cox proportional hazards regression for HR and 95% CI's for total and cause-specific mortality risk (including total cancer risk) Adjusted for socio-demographic, health, and health behaviour variables *F/U from baseline to 2008*	Up to 9 y F/U For cancer risk: *ODI-R* HR(highest compared to lowest scores) = 0.48 (95% CI: 0.25, 0.93, P *trend* 0.14) *DDS* HR(highest compared to lowest scores) = 0.46 (95% CI: 0.20, 1.07, P *trend* 0.03)	Of the two, DDS is more predictive of overall mortality risk. The DDS did show a possible inverse relationship with cancer mortality risk
17	Mai, V., et al., (2005) [63], Diet quality and subsequent cancer incidence and mortality in a prospective cohort of women	USA	Longitudinal cohort BCDDP	*N* = 42,254 women (μ age 61 y)	RFS (1) Categorised into quantiles (Q1-Q5)	62-item FFQ (completed 1987–89) considers intake over previous yr *Validated*	Cox proportional hazards models used to calculate HR (95% CIs) of total and site-specific cancer incidence and mortality Adjusted for socio-demographic, health, and health behaviour variables *F/U to 1998*	μ F/U = 9.5 y Cancer mortality *Total cancer:* $HR_{(Q4\ v\ Q1)}$ = 0.74 (95% CI: 0.63, 0.86; P *trend* < 0.001) *Breast cancer:* $HR_{(Q4\ v\ Q1)}$ = 0.75 (95% CI: 0.56, 1.00; P *trend* 0.06) *Lung cancer*: $HR_{(Q4\ v\ Q1)}$ = 0.54 (95% CI: 0.29, 0.84; P *trend* < 0.001) * *Colorectal cancer:* $HR_{(Q4\ v\ Q1)}$ = 0.49 (95% CI: 0.29, 0.86; P *trend* < 0.01) * *Other cancers*: $HR_{(Q4\ v\ Q1)}$ = 0.85 (95% CI: 0.67, 1.08; P *trend* 0.11)	In this cohort, the RFS was inversely associated with overall, lung and colorectal cancer mortality. Borderline significance was seen for breast cancer mortality
18	Martinez-Gonzalez, M.A., (2012) [64] The Mediterranean diet is associated with a reduction in premature mortality among middle-aged adults	Spain	Seguimiento Universidad de Navarra (SUN) Project	*N* = 15,535 university students (μ age 38.1 ± 11.8 y)	MMDS (1) (categorised into 3 groups: low [0–2 pts]; moderate [3–5 pts]; and high [6–9 pts])	136-item FFQ completed from 1999–2009 *Validated*	Cox proportional hazards models used to calculate HR (95% CIs) for cancer mortality Adjusted for socio-demographic, health and health behaviour factors *F/U to 2010*	From 2–10 y F/U For a two point increment in MMDS, the HR death from cancer = 1.03 (95% CI: 0.73, 1.45; P *trend* = 0.80)	Adherence to MMDS associated with reduced risk of total mortality in this young cohort No association between MMDS and death from cancer in this young cohort
19	Michels, K.B., & Wolk A. (2002) [68], A prospective study of variety of healthy foods and mortality in women	Sweden	Longitudinal cohort Mammography Screening Cohort	*N* = 59,038 women	RFS (4) Non-RFS (2) Categorised into quintiles (Q1-Q5)	60-item FFQ completed at baseline (1987–9) and assessed intake over previous 6 mo *Validated*	Cox proportional hazards models used to calculate HR (95% CIs) for overall and cause-specific mortality Adjusted for socio-demographic, health and health status variables *F/U to 1998*	μ F/U = 9.9 y *Cancer mortality risk:* RFS $HR_{(Q5VQ1)}$ = 0.76 (95% CI: 0.60, 0.96. P *trend* 0.005) Non-RFS $HR_{(Q5VQ1)}$ = 1.52 (95% CI: 1.13, 2.05. P *trend* 0.02)	The RFS and non-RFS are both associated with risk of cancer mortality in this cohort

Table S3. *Cont.*

	Study	Location	Study Design & Cohort	Participants	Diet Quality Score	Method & Period of Dietary Analysis	Outcome Variable/s	Cancer Mortality Results *	Overall Conclusions
20	Mitrou, P.N., et al., (2007) [70], Mediterranean dietary pattern and prediction of all-cause mortality in a US population	USA	Longitudinal cohort NIH-AARP	*N* = 214,284 men *N* = 166,012 women	tMED aMED(2) Both scores categorised into 3 groups: scores 0–3, 4–5, and 6–9	124-item FFQ completed baseline 1995 *Validated*	Cox proportional hazards models used to calculate HR (95% CIs) for cancer mortality Adjusted for socio-demographic, health and health status variables *F/U to 2005*	10 y F/U *Cancer mortality risk:* Men tMED: $HR_{(highest\ V\ lowest\ scores)}$ = 0.79 (95% CI: 0.72, 0.87) aMED: $HR_{(highest\ V\ lowest\ scores)}$ = 0.83 (95% CI: 0.76, 0.91; P *trend* < 0.001) Women tMED: $HR_{(highest\ V\ lowest\ scores)}$ = 0.89 (95% CI: 0.79, 1.01) aMED: $HR_{(highest\ V\ lowest\ scores)}$ = 0.88 (95% CI: 0.78, 1,00; P *trend* 0.04)	Results provide strong evidence for a beneficial effect of higher conformity with the Mediterranean diet and cancer mortality
21	Mursu, J., et al., (2013) [71], Diet quality indexes and mortality in postmenopausal women: the Iowa Women's Health Study	Iowa, USA	Longitudinal cohort Iowa Women's Health Study	*N* = 29,634 women (μ age 61.4 ± 4.2 y)	AHEI-2010 A priori diet quality score Both scores categorised into quartiles (Q1-Q4)	127-item FFQ. Data collected at baseline in 1986 and at F/U (2004) *Validated*	Cox proportional hazards models used to calculate RR (95% CIs) for cancer mortality Adjusted for socio-demographic, health and health status variables *F/U to 2008*	μ F/U = 20.3 y Using 1986 dietary data N = 29,634 *AHEI-2010* $RR_{(Q4VQ1)}$ = 0.88 (95% CI: 0.79, 0.98; P *trend* < 0.001) *A priori score* $RR_{(Q4VQ1)}$ = 0.86 (95% CI: 0.77, 0.95; P *trend* 0.025) Using 2004 data for remaining N = 15,076 *AHEI-2010* $RR_{(Q4VQ1)}$ = 0.83 (95% CI: 0.63, 1.09; P *trend* 0.037) *A priori score* $RR_{(Q4VQ1)}$ = 0.70 (95% CI: 0.52, 0.94; P *trend* 0.028) *	Both diet quality indices in this study predicted mortality from cancer
22	Nakamura, Y., et al., (2009) [72], A Japanese diet and 19-year mortality: national integrated project for prospective observation of non-communicable diseases and its trends in the aged, 1980	Japan	Longitudinal cohort National Integrated Project for Prospective Observation of Non-Communicable Diseases and its Trends in the Aged, 1980	*N* = 9086 (aged ≥30 y at baseline in 1980)	Reduced-salt Japanese diet score (categorised into tertiles T1-T3: scores 0–2, 3 and 4–7)	31-item FFQ (administered at baseline, 1980)	Cox proportional hazards models used to calculate HR (95% CIs) of cancer mortality Adjusted for socio-demographic, health and health status variables *F/U to 1999*	Up to 19 y F/U Cancer mortality $HR_{(T3VT1)}$ = 0.85 (95% CI: 0.69, 1.05; P *trend* 0.13)	Adherence to a healthy Japanese diet is associated with reduced all-cause and CVD mortality. No association noted for cancer mortality

Table S3. *Cont.*

	Study	Location	Study Design & Cohort	Participants	Diet Quality Score	Method & Period of Dietary Analysis	Outcome Variable/s	Cancer Mortality Results *	Overall Conclusions
23	Nilsson, L.M., et al., (2012a) [73], A traditional Sami diet score as a determinant of mortality in a general northern Swedish population	Västerbotten, Sweden	Longitudinal cohort VIP	*N* = 77,319 men and women recruited from 1990–2008	Traditional Sami Diet Score (categorised into groups: low [scores 0–3]; medium [scores 4–5]; high [scores 6–8])	3 FFQs One older and one newer 84-item FFQ and a more recent 65-item version	Cox proportional hazards models used to calculate HR (95% CIs) of cancer mortality Adjusted for socio-demographic, health, and health behaviour factors *F/U to 2007*	1 d–19 y F/U For every one point increase in traditional Sami diet score HR for cancer mortality risk = 1.05 (95% CI: 0.99, 1.10; P *tend* 0.102) for men. For women HR = 1.03 (95% CI: 0.97, 1.09; P *tend* 0.304)	No identified association between the traditional Sami diet score and cancer mortality
24	Nilsson, L.M., et al., (2012b) [74], Low-carbohydrate, high-protein score and mortality in a northern Swedish population-based cohort	Västerbotten, Sweden	Longitudinal cohort VIP	*N* = 37,639 men *N* = 39,680 women recruited from 1990–2008 (median age at recruitment 49 y)	LCHP score (3) (categorised into groups; low [scores 2–8]; medium [scores 9–13]; high [scores 14–20])	3 FFQs One older and one newer 84-item FFQ and a more recent 65-item version	Cox proportional hazards models used to calculate HR (95% CIs) for cancer mortality Adjusted for socio-demographic, health, and health behaviour factors *F/U to 2007*	1 d–19 y F/U For every one point increase in LCHP score HR for cancer mortality risk = 1.00 (95% CI: 0.98, 1.03; P *tend* 0.851) for men. For women HR = 1.00 (95% CI: 0.97, 1.02; P *tend* 0.878)	No association between LCHP score in this study cohort and risk of mortality from all cancers
25	Reedy, J., et al., (2013) [76], Higher diet quality is associated with decreased risk of all-cause, cardiovascular disease, and cancer mortality among older adults	USA	Longitudinal cohort NIH-AARP	*N* = 492,623 men and women (aged 50–71 y at baseline in 1995–6)	HEI-2010 AHEI-2010 aMED (2) DASH score (1) All scores categorised into quintiles (Q1-Q5)	124-item FFQ completed at baseline *Validated*	Cox proportional hazards models used to calculate HR (95% CIs) for cancer mortality Adjusted for socio-demographic, health, and health behaviour factors *F/U to 2011*	Up to 15 y F/U HEI-2010 $HR_{(Q5VQ1)}$ = 0.76 (95% CI: 0.72, 0.80) * AHEI-2010 $HR_{(Q5VQ1)}$ = 0.82 (95% CI: 0.78, 0.86) aMED $HR_{(Q5VQ1)}$ = 0.80 (95% CI: 0.77, 0.84) DASH score $HR_{(Q5VQ1)}$ = 0.80 (95% CI: 0.76, 0.84)	Reduced risk of all cancer mortality was observed for this cohort across all 4 diet quality scores
26	Seymour, J.D., et al., (2003) [77], Diet quality index as a predictor of short-term mortality in the American cancer society cancer prevention study II nutrition cohort	USA	Longitudinal cohort American Cancer Society Cancer Prevention Study II Nutrition Cohort	*N* = 63,109 women *N* = 52,724 men Aged 50–70 y at baseline in 1992–3	DQI (1) Categorised into quintiles (Q1-Q5)	68-item FFQ completed at baseline in 1992–93	Cox proportional hazards models used to calculate RR (95% CIs) for cancer mortality Adjusted for socio-demographic, health, and health behaviour factors *F/U to 1996*	Up to 4 y F/U Cancer mortality Women $RR_{(Q5VQ1)}$ = 0.61 (95% CI: 0.32, 1.18; P *trend* 0.28) Men $RR_{(Q5VQ1)}$ = 0.92 (95% CI: 0.63, 1.34; P *trend* 0.28)	The DQI has limited ability to predict all-cause and cancer mortality

Table S3. *Cont.*

	Study	Location	Study Design & Cohort	Participants	Diet Quality Score	Method & Period of Dietary Analysis	Outcome Variable/s	Cancer Mortality Results *	Overall Conclusions
27	Tognon, G., et al., (2012) [78] The Mediterranean diet score and mortality are inversely associated in adults living in the subarctic region	Northern Sweden	Longitudinal cohort VIP	*N* = 77,151 men and women recruited from 1990–2008 (aged 30–60 y)	mMDS (2)	3 FFQs One older and one newer 84-item FFQ and a more recent 65-item version	Cox proportional hazards models used to calculate RR (95% CIs) for all cause and cancer mortality Adjusted for socio-demographic, health, and health behaviour factors *F/U to 2007*	1 d–19 y F/U For every one unit increase in mMDS: Men All-cause cancer: HR = 0.92 (95% CI: 0.87, 0.98.P *trend* < 0.01) Pancreatic cancer: HR = 0.82 (95% CI: 0.68, 0.99. P *trend* < 0.05) Colorectal cancer: HR = 1.07 (95% CI: 0.93, 1.24. P *trend* > 0.05) Stomach cancer: HR = 1.07 (95% CI: 0.85, 1.34. P *trend* > 0.05) Prostate cancer: HR = 0.88 (95% CI: 0.74, 1.03. P *trend* > 0.05) Women All-cause cancer: HR = 0.98 (95% CI: 0.92, 1.03. P *trend* > 0.05) Pancreatic cancer: HR = 0.83 (95% CI: 0.69, 1.00. P *trend* > 0.05) Colorectal cancer: HR = 0.91 (95% CI: 0.77, 1.06. P *trend* > 0.05) Stomach cancer: HR = 1.24 (95% CI: 0.95, 1.04. P *trend* > 0.05) Breast cancer: HR = 1.12 (95% CI: 0.97, 1.28. P *trend* > 0.05)	mMDS significantly related to all-cause mortality. mMDS associated with all-sites and pancreatic cancer mortality in men
28	Trichopoulou, A., et al., (2003) [79], Adherence to a Mediterranean die and survival in a Greek population	Greece	Longitudinal cohort EPIC	*N* = 22,043 men and women Aged 20–86 y at baseline in 1994–9	MMDS (1)	150-item FFQ collected data on yr prior to enrolment in study *Validated*	Cox proportional hazards regression for HR (95% CI's) for total and cause-specific mortality Adjusted for socio-demographic, health, and health behaviour factors *F/U to 2003*	Median F/U = 3.7 y Cancer mortality For every 2-point increment in MMDS: HR = 0.76 (0.59, 0.98)	Greater adherence to the traditional Mediterranean diet is associated with a significant reduction in total and cancer mortality
29	Van Dam, R.M., et al., (2008) [81], Combined impact of lifestyle factors on mortality: prospective cohort study in US women	USA	Longitudinal cohort NHS	*N* = 77,782 women aged 34–59 at baseline in 1980	AHEI (2) Categorised into quintiles (Q1-Q5)	61-item FFQ *Validated*	Pooled logistic regression analysis stratified by 2 year calendar time periods to estimate multivariate RR and 95% CI for risk of total and cause-specific mortality Adjusted for socio-demographic, health, and health behaviour factors *F/U to 2004*	Up to 24 y F/U Cancer mortality $RR_{(Q5\ V\ Q1)}$ = 0.72 (95% CI : 0.65, 0.79)	Higher healthy diet score associated with reduced risk of cancer mortality

Table S3. *Cont*

	Study	Location	Study Design & Cohort	Participants	Diet Quality Score	Method & Period of Dietary Analysis	Outcome Variable/s	Cancer Mortality Results *	Overall Conclusions
30	Vormund, K., et al., (2014) [82], Mediterranean diet and mortality in Switzerland: an alpine paradox?	Switzerland	Longitudinal cohort	*N* = 17,861 men (aged 44.9 ± 13.3 y) and women (aged 45.2 ± 13.6 y)	MDS (1) Presented as continuous variable and into three categories, low-high: <4; 4–5; >5	24 h recall (completed at baseline 1977–1993)	Cox proportional hazards regression for HR (95% CI's) for total and cause-specific mortality Adjusted for socio-demographic, health, and health behaviour factors *F/U to 2008*	μ F/U = 21.4 y<br>Cancer mortality<br>*Men*<br>For a 1 = pt increase in MDS HR = 0.92 (95% CI: 0.88, 0.97)<br>$HR_{(high\ V\ Low\ scores)}$ = 0.80 (95% CI: 0.65, 0.99)<br>*Women*<br>For a 1 = pt increase in MDS HR = 0.98 (95% CI: 0.93, 1.04)<br>$HR_{(high\ V\ Low\ scores)}$ = 0.92 (95% CI: 0.73, 1.17)<br>*Men and women*<br>For a 1 = pt increase in MDS HR = 0.95 (95% CI: 0.92, 0.99)<br>$HR_{(high\ V\ Low\ scores)}$ = 0.83 (95% CI: 0.70, 0.97)	Stronger adherence to Mediterranean diet and all cause as well as cancer risk was observed
31	Zarrin, R., et al., (2013) [84], Development and validity assessment of a diet quality index for Australians	Australia	Longitudinal cohort Nambour Skin Cancer study	*N* = 1355 men and women	Aussie-DQI Categorised into tertiles (T1–T3)	24 h recall (completed in 1995)	Cox proportional hazards regression for HR (95% CI's) for total and cause-specific mortality Adjusted for socio-demographic, health, and health behaviour factors *F/U to 2007*	Up to F/U 16 y<br>Cancer mortality<br>*Men*<br>$HR_{(tertile\ 3\ V\ tertile\ 1)}$ = 0.30 (95% CI: 0.11, 0.83; P *trend* 0.06)<br>*Women*<br>$HR_{(tertile\ 3\ V\ tertile\ 1)}$ = 0.64 (95% CI: 0.24, 1.68; P *trend* 0.65)	Adherence to the Aussie-DQI in men is associated with reduced cancer mortality

* Fully adjusted hazard ratio (HR) or relative risk (RR) reported where given.

References

93. Dixon, L.B.; Subar A.F.; Peters, U.; Weissfeld, J.L.; Bresalier, R.S.; Risch, A.; Schatzkin, A.; Hayes, R.B. Adherence to the USDA Food Guide, DASH Eating Plan, and Mediterranean dietary pattern reduces risk of colorectal adenoma. *J. Nutr*. **2007**, *137*, 2443–2450.
94. Drake, I.; Gullberg, B.; Ericson, U.; Sonestedt, E.; Nilsson, J.; Wallström, P.; Hedblad, B.; Wirfält, E. Development of a diet quality index assessing adherence to the Swedish nutrition recommendations and dietary guidelines in the Malmö Diet and Cancer cohort. *Public Health Nutr*. **2011**, *14*, 835–845.
95. Fung, T.T.; Chiuve, S.E.; McCullough, M.L.; Rexrode, K.M.; Logroscino, G.; Hu, F.B. Adherence to a DASH-style diet and risk of coronary heart disease and stroke in women. *Arch. Intern. Med*. **2008**, *168*, 713–720.
96. Guenther, P.M.; Casavale, K.O.; Reedy, J.; Kirkpatrick, S.I.; Hiza, H.A.; Kuczynski, K.J.; Kahle, L.L.; Krebs-Smith, S.M. Update of the healthy eating index: HEI-2010. *J. Acad. Nutr. Diet*. **2013**, *113*, 569–580.
97. Guenther, P.M.; Krebs-Smith, S.M.; Reedy, J.; Britten, P.; Juan, W.; Lino, M.; Carlson, A.; Hiza, H.A.; Basiotis, P.P. Healthy eating index-2005. *Center Nutr. Policy Promot. Factsheet*. **2006**,1.
98. Günther, A.L.; Liese, A.D.; Bell, R.A.; Dabelea, D.; Lawrence, J.M.; Rodriguez, B.L.; Standiford, D.A.; Mayer-Davis, E.J. Association between the dietary approaches to hypertension diet and hypertension in youth with diabetes mellitus. *Hypertension* **2009**, *53*, 6–12.
99. Haines, P.S.; Siega-Riz, A.M.; Popkin, B.M. The Diet Quality Index revised: A measurement instrument for populations. *J. Am. Diet. Assoc*. **1999**, *99*, 697–704.
100. Kant, A.K.; Block, G.; Schatzkin, A.; Ziegler, R.G.; Nestle, M. Dietary diversity in the US population, NHANES II, 1976–1980. *J. Am. Diet. Assoc*. **1991**, *91*, 1526–1531.
101. Mellen P.B.; Gao S.K.; Vitolins M.Z.; Goff D.C. Deteriorating dietary habits among adults with hypertension: DASH dietary accordance, NHANES 1988-1994 and 1999-2004. *Arch. Intern. Med*. 2008, *168*, 308–314.
102. Olsen, A.; Egeberg, R.; Halkjær, J.; Christensen, J.; Overvad, K.; Tjønneland, A. Healthy aspects of the Nordic diet are related to lower total mortality. *J. Nutr*. **2011**, *141*, 639–644.
103. Tkennedy, E.; Ohls, J.; Carlson, S.; Fleming, K. The healthy eating index: Design and applications. *J. Am. Diet. Assoc*. **1995**, *95*, 1103–1108.
104. Trichopoulou, A.; Psaltopoulou, T.; Orfanos, P.; Hsieh, C.; Trichopoulos, D. Low-carbohydrate–high-protein diet and long-term survival in a general population cohort. *Eur. J. Clin. Nutr*. **2007**, *61*, 575–581.
